# The functional impact of myofiber macroscopic organization and disarray in computational models of the murine heart

**DOI:** 10.1371/journal.pcbi.1014807

**Published:** 2026-09-21

**Authors:** Carlo Guastamacchia, Roberto Piersanti, Francesco Giardini, Raffaele Coppini, Cecilia Ferrantini, Luca Dede’, Leonardo Sacconi, Francesco Regazzoni

**Affiliations:** 1 MOX - Department of Mathematics, Politecnico di Milano, Milan, Italy; 2 Department of Theoretical and Applied Sciences, eCampus University, Novedrate, Italy; 3 SMARTEST Research Center, eCampus University, Novedrate, Italy; 4 Institute for Experimental Cardiovascular Medicine, University Heart Center Freiburg – Bad Krozingen, Medical Faculty and Medical Center – University of Freiburg, Freiburg im Breisgau, Germany; 5 Department of Experimental and Clinical Medicine, University of Florence, Florence, Italy; 6 Institute of Clinical Physiology, National Research Council (IFC-CNR), Florence, Italy; Nanjing University, CHINA

## Abstract

A major challenge in computational models of cardiac electromechanics is the reconstruction of myocardial fiber architecture, as direct in vivo measurements of fiber orientation are not feasible. Consequently, rule-based methods are commonly adopted as surrogates, relying on empirical descriptions of fiber organization combined with patient-specific geometries. This study investigates the respective roles of macroscopic fiber architecture and microscopic fiber disarray in cardiac electromechanical simulations. A high-fidelity biventricular electromechanical model of a murine heart was developed using a high-resolution myocardial fiber field obtained via mesoscopic optical imaging, which serves as a reference ground truth. A spatial smoothing strategy is introduced to decouple macroscopic fiber organization from local disarray, and the resulting responses are also compared with those obtained using a rule-based fiber field. The results show that passive mechanics and electrophysiological activation are only weakly affected by fiber disarray, with global chamber compliance and activation times remaining largely unchanged across different fiber descriptions. In contrast, active mechanics is highly sensitive to fiber architecture. Moderate regularization of the experimentally measured fiber field enhances the ventricular pumping efficiency of the computational model by reducing microscopic disarray while preserving the macroscopic helical organization, whereas excessive smoothing or rule-based fiber reconstructions lead to unphysiologically strong or inefficient contraction. Within this framework, two commonly adopted surrogate strategies to account for fiber disarray are investigated: (i) a reduction of the effective cross-bridge stiffness in the active tension model, and (ii) the introduction of controlled misalignment between active tension and the local fiber direction. While both approaches reproduce global hemodynamic indicators comparable to the reference case, an effective reduction of contractility – despite its phenomenological nature – provides a closer match to the reference strain patterns than the introduction of orthogonal active stress components. Overall, the results highlight the dominant role of macroscopic fiber architecture in active mechanics and reveal important limitations of commonly adopted surrogate approaches for modeling fiber disarray.

## Author summary

To integrate cardiac computational models into routine clinical practice, it is essential to validate the modeling strategies proposed in the scientific literature. A major challenge in this context is the reconstruction of myocardial fiber fields, as obtaining precise in-vivo measurements of fiber orientation within patient-specific organs remains infeasible. An additional source of complexity arises from fiber disarray, which refers to local deviations of fiber orientation from the average direction.

Current state-of-the-art methods for reconstructing cardiac fiber fields from heart geometry typically neglect the presence of fiber disarray. In this work, we analyze a dataset containing high-resolution fiber information obtained from ex-vivo measurements of a mouse heart. We introduce a methodology to assess the relative impact of the mean fiber direction and fiber disarray on electromechanical simulations, and we evaluate the reliability of a state-of-the-art fiber surrogation approach.

Our results indicate that fiber orientation has only a limited effect on electrical signal propagation and on the passive mechanical properties of the heart. However, when active tension generated by myocardial fibers is included in the model, fiber orientation and disarray have a strong influence on cardiac contraction and local tissue deformations. Furthermore, the results show that commonly used surrogate fiber models, even when combined with specific modeling techniques to account for disarray, are not sufficient to accurately reproduce the local effects associated with the real fiber field.

## 1 Introduction

Cardiac computational models are increasingly being used in modern medical practice [1,2]. These models have demonstrated the ability to reproduce action potential propagation under both physiological [3,4] and pathological conditions [5–8], to simulate mechanical contraction in electromechanical frameworks [9–11], and to model blood flow in fluid dynamic simulations [12]. By leveraging these models, it is possible to investigate and predict specific quantities of interest [13], prognosticate disease evolution [14], and test new medical treatments in silico [15]. The ultimate goal in this field is the development of patient-specific digital twins capable of delivering personalized analyses and therapeutic strategies [16–22]. Achieving this objective requires an accurate representation of the heart’s muscles structure and function; however, with current technological capabilities, such detailed reconstruction remains unfeasible on a patient specific basis [23].

The cardiac muscle is composed of cardiomyocytes arranged into fibers, commonly referred to as myofibers. As described by [24], the myocardial fiber architecture consists of an orderly laminar organization of myofibers, characterized by extensive cleavage planes separating adjacent muscle layers. In transmural sections, these planes extended radially from the endocardium (the inner surface) to the epicardium (the outer surface), aligning with the local myofiber orientation observed in tangential sections. This well-organized laminar architecture can be described in terms of three material symmetry axes: the logitudinal, the sheet, and the normal directions. The longitudinal axes of myocytes, which constitute laminae, show a well-defined helical organization that progressively rotates along the transmural axis from the endocardium to the epicardium [25]. However, the complexity of the myofiber architecture increases when considering the heterogeneity in macroscopic fiber orientation and the presence of physiological disarray [26]. The disarray of fibers consists in a local misalignment of the fibers with respect to the mean direction [27].

The characteristic myofiber configuration has a major impact on the heart function. Fiber orientation affects action potential propagation within the muscle, as the spread of epicardial excitation is considerably faster parallel to the longitudinal axes of the cardiac fibers than perpendicular to them [28]. Furthermore, fiber disarray can alter tissue electrical conductivity, leading to a more isotropic propagation of the wavefront [29]. In addition, the tissue’s mechanical contraction, driven by the propagation of electrical signals, is highly dependent on the alignment of the muscle fibers [30]. Due to its anisotropic nature, cardiac muscle exhibits direction-dependent material stiffness determined by the local myofiber architecture along the three principal directions [31]. Morover, cardiomyocytes are also responsible for active contraction, producing force mainly aligned with the fiber orientation [32]. Since myofibers play a pivotal role in cardiac function – affecting electrophysiological behavior, passive mechanical properties, and active contraction – precise representation of their spatial architecture is crucial in cardiac computational modeling. In particular, cardiac digital twins require to represent patient-specific fiber architectures [23,33]. Furthermore, assessing the impact of fiber and disarray is key to unraveling the complex pathophysiological mechanisms underlying the cardiac diseases, such as hypercontractility, hypocontractility, and fibrosis [7].

The current *de facto* standard imaging technique for reconstructing the fiber architecture is Diffusion Tensor Magnetic Resonance Imaging (DTMRI) [34,35], which has achieved resolutions of 400 μm in ex-vivo human hearts [36] and 43 μm in ex-vivo mouse hearts [37]. However, DTMRI suffers from poor signal-to-noise ratio and long acquisition times [38]. As an alternative, micro-computed tomography [39], which does not require long preparation or measurement times, has reached a resolution of 10 μm in ex-vivo rat hearts. More recently, the authors of [40] demonstrated that, by means of Hierarchical Phase-Contrast Tomography (HiP-CT), it is possible to reconstruct an entire human heart at an isotropic resolution of approximately 25 μm per voxel, while integrating hierarchical local scans down to ~2 μm per voxel for the detailed analysis of cardiac musculature and cardiomyocyte organization. Nevertheless, the applicability of such approaches is limited by the high cost of synchrotrons for X-ray production [41] which severely restricts their accessibility. Another imaging technique is shear-wave imaging, which achieved a resolution of 200 μm in ex-vivo porcine hearts [42]. It is worth noting that optical imaging techniques can achieve micrometric or even sub-micrometric resolution; however, this is typically limited to thin tissue sections or optically cleared samples with constrained thickness [43], and does not allow imaging of the intact whole organ at comparable resolution. To overcome the aforementioned limitations, recent advances in optical imaging techniques [44] have enabled mesoscale reconstruction of cardiac anatomy at the whole-organ level. In particular, the combination of tissue clearing methods with a new generation of mesoSPIM microscopes enables whole-heart reconstruction at an isotropic resolution of 3.25 μm × 3.00 μm in ex vivo mouse hearts, as demonstrated by [45]. All of the imaging techniques mentioned above are performed ex vivo. Currently, in vivo fiber identification remains limited by their relatively coarse spatial resolution [46–49]. Therefore, contemporary fiber-imaging techniques are largely impractical for building patient-specific cardiac computational models.

Given the challenges associated with obtaining patient-specific fiber fields, mathematical models, known as Rule-Based Methods (RBMs) are commonly employed in cardiac computational models. RBMs surrogate the characteristic myocardial fiber architecture. RBMs represent fiber orientations exploiting mathematically sound rules informed by histological or imaging data together with patient-specific cardiac geometry [23,50–54]. The current state-of-the-art of RBMs is represented by the Laplace-Dirichlet Rule Based Methods (LDRBMs), which determine the myofiber direction by solving suitable Laplace-Dirichlet problems. Despite their widespread use, the impact of these methods on the reliability of models has yet to be definitively established. In [52], the authors compared the fiber fields generated by ventricular LDRBM with those obtained from diffusion tensor magnetic resonance imaging (DTMRI) at a spatial resolution of 400 μm. The study reported a non-negligible angular discrepancy of approximately 30 °. From an electrophysiological perspective, the works in [51,52] validated ventricular LDRBMs by comparing simulated activation maps with experimental measurements. The method proved effective in reproducing activation patterns across biventricular geometries. Specific RBMs have been developed for atria as they present more complex fiber architecture characterized by bundles with different mean fiber directions [50,55,56]. Nevertheless, the analysis of these models is out of the scope of this work.

Still, should an exact representation of every single cardiomyocyte orientation be available, the computational cost of running a simulation of the full organ by resolving the cell scale would be unaffordable. As a matter of fact, attempts to simulate the cardiac function at the cell scale remain limited to specimens composed by a few cells [57–59]. Most of cardiac computational models are in fact homogenized, as they describe the tissue as a continuum, without resolving the single cells. In such models, fiber disarray is not explicitly represented, but it is implicitly incorporated either through effective parameters or through purposely defined corrective terms. This necessity is inherently resolution-dependent: when the computational mesh does not resolve local variations in fiber orientation, the effect of sub-grid fiber disarray should be incorporated through effective tissue properties. Conversely, at sufficiently high spatial resolution, local fiber perturbations may in principle be represented explicitly, either from direct measurements or through stochastic perturbations calibrated from high-resolution imaging data. However, resolving such fine-scale variability in full-organ electromechanical simulations may rapidly become computationally prohibitive, thereby motivating the use of homogenized representations in large-scale cardiac models. In particular, in electrophysiology models, fiber disarray is implicitly accounted for in the definition of the anisotropic diffusion tensor. Similarly, in passive mechanics models, fiber disarray is encoded in the coefficients of the hyperelastic constitutive law [60–62]. Indeed, experimental tissue samples used for mechanical testing and models calibration inherently exhibit fibers with some degree of disarray [63]. Conversely, in active mechanics, a dedicated modeling strategy is required to account for the influence of fiber disarray [64–66]. To this end, the authors of [67] proposed introducing cross-fiber activation to mimic the effects of fiber disarray within RBM frameworks. In [53], RBMs were employed in biventricular electromechanical simulations that successfully reproduced realistic contraction patterns. In [68], biventricular electromechanical simulations incorporating cross-fiber active tension successfully reproduced experimental data of key mechanical biomarkers reported in the literature under physiological conditions. However, in both studies, no experimental ground truth was available for validation. The authors in [69,70] developed a mechanical model of a biventricular geometry, comparing simulations based on RBM-generated fibers with those using experimentally measured ones. The study showed that incorporating cross-fiber activation improved the agreement of pressure–volume (PV) loops with experimental data but failed to fully capture local deformation effects. However, this analysis, conducted on porcine heart model embedded with canine fibers, is limited by the absence of coupling with an electrophysiology model. In conclusion, to the best of our knowledge, no validation of RBMs using comprehensive electromechanical models and high-resolution fiber data has yet been reported in the literature.

Despite the non conclusive literature results, cross-fiber activation remains a widely adopted approach in standard electromechanical simulations employing RBMs [68,71–73]. Moreover, while the sensitivity of mechanical [74] and electromechanical [68,69,75,76] simulations to fiber orientation has been shown in literature, the specific impact of fiber disarray with respect to the mean fiber orientation has, to the best of our knowledge, not yet been explored.

Motivated by these open issues, in this work we present a computational study based on a measured myofiber field obtained using mesoscopic optical imaging [77] in a murine heart, enabling an electromechanical analysis at unprecedented spatial resolution. The different components of the model are calibrated using functional measurements acquired from the same specimen, including activation maps, while complementary information is drawn from the literature when direct measurements are not available. To independently assess the functional impact of macroscopic fiber architecture and microscopic fiber disarray, we introduce a methodology based on spatial frequency decomposition that allows these two contributions to be disentangled. Leveraging the very high resolution and signal-to-noise ratio of the measured fiber field, together with the proposed disentangling approach, this study enables multiple analyses. First, we investigate the respective effects of macroscopic architecture and microscopic disarray on cardiac function, spanning passive mechanics, electrophysiology, and active contraction. Second, we provide a validation of commonly adopted modeling strategies used in practice to account for these features, namely RBM on the one hand, and the inclusion of cross-fiber activation or modulation of cross-bridge stiffness on the other.

The remainder of the paper is organized as follows. In Sec. 2, we present the methods used to characterize the macroscopic fiber architecture and local disarray, as well as the LDRBM considered in this work. In Sec. 2.5, we describe the electromechanical model used to study the effect of fiber architecture on cardiac electromechanics. In Sec. 3, we present the results, while Sec. 4 is devoted to their discussion.

## 2 Materials and methods

In this section, we introduce the notation employed to characterize the reference system and the angles defining the fibers architecture. Then we describe the experimental data and the procedure used to isolate the macroscopic fiber architecture from the microscopic disarray. In addition, we briefly describe the LDRBM considered in this work. Finally, we provide a short overview of the electromechanical model we implemented.

### 2.1 Fiber architecture and rotation angles

**Fig 1:**
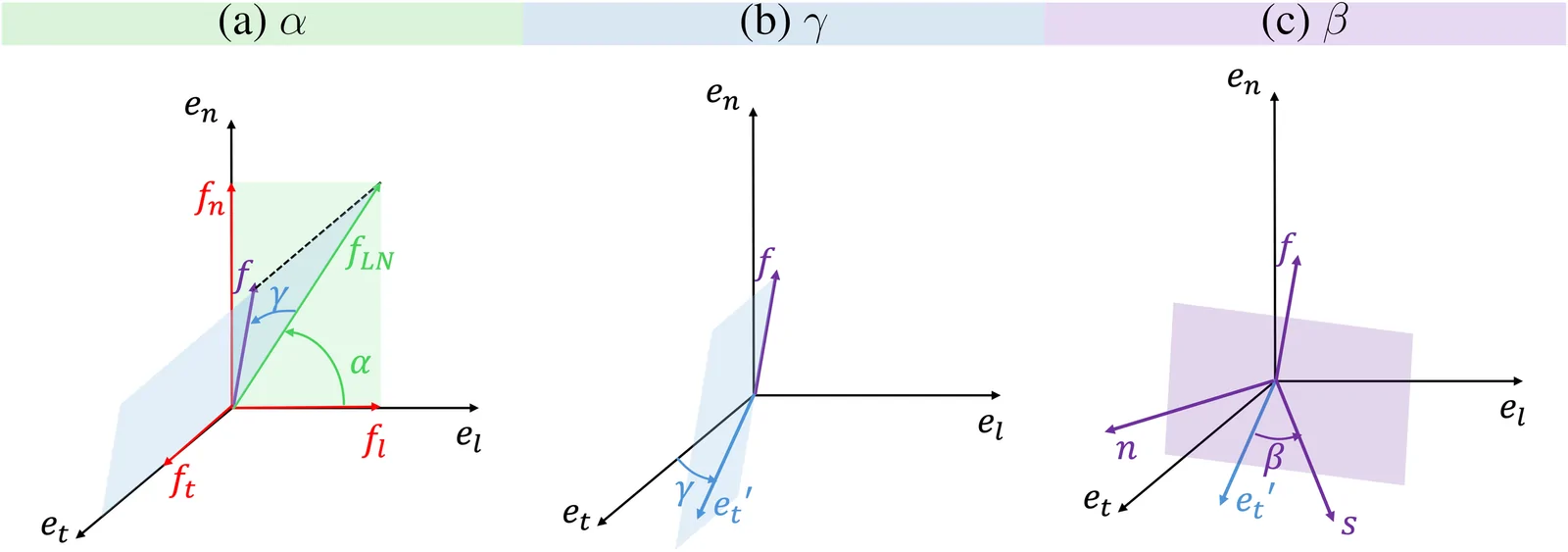
From myocardial to fiber reference. **(a)** Rotation of ${𝐞}_{l}$ by an angle α around the axis ${𝐞}_{t}$ to obtain ${𝐟}_{LN}$, followed by a rotation of ${𝐟}_{LN}$ by an angle γ in the plane $\text{span}\{{𝐟}_{LN},{𝐞}_{t}\}$ (depicted in blue) to obtain 𝐟. **(b)** Rotation of ${𝐞}_{t}$ by an angle γ to obtain ${𝐞}_{t}^{\prime }$. **(c)** Rotation of ${𝐞}_{t}^{\prime }$ by an angle β around 𝐟 (in the lilac plane) to obtain 𝐬, and definition of 𝐧 as the normal to both 𝐟 and 𝐬.

The fiber field is defined by three principal orthogonal directions, which characterize the tissue properties: 𝐟 is the longitudinal fiber direction; 𝐬 is the transmural sheet direction defining the orthogonal direction to the fiber and lying on the fiber’s sheet plane; 𝐧 is the normal direction to the sheet plane. These three orthogonal directions compose the fiber reference system. In modeling practice, an additional triplet known as the myocardial reference system is often introduced [50–53]. Its formulation relies solely on information about myocardial geometry and is designed to parametrize the transmural and apico-basal directions throughout the entire myocardium, thereby defining an orthotropic reference frame. Consequently, the myocardial reference frame can be used to analyze fiber orientation with respect to myocardial geometry or to surrogate the fiber field through RBMs [23,50]. The three orthogonal directions of the myocardial reference system are: the apico-basal direction ${𝐞}_{n}$, going from the ventricular apex to the base; the transmural direction ${𝐞}_{t}$, going from the endocardium to the epicardium; the circumferential direction ${𝐞}_{l}$, defined as normal to the other two. The fiber’s principal directions (𝐟, 𝐬, 𝐧) and the myocardial reference system (${𝐞}_{l}$, ${𝐞}_{t}$, ${𝐞}_{n}$) are linked by rotation angles. These allow to pass from one reference system to the other. In particular, three rotation angles are required to map the myocardial reference system into the fiber reference system [78]: the projected helical angle α, that is the angle between the projection of the longitudinal fiber direction 𝐟 on the epicardial tangential plane defined by the circumferential and apico-basal directions ${𝐞}_{l}$ and ${𝐞}_{n}$; the intrusion angle γ, defined as the one between ${𝐞}_{l}$ and the plane ${𝐞}_{l}\text{-}{𝐞}_{n}$; the cross-fiber angle β defined as the rotation of the system around the ${𝐞}_{l}$ direction (see Fig 1).

In the following paragraphs, we describe how the myocardial reference system is defined, how to compute the rotation angles from the experimental fiber reference system, and, vice-versa, how to reconstruct the fiber reference system from the angles.

#### 2.1.1 Myocardial reference system.

**Fig 2:**
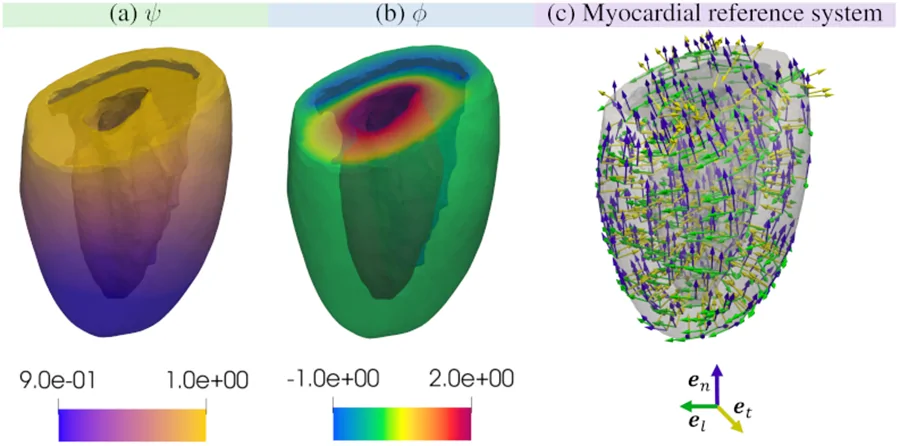
Construction of the myocardial reference system. (a) apico-basal coordinate ψ (b) transmural coordinate ϕ, (c) myocardial reference system. The colormap for the apico-basal coordinate was saturated in the 0.9–1.0 range to better highlight its variation across the myocardium.

Several LDRBMs have been proposed in the literature, each employing different strategies to compute the myocardial reference system [51–53]. Recently, a unified mathematical framework encompassing many of the existing LDRBM approaches has been proposed [50]. In this work, we use a LDRBM based on Doste et al. [52], with the modifications presented in [50], that enable its application to based biventricular geometries. Here, based refers to the artificial truncation of the ventricular geometry by a basal plane, introduced to define the ventricular base as a boundary of the computational domain. We emphasize that the Doste et al. RBM provides a dedicated treatment of specific ventricular regions, such as the inter-ventricular septum (approximately assigning two-thirds of the septum to LV and one-third to RV) as well as tailored fiber orientations for the RV. In the adopted formulation, the three directions ${𝐞}_{l}$, ${𝐞}_{t}$, and ${𝐞}_{n}$ are computed from two scalar fields: the transmural coordinate ϕ which surrogates the distance from the epicardium and the endocardial surfaces, see Fig 2(a); the apico-basal ψ coordinate representing the position with respect to the apex and the ventricular base, see Fig 2(b). Finally, the gradients of the transmural coordinate ϕ and the apico-basal coordinate ψ are used to construct the transmural and apico-basal directions, respectively.

The scalar fields ϕ and ψ are computed by solving the following problems


$$\begin{array}{ccc} \left\{ \begin{array}{cccc} & \Delta \phi =0 & & \text{ in }\Omega \\ & \phi =0 & & \text{ on }\Gamma_{Epi},\\ & \phi =-1 & & \text{ on }\Gamma_{Endo,RV},\\ & \phi =2 & & \text{ on }\Gamma_{Endo,LV}, \end{array}\right. & & \left\{ \begin{array}{cccc} & \Delta \psi =0 & & \text{ in }\Omega \\ & \psi =0 & & \text{ on }\Gamma_{Apex},\\ & \psi =1 & & \text{ on }\Gamma_{Base}, \end{array}\right. \end{array}$$ (1)

where Ω is the computational domain; $\Gamma_{Epi}$, $\Gamma_{Endo,RV}$ and $\Gamma_{Endo,LV}$ represent the epicardium, the endocardium of the Right Ventricle (RV) and the endocardium of the Left Ventricle (LV), respectively; while $\Gamma_{Base}$ represents the ventricular base and $\Gamma_{Apex}$ the apex of the myocardium as shown in S7 Fig. Given the variables ϕ and ψ, the transmural, apico-basal, and the circumferential directions ${𝐞}_{t}$, ${𝐞}_{n}$, and ${𝐞}_{l}$ are computed according to


$$\begin{array}{c} \left\{ \begin{array}{c} {𝐞}_{t}=\frac{\nabla \phi }{\left\|\nabla \phi \right\|},\\ {}[4pt]{𝐞}_{n}=\frac{\frac{\nabla \psi }{\left\|\nabla \psi \right\|}-\left(\frac{\nabla \psi }{\left\|\nabla \psi \right\|}\cdot {𝐞}_{t}\right){𝐞}_{t}}{\left\|\frac{\nabla \psi }{\left\|\nabla \psi \right\|}-\left(\frac{\nabla \psi }{\left\|\nabla \psi \right\|}\cdot {𝐞}_{t}\right){𝐞}_{t}\right\|},\\ {}[4pt]{𝐞}_{l}={𝐞}_{n}\times {𝐞}_{t}. \end{array}\right. \end{array}$$ (2)

Fig 2 illustrates the transmural coordinate ϕ, the apico-basal coordinate ψ, and the myocardial reference system (${𝐞}_{l}$, ${𝐞}_{t}$, ${𝐞}_{n}$).

#### 2.1.2 From fibers to angles.

When both the fiber field and the myocardial reference system are available, the rotation angles α, γ, and β can be computed in sequence. To obtain α, the longitudinal direction 𝐟 is projected on the plane ${𝐞}_{n}\text{-}{𝐞}_{l}$, resulting in ${𝐟}_{LN}$ as


$$\begin{array}{c} {𝐟}_{LN}=𝐟-(𝐟\cdot {𝐞}_{t}){𝐞}_{t}, \end{array}$$ (3)

and than normalized in ${\hat{𝐟}}_{LN}$


$$\begin{array}{c} {\hat{𝐟}}_{LN}=\frac{{𝐟}_{LN}}{\left\|{𝐟}_{LN}\right\|}. \end{array}$$ (4)

After defining ${\hat{𝐟}}_{LN}$, α is obtained as the angle between ${\hat{𝐟}}_{LN}$ and ${𝐞}_{l}$, with α∈(−π,π]. This choice avoids treating angles α and α+2π as distinct. Finally, the atan2 function is used to compute α


$$\begin{array}{c} \alpha =atan2({𝐞}_{n}\cdot {\hat{𝐟}}_{LN},{𝐞}_{l}\cdot {\hat{𝐟}}_{LN}), \end{array}$$ (5)

where the sign of the dot product ${𝐞}_{l}\cdot {\hat{𝐟}}_{LN}$ specifies which half-plane contains ${\hat{𝐟}}_{LN}$. Similarly, γ is computed by inverting the sign for |α|>π/2:


$$\begin{array}{c} \gamma =\left\{ \begin{array}{cc} atan2({𝐞}_{t}\cdot 𝐟,𝐟\cdot {\hat{𝐟}}_{LN}) & \text{ if }|\alpha |\leq \pi /2,\\ -atan2({𝐞}_{t}\cdot 𝐟,𝐟\cdot {\hat{𝐟}}_{LN}) & \text{ if }|\alpha |>\pi /2. \end{array}\right. \end{array}$$ (6)

To compute β, two auxiliary vectors are introduced: ${𝐞}_{t}^{\prime }$, which is the transmural ${𝐞}_{t}$ direction rotated of γ in the $𝐟\text{-}{𝐞}_{t}$ plane, and ${𝐞}_{n}^{\prime }$ which is the normal direction to the $𝐟\text{-}{𝐞}_{t}$ plane. Specifically, ${𝐞}_{t}^{\prime }$ and ${𝐞}_{n}^{\prime }$ are computed as


$$\begin{array}{c} \left\{ \begin{array}{c} {𝐞}_{t}^{\prime }=\frac{{𝐞}_{t}-({𝐞}_{t}\cdot 𝐟)𝐟}{\left\|{𝐞}_{t}-({𝐞}_{t}\cdot 𝐟)𝐟\right\|},\\ {}[8pt]{𝐞}_{n}^{\prime }=\frac{𝐟\times {𝐞}_{t}}{\left\|𝐟\times {𝐞}_{t}\right\|}. \end{array}\right. \end{array}$$ (7)

Finally, the β angle is evaluated as


$$\begin{array}{c} \beta =\left\{ \begin{array}{cc} atan2({𝐞}_{t}^{\prime }\cdot 𝐬,𝐬\cdot {𝐞}_{n}^{\prime }) & \text{ if }\alpha \geq 0,\\ -atan2({𝐞}_{t}^{\prime }\cdot 𝐬,𝐬\cdot {𝐞}_{n}^{\prime }) & \text{ if }\alpha <0. \end{array}\right. \end{array}$$ (8)

Fig 1 depicts the three rotations.

#### 2.1.3 From angles to fibers.

When the angles are provided – either measured experimentally from fiber orientations or computed by a LDRBM – the myofiber field is reconstructed via the inverse process, which converts the angles back to the fiber reference system. To compute the fiber reference system 𝐟, 𝐬, 𝐧, from the values of α and γ two rotations are imposed: the first, around direction ${𝐞}_{t}$, is used to compute ${𝐟}_{LN}$; the second, on the ${𝐟}_{LN}\text{-}𝐬$ plane, maps ${𝐟}_{LN}$ into 𝐟 and ${𝐞}_{t}$ into ${𝐞}_{t}^{\prime }$. In addition, ${𝐞}_{n}^{\prime }$ is defined as the cross product of ${𝐞}_{t}^{\prime }$ and 𝐟 as follows


$$\begin{array}{c} \left\{ \begin{array}{c} 𝐟=\cos(\alpha )\cos(\gamma )\;{𝐞}_{l}+\sin(\alpha )\cos(\gamma )\;{𝐞}_{n}+\sin(\gamma )\;{𝐞}_{t},\\ {}[4pt]{𝐞}_{t}^{\prime }=\frac{{𝐞}_{t}-\sin(\gamma )\;𝐟}{\left\|{𝐞}_{t}-\sin(\gamma )\;𝐟\right\|},\\ {}[8pt]{𝐞}_{n}^{\prime }={𝐞}_{t}^{\prime }\times 𝐟. \end{array}\right. \end{array}$$ (9)

If β=0, then $𝐬={𝐞}_{t}^{\prime }$ and $𝐧={𝐞}_{n}^{\prime }$, otherwise if β≠0 the rotation around the longitudinal direction 𝐟 have to be considered by multiplying the fiber reference system with the rotation matrix ${𝐑}_{\beta }$ as follows


$$\begin{array}{c} {}[\begin{array}{c} 𝐟\\ {𝐞}_{t}^{\prime }\\ {𝐞}_{n}^{\prime } \end{array}]={𝐑}_{\beta }[\begin{array}{c} 𝐟\\ 𝐬\\ 𝐧 \end{array}], \end{array}$$ (10)

where ${𝐑}_{\beta }$ is defined as


$$\begin{array}{c} {𝐑}_{\beta }=[\begin{array}{cccc} & 1 & 0 & 0\\ & 0 & \cos(\beta ) & -\sin(\beta )\\ & 0 & \sin(\beta ) & \cos(\beta ) \end{array}]. \end{array}$$ (11)

### 2.2 Fibers measurement

The data used in this work have been measured by the optical imaging technique proposed in [45]. This approach provides high-resolution information on the three-dimensional organization of the myocardium, including the longitudinal and sheet directions of cardiomyocytes, across the entire mouse heart. Briefly, whole mouse hearts were fixed and optically cleared using the SHIELD protocol optimized for cardiac tissue [79]. Cleared hearts were imaged using a modified mesoscopic selective plane illumination microscope (mesoSPIM), exploiting the intrinsic autofluorescence of cardiac muscle to reconstruct the myocardial architecture at near-isotropic spatial resolution (3.25 μm×3.25 μm×3 μm) [45]. The resulting volumetric datasets allowed a detailed three-dimensional reconstruction of the myocardium at single-cell scale. Local cardiomyocyte orientation was then quantified with an isotropic spatial resolution of 96 μm by applying the Structure Tensor Analysis (STA) to the autofluorescence signal: the longitudinal myofiber field and the sheet direction were obtained from the eigenvectors associated with the smallest and the second smallest eigenvalues, respectively. The resulting STA-based mapping represents myocardial organization through two unit-length vector fields, describing the longitudinal myofiber field and the sheet orientation field.

### 2.3 Decoupling macroscopic fiber orientations from microscopic disarray

In this section, we present the methodology employed to derive the main fiber organization from an experimentally measured myofiber field, by separating macroscopic architecture from microscopic fiber disarray. The underlying idea is to perform a decoupling in the spatial frequency domain, treating high-frequency components as indicative of fiber disarray, while associating the remaining low-frequency content with the macroscopic fiber architecture. To this end, a Helmholtz filter is applied to the fiber field. This filter is particularly well suited for data defined on unstructured meshes and irregular domains, as encountered in cardiac geometries. Acting as a low-pass spatial filter, the Helmholtz filter attenuates high-frequency variations in fiber orientation, thereby removing fiber disarray. The resulting smoothed field is interpreted as the macroscopic fiber architecture, while the fiber disarray is subsequently defined as the residual signal.

This procedure can be applied independently to the three angles α, β, and γ. For the sake of simplicity, in what follows we focus on the α angle. The measured angle field α(𝐱) is decomposed into a macroscopic component $\tilde{\alpha }(𝐱,\ell )$ and a microscopic disarray term $\varepsilon_{\alpha }(𝐱,\ell )$:


$$\begin{array}{c} \alpha (𝐱)=\tilde{\alpha }(𝐱,\ell )+\varepsilon_{\alpha }(𝐱,\ell ), \end{array}$$ (12)

where, ℓ denotes the regularization radius representing the characteristic length scale of the smoothing operator used to extract the macroscopic field.

A critical issue is that direct smoothing in the angular domain is not straightforward for two primary reasons. First, angular variables are circular, meaning that α and α+2π represent the same value. Second, due to the directional invariance of the fibers, angles differing by π represent the same physical fiber direction, and therefore α and α+π should be treated as equivalent. For these reasons, smoothing is not performed directly on α, but rather in a transformed coordinate system that correctly reflects this topology. Specifically, the fiber angles are mapped onto the coordinate system $\left(\eta_{\sin},\eta_{\cos}\right)$, defined as


$$\begin{array}{c} \left\{ \begin{array}{c} \eta_{\sin}=\sin(2\alpha ),\\ \eta_{\cos}=\cos(2\alpha ). \end{array}\right. \end{array}$$ (13)

Since sin(2α)=sin(2(α+π)) and cos(2α)=cos(2(α+π)), opposite fiber directions are mapped to the same point in this coordinate system.

By applying a Helmholtz filter with regularization radius ℓ, the regularized coordinates ${\tilde{\eta }}_{\sin}$ and ${\tilde{\eta }}_{\cos}$ are obtained by solving the problems:


$$\begin{array}{c} \left\{ \begin{array}{cc} -\ell^{2}\Delta {\tilde{\eta }}_{\sin}+{\tilde{\eta }}_{\sin}=\eta_{\sin} & \text{ in }\Omega ,\\ \nabla {\tilde{\eta }}_{\sin}\cdot n_{\Gamma }=0 & \text{ on }\Gamma , \end{array}\right.\text{ and }\left\{ \begin{array}{cc} -\ell^{2}\Delta {\tilde{\eta }}_{\cos}+{\tilde{\eta }}_{\cos}=\eta_{\cos} & \text{ in }\Omega ,\\ \nabla {\tilde{\eta }}_{\cos}\cdot n_{\Gamma }=0 & \text{ on }\Gamma , \end{array}\right. \end{array}$$ (14)

where Γ is the whole boundary of the biventricular domain Ω. In an unbounded domain, each problem of Eq. (14) is equivalent to applying the convolution operator


$$\begin{array}{c} \tilde{\eta }(𝐱)=(\eta *h)(𝐱)=\int_{\Omega }h\;\left(|𝐱-{𝐱}^{\prime }|\right)\eta ({𝐱}^{\prime })\;d{𝐱}^{\prime }, \end{array}$$ (15)

where *h*(*r*) is the Green’s function of the operator $I-\ell^{2}\Delta$, given by


$$\begin{array}{c} h(r)=\frac{1}{4\pi \ell^{2}}\frac{e^{-r/\ell }}{r}. \end{array}$$ (16)

As shown in Eq. (16), the parameter ℓ defines the characteristic smoothing length and controls the attenuation of spatial frequencies significantly higher than 1/ℓ, effectively acting as a low-pass spatial filter. In practice, Eq. (14) is solved numerically using the finite element method, which is well suited for unstructured meshes and irregular computational domains.

Finally, the smoothed angle field $\tilde{\alpha }$ (in radians) is recovered by mapping the regularized coordinates back to the angular domain using the atan2 operator:


$$\begin{array}{c} \tilde{\alpha }=\frac{1}{2}atan2({\tilde{\eta }}_{\sin},{\tilde{\eta }}_{\cos}). \end{array}$$ (17)

After obtaining the regularized angle field, the fiber disarray $\varepsilon_{\alpha }$ is determined directly from Eq. (12).

As mentioned, the same procedure can be applied to all three angles α, β, and γ. However, in the present work, due to the transversely isotropic behavior of the material, rotations in the 𝐬–𝐧 plane, represented by the angle β, are neglected. Therefore, after computing $\tilde{\alpha }$ and $\tilde{\gamma }$, the regularized fiber reference system $\tilde{𝐟}$, $\tilde{𝐬}$, and $\tilde{𝐧}$ is reconstructed using Eq. (9).

**Fig 3:**
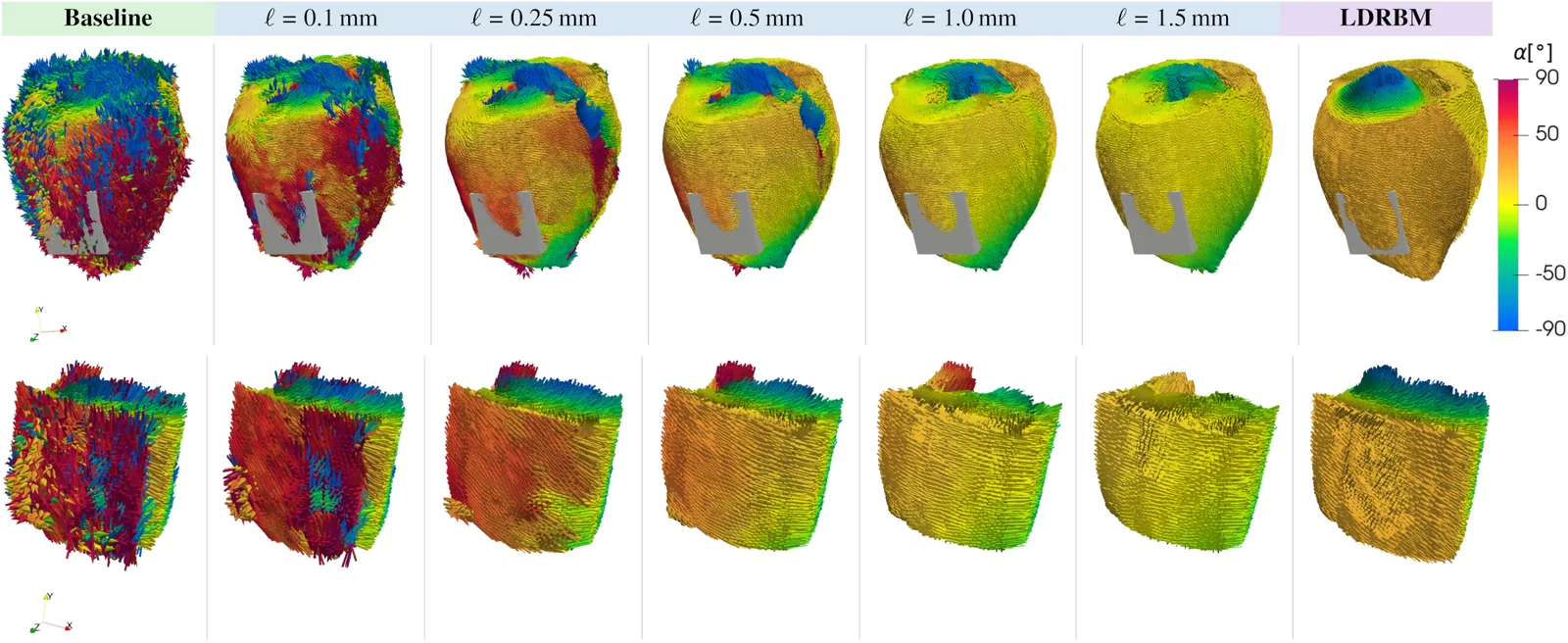
Comparison different fiber fields derived by varying the regularization radius ℓ with respect to the the unfiltered fiber field and the LDRBM one. On the left the unfiltered fiber field and on the right the LDRBM one [52]. The first row shows the full geometry. The second row shows a magnified view of the region highlighted by the gray boxes in the first row, with an edge length of 3.0 mm. The colormap represents the angular variation of α within the myocardium.

Fig 3 compares the baseline fiber field obtained from experimental measurements with the corresponding regularized fields for increasing values of the regularization radius ℓ, as well as with the fiber field generated by the LDRBM. In the present work, the maximum diameter of the base is of approximately 8 mm.

### 2.4 Prescribing the fiber orientations via LDRBM

The Doste et al. RBM [50,52] surrogates the fiber architecture by interpolating specific angular values of α and γ at the epicardium and endocardium and linearly interpolating them along the transmural direction. A convex combination is performed depending on ϕ as follows


$$\begin{array}{c} \alpha_{RBM}=\left\{ \begin{array}{cc} -\;\alpha_{Endo,RV}\;\phi +\alpha_{Epi,RV}\;(\phi -1), & \text{in }\Omega_{RV},\\ {}[6pt]\alpha_{Endo,LV}\;\phi +\frac{\alpha_{Epi,LV}}{2}\;(1-\phi ), & \text{in }\Omega_{LV}, \end{array}\right. \end{array}$$ (18)

where $\alpha_{endo,RV}$, $\alpha_{endo,LV}$, $\alpha_{epi,LV}$ and $\alpha_{epi,RV}$ are the prescribed angles at right and left endocarium and epicardium, respectively, while $\Omega_{LV}$ and $\Omega_{RV}$ denote the left and right ventricular domains. This procedure is applied to both the α and γ angles. The resulting angles $\alpha_{RBM}$ and $\gamma_{RBM}$ are then used in Eq. (9) to construct the myocardial reference system.

To identify $\alpha_{Endo,RV}$, $\alpha_{Endo,LV}$, $\alpha_{Epi,RV}$, $\alpha_{Epi,LV}$, $\gamma_{Endo,RV}$, $\gamma_{Endo,LV}$, $\gamma_{Epi,RV}$ and $\gamma_{Epi,LV}$, since the helix-angle distribution varies significantly accross species [80], we do not use literature values, but rather we compute the statistical distribution of α and γ selecting the modal value in different myocardial sub-regions, as done in [23] (see Fig 5). We reported the computed mode values in S1 Appendix.

**Fig 4:**
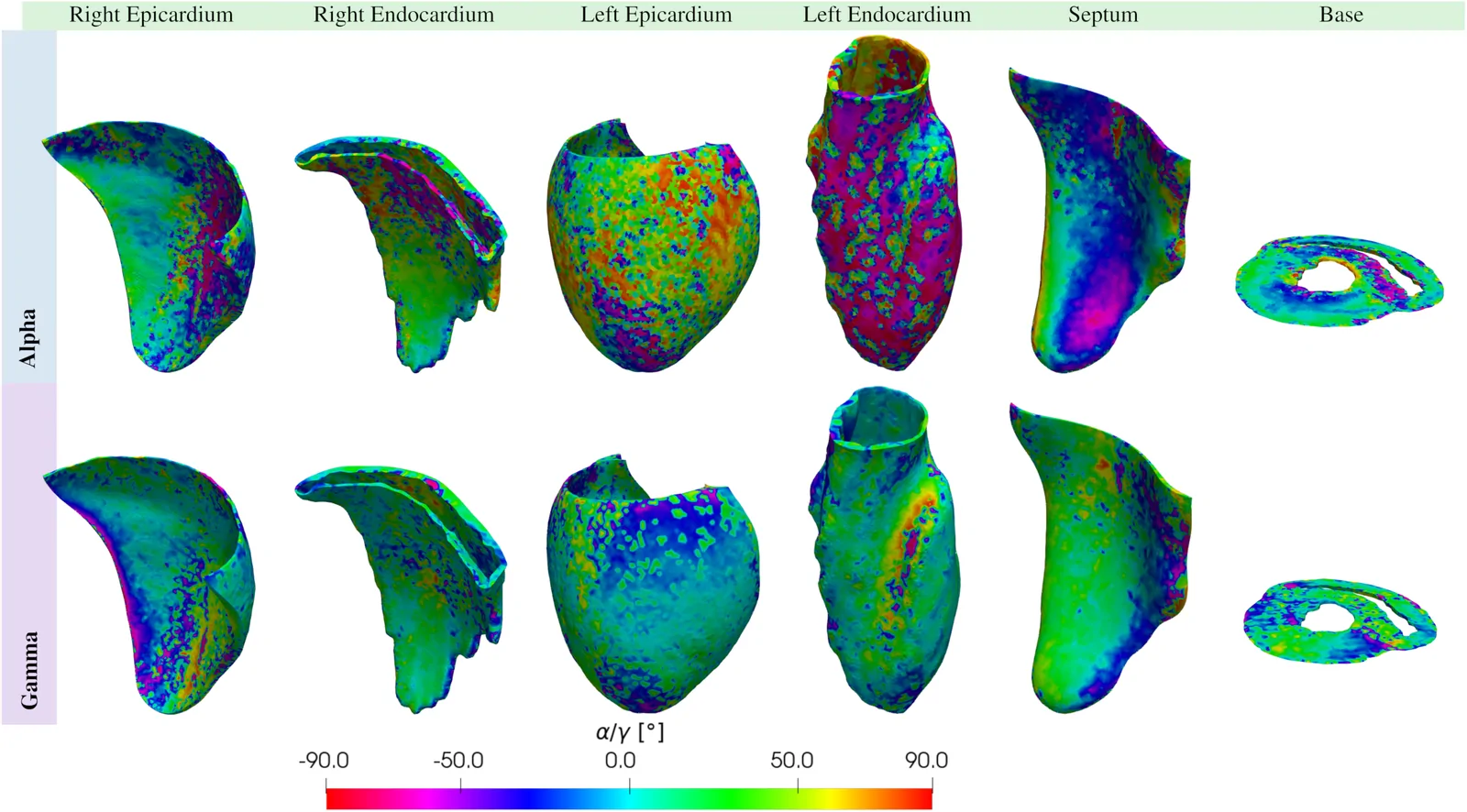
Angles mapping. Variation of α and γ angles within the ventricular sub-regions.

**Fig 5:**
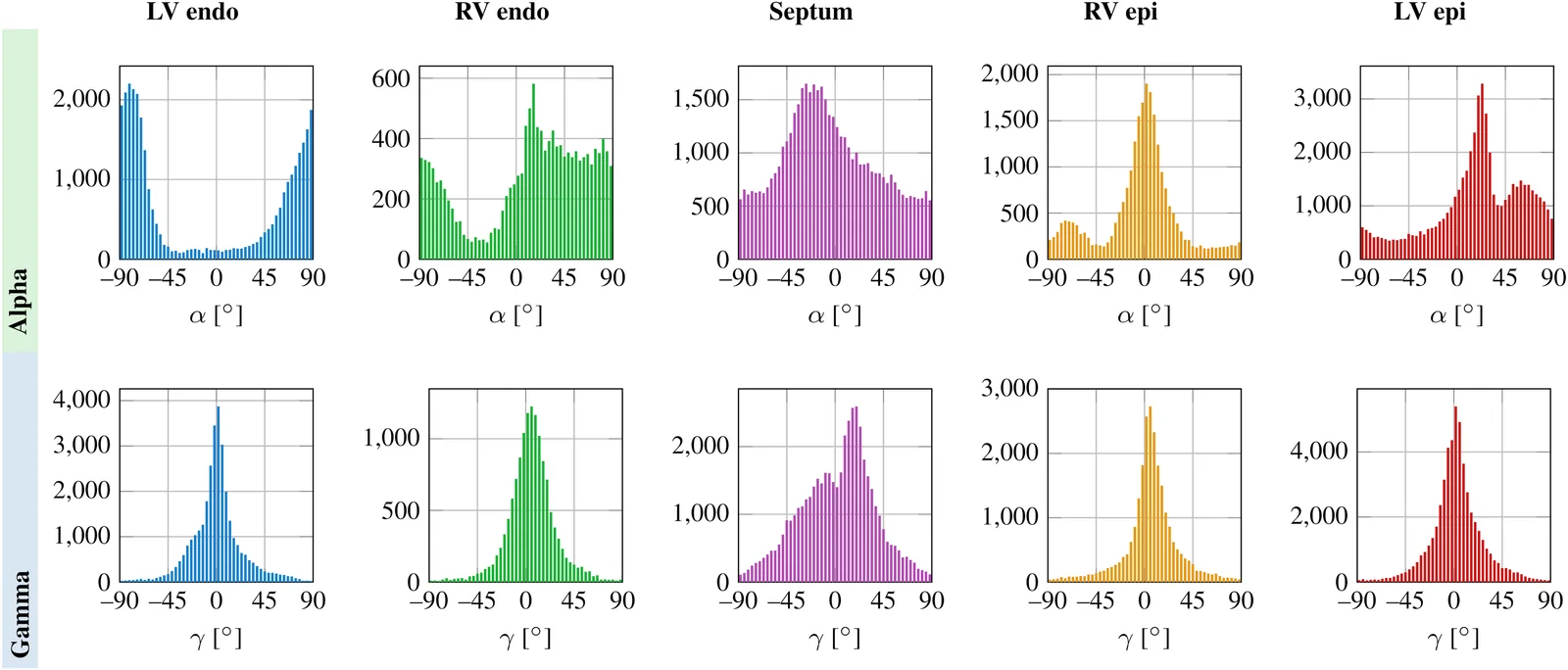
Angles distribution. Distribution of the α and γ angles in different zones of the domain.

Fig 4 and 5 report the values of the angles α and γ across different regions of the ventricular domain. Compared with α, γ is relatively uniform and remains near zero. This suggests that global ventricular contraction is primarily driven by rotations in the ${𝐞}_{l}$–${𝐞}_{n}$ plane, which exhibit a pronounced transmural variation from endocardium to epicardium. In particular, the fiber orientation within the septum (both in the portions associated with the LV and in those associated with the RV) differs substantially from that observed in the endo-epicardial regions of the LV/RV free walls, in agreement with [81]. In the lateral wall, fiber orientation rotates smoothly from the endocardial to the epicardial layers. In contrast, within the septal wall, an inverse rotation from the antero-posterior direction to the inferior-superior direction is observed. Moreover, α displays significant variability even within the same region, suggesting that assigning a single fiber angle value within the entire epicardium and endocardium may not be sufficient to accurately represent local myocardial fiber orientation. Finally, it is worth noting that the LDRBM by Doste et al. [52] does not provide a reconstruction of the β angle, assuming a transversely isotropic material behavior.

### 2.5 Electromechanical model

The electromechanical model consists of multiple core modules, each representing different physical processes: electrophysiology, activation, mechanics, and blood circulation. The electrophysiology component determines the activation time, defined as the instant at which the electrical stimulus reaches each point in the cardiac domain, as well as the calcium ion concentration. These quantities are then used as input to the activation core model, which computes the active tension along the fibers by accounting for both calcium concentration and local deformations. The mechanics combines the contributions of active tension and passive tissue behavior to compute displacements, while the blood circulation component, which represents the hemodynamics of the entire cardiovascular system, provides the pressure boundary conditions at the endocardium [68,71,82].

Under the physiological conditions considered, the electrophysiology component is described using an eikonal-diffusion formulation [83]. This enables the efficient computation of activation times by solving an eikonal equation. The local calcium concentration is computed by evaluating an experimentally derived calcium transient at the activation time associated with each point, following the approach proposed in [84]. The electrical signal propagation across the myocardial domain is governed by the conductivity tensor 𝐃, defined as


$$\begin{array}{c} 𝐃=\sigma_{f}\frac{𝐅{𝐟}_{0}⊗𝐅{𝐟}_{0}}{{\left|\left|𝐅{𝐟}_{0}\right|\right|}^{2}}+\sigma_{s}\frac{𝐅{𝐬}_{0}⊗𝐅{𝐬}_{0}}{{\left|\left|𝐅{𝐬}_{0}\right|\right|}^{2}}+\sigma_{n}\frac{𝐅{𝐧}_{0}⊗𝐅{𝐧}_{0}}{{\left|\left|𝐅{𝐧}_{0}\right|\right|}^{2}}, \end{array}$$ (19)

where $\sigma_{f}$, $\sigma_{s}$, and $\sigma_{n}$ are the conductivities in the fiber, sheet, and normal directions, respectively. In modeling practice, $\sigma_{f}$ is typically assumed to be twice as large as $\sigma_{S}$, with $\sigma_{S}$ set equal to $\sigma_{N}$. Hence, the fiber orientation ${𝐟}_{0}$ establishes a preferential direction for signal propagation.

The cardiac mechanics, describing the dynamics of the tissue displacement 𝐝, is modeled using the momentum conservation equation under the hyperelasticity assumption, combined with an active stress approach [31,71]. The deformation gradient tensor, computed from the displacement field 𝐝(𝐱), is defined as 𝐅=𝐈+∇𝐝(𝐱).

The constitutive behavior of the myocardium is governed by the Piola-Kirchhoff stress tensor, defined as


$$\begin{array}{c} 𝐏=\frac{\partial 𝒲(𝐅)}{\partial 𝐅}+m_{f}T_{a}\frac{𝐅{𝐟}_{0}⊗{𝐟}_{0}}{\left||𝐅{𝐟}_{0}|\right|}+m_{s}T_{a}\frac{𝐅{𝐬}_{0}⊗{𝐬}_{0}}{\left||𝐅{𝐬}_{0}|\right|}+m_{n}T_{a}\frac{𝐅{𝐧}_{0}⊗{𝐧}_{0}}{\left||𝐅{𝐧}_{0}|\right|}, \end{array}$$ (20)

where 𝒲 is the Usyk strain potential energy established with transversely isotropic parameters [67]; $T_{a}$ is the active tension, and $m_{f}$, $m_{s}$, and $m_{n}$ are the Stress Factors (SF) in the fiber, sheet, and normal directions, respectively [68]. The SF configuration $m_{f}=1$, $m_{s}=m_{n}=0$ corresponds to an active contraction occurring solely along the fiber longitudinal direction. In computational modeling practice, the other components can be activated to surrogate the effect of fiber disarray [68,69].

The activation provided by the RDQ20-MF model proposed by [85]. The latter computes the active tension field by solving a system of 20 ordinary differential equations. The RDQ20-MF model offers accurate results under physiological conditions at low computational cost, while accurately capturing cooperative, length-dependent activation and force-velocity relationships [71,85]. The output of the RDQ20-MF model is the active tension $T_{a}$, defined as


$$\begin{array}{c} T_{a}=a_{XB}\;𝒢([{Ca}^{2+}],SL), \end{array}$$ (21)

where $a_{XB}$ is the crossbridge stiffness, which linearly scales the active tension with respect to the crossbridge contraction in the sarcomere, 𝒢; $[{Ca}^{2+}{]}_{i}$ is the calcium ion concentration provided by the electrophysiology model; and SL is the sarcomere length, resulting from fiber contraction computed in the mechanics. The crossbridge stiffness $a_{XB}$ is set to 23 MPa baseline simulation. This parameter can be adjusted to reduce or increase the global contractility of the myocardium. Furthermore, the $a_{XB}$ in the RV is half of that in the LV, effectively imposing a contractility ratio of 1/2 between LV and RV.

Mechanical boundary conditions are imposed in four cardiac zones: epicardium, left and right endocardium, and the ventricular base. Robin-type boundary conditions are applied at the epicardium and basal plane, following the approach in [86]. These boundary conditions introduce stiffness and damping parameters that reproduce the interaction between the myocardium and the pericardium. This interaction is modeled as springs and dashpots acting in the normal and tangential directions with respect to the epicardium. At the base, less stiff Robin boundary conditions are applied to reproduce the effect of the atria on the ventricles. At the endocardium, the mechanical model is coupled with the blood circulation model through Neumann boundary conditions accounting for the pressure exerted by the blood inside the chamber.

The blood circulation of the entire cardiovascular system is modeled using a 0D closed-loop approach, as proposed in [82]. The systemic and pulmonary circulations are represented by 0D Resistance–Inductance–Capacitance (RLC) networks, in which blood flow rate is analogous to electrical current, and pressure corresponds to electrical potential. Heart chambers are modeled as time-varying elastance elements, while non-ideal diodes represent the heart valves [68,71,82].

#### 2.5.1 Strain computation.

To analyze the spatial distribution of deformation, we compute the first three invariants of the Green–Lagrange strain tensor 𝐄(x)


$$\begin{array}{c} 𝐄(x)=\frac{1}{2}({𝐅}^{T}(𝐱)𝐅(𝐱)-𝐈), \end{array}$$ (22)

where 𝐅(𝐱)=𝐈+∇𝐝(𝐱) is the deformation gradient and 𝐈 is the Identity tensor. The first invariant *I*_1_, given by the trace of the tensor 𝐄, ${𝐈}_{1}=\mathrm{tr}(𝐄)$, provides a measure of the overall level of strain and is commonly associated with volumetric dilatation in the small-to-moderate deformation regime. The second invariant $I_{2}=\frac{1}{2}[(\mathrm{tr}(𝐄{)}^{2})-\mathrm{tr}({𝐄}^{2})]$ captures distortional deformation by describing the deviatoric part of the strain tensor, thereby quantifying shape changes independently of volume variations. The third invariant *I*_3_, given by the determinant of the Green–Lagrange strain tensor $I_{3}=\det({𝐅}^{T}𝐅)$ characterizes higher-order nonlinear strain effects, and is associated with changes in volume.

#### 2.5.2 Numerical Implementation.

The numerical framework has been implemented within life${\;}^{\text{x}}\}$ (https://lifex.gitlab.io) [87–90], an in-house high-performance C++ Finite Elements (FE) library focused on cardiac applications based on deal.II FE core (https://www.dealii.org) [91,92]. The simulations were run on one CPU node powered by two 24-core CPUs with 512 GB RAM and required approximately 2000 s to reproduce one heartbeat.

The multiphysics problem is discretized on a tetrahedral mesh composed of 328 000 nodes, with an averege edge length of 150 μm and a minimum cell diameter of 91 μm. We selected the minimum mesh size to capture the fiber field variations without unnecessarily increasing the computational cost. Space discretization is based on linear FE, and the time discretization is performed using a Backward Difference Formula (BDF) of second order for the acceleration term. We solve the non-linear equations at each timestep using the Newton method. Finally, for the blood circulation, a forward Euler scheme is employed. The circulation model is then coupled with the mechanics through a Lagrange multiplier formulation, in which the pressures of the LV and RV act as Lagrange multipliers in the coupled circulation-mechanics problem [68,71,82].

The timestep for electromechanics and blood circulation is set to 1 ms. Each heartbeat for the physiological mouse heart lasts 200 ms, and 5 heartbeats are simulated. The ectrophysiological parameters were calibrated using a heart-specific activation map obtained from experimental measurements, and the activation model was tuned based on shortening twitch test results. To calibrate the remaining parameters, we relied on physiological data of blood circulation Quantity of Interest (QoI) derived from the literature [93,94]. Furthermore, to reduce the computational time and resources required during the tuning phase, we employed the 0D emulator proposed by [95]. This emulator allows the computation of convenient initial conditions for the 3D simulation, enabling faster convergence toward the limit cycle [68]. It can also be used to calibrate the parameters of the RLC circuit representing blood circulation, as well as selected parameters of the 3D model. Further details are provided in S1 Appendix.

## 3 Results

In this section, we report the numerical results obtained with the cardiac electromechanical model, focusing on the role of the fiber field and the associated fiber disarray on the electrophysiology (Sec. 3.1), passive mechanics (Sec. 3.2), and finally on the overall electromechanical function (3.3). In this analysis, the baseline simulation, built on the experimental fiber field, is taken as reference result and simulations built on regularized or LDRBM fiber fields are compared with it.

In the murine heart, global mechanical function is predominantly governed by the LV, whereas the RV operates under substantially lower pressure and mechanical load due to the low-resistance pulmonary circulation [96]. Moreover, owing to its extremely thin free wall and strong ventricular interdependence, RV dynamics are largely driven by LV contraction. Accordingly, in the following electromechanical analysis we primarily focus on the LV.

### 3.1 Electrophysiology

We evaluated the influence of the fiber field on the propagation of the electrophysiology signal through the myocardium. Tissue conductivity was modeled as transversely isotropic, with fiber-longitudinal conductivity twice that of the sheet and normal directions.

**Fig 6:**
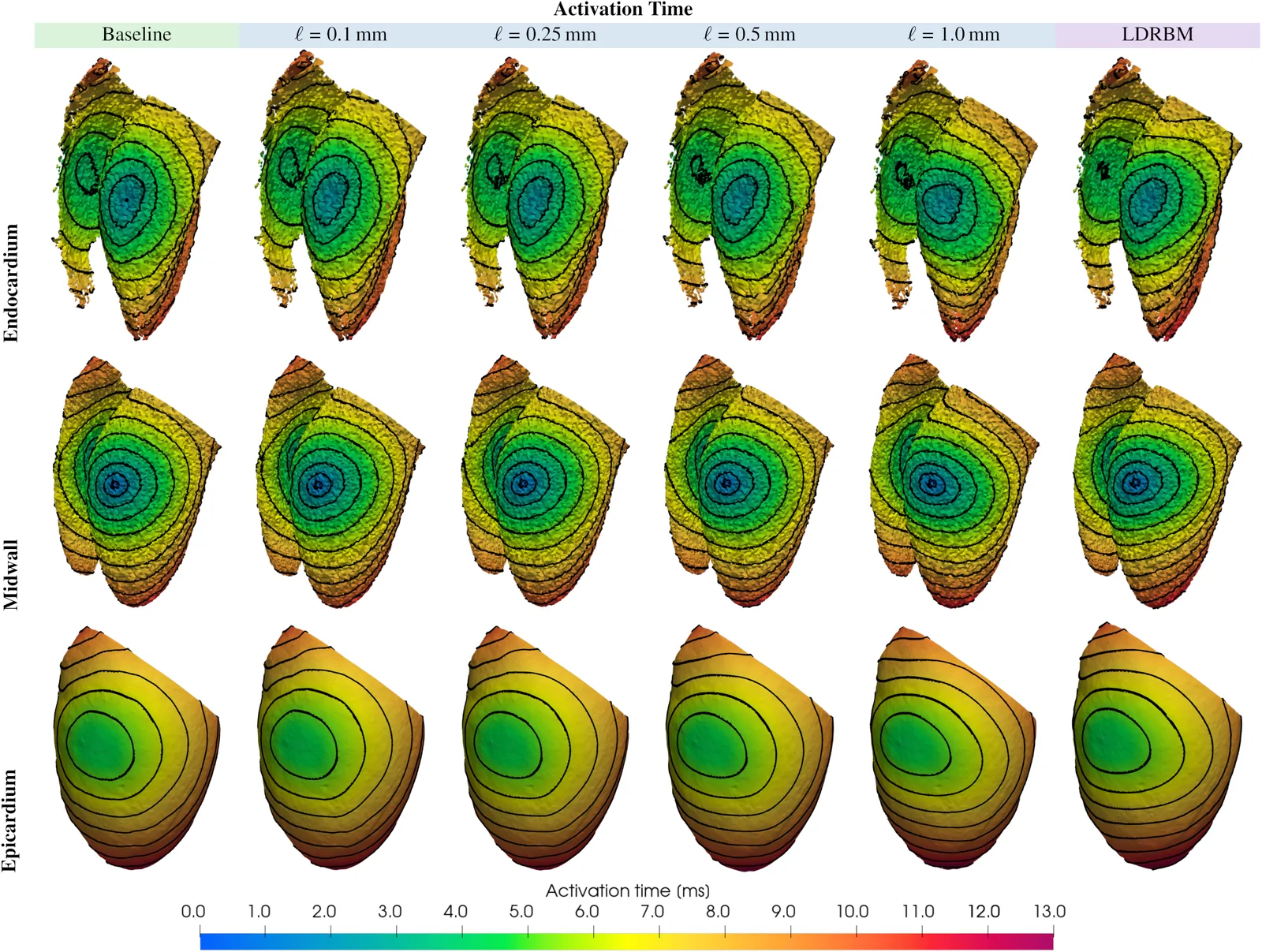
Influence of the fiber field on electrophysiology propagation in the myocardium. The distance between the isochrones is 1.0 ms, while the spatial scale is represented by the cube of edge length equal to 1.5 mm in the top left image. Endocardial and epicardial activation maps are shown for different fiber architectures: experimental (baseline), varying regularization radii ℓ, and the LDRBM fibers. Furthermore the total activation time *T*_max_ is reported for every fiber field.

Fig 6 shows the endocardial and epicardial activation maps together with the total activation time *T*_max_, for increasing fiber regularization radius and for the LDRBM. The results for the baseline case are aligned with the literature [97]. Wavefronts originating from the stimulation sites propagate preferentially along the higher-conductivity fiber direction. As expected, in the experimental fiber field, the presence of disarray reduced the directional effect of faster conduction along the fibers, resulting in a more isotropic propagation pattern compared to LDRBM fibers. However filtering out the disarray results only in slight reduction of *T*_max_ for ℓ≤0.25 mm. Moreover, as the fiber regularization radius increases, the preferred direction of propagation in the longitudinal direction becomes more pronounced. Overall, the LDRBM produces an activation pattern similar to that of the regularized fiber field and its *T*_max_ is in between the total activation time of the case with ℓ=0.5 mm and the case with ℓ=1.0 mm, suggesting that LDRBM captures the mean fiber orientation. However, signal propagation with LDRBM is more anisotropic compared to the experimental fiber field. *T*_max_ shows therefore a weak dependence on the regularization radius as it varies only between 11.67 ms and 11.98 ms when ℓ≤0.5 mm. To observe a meaningful change in *T*_max_, the regularization must be increased to 1.0 mm.

### 3.2 Passive mechanics

We investigated how the fiber architecture affects passive inflation of the biventricular geometry. The following conditions were considered: no active stress, prescribed cavity pressures without explicitly modeling the blood circulation. The resulting numerical problem was solved by Newton-Raphson iterations using pressure as the control variable. Pressure was progressively applied to both the LV and RV, increasing from 0 to 37.5 mmHg in 50 equal increments. This approach allowed us to examine the cardiac passive response across a range that includes physiological diastolic pressures.

**Fig 7:**
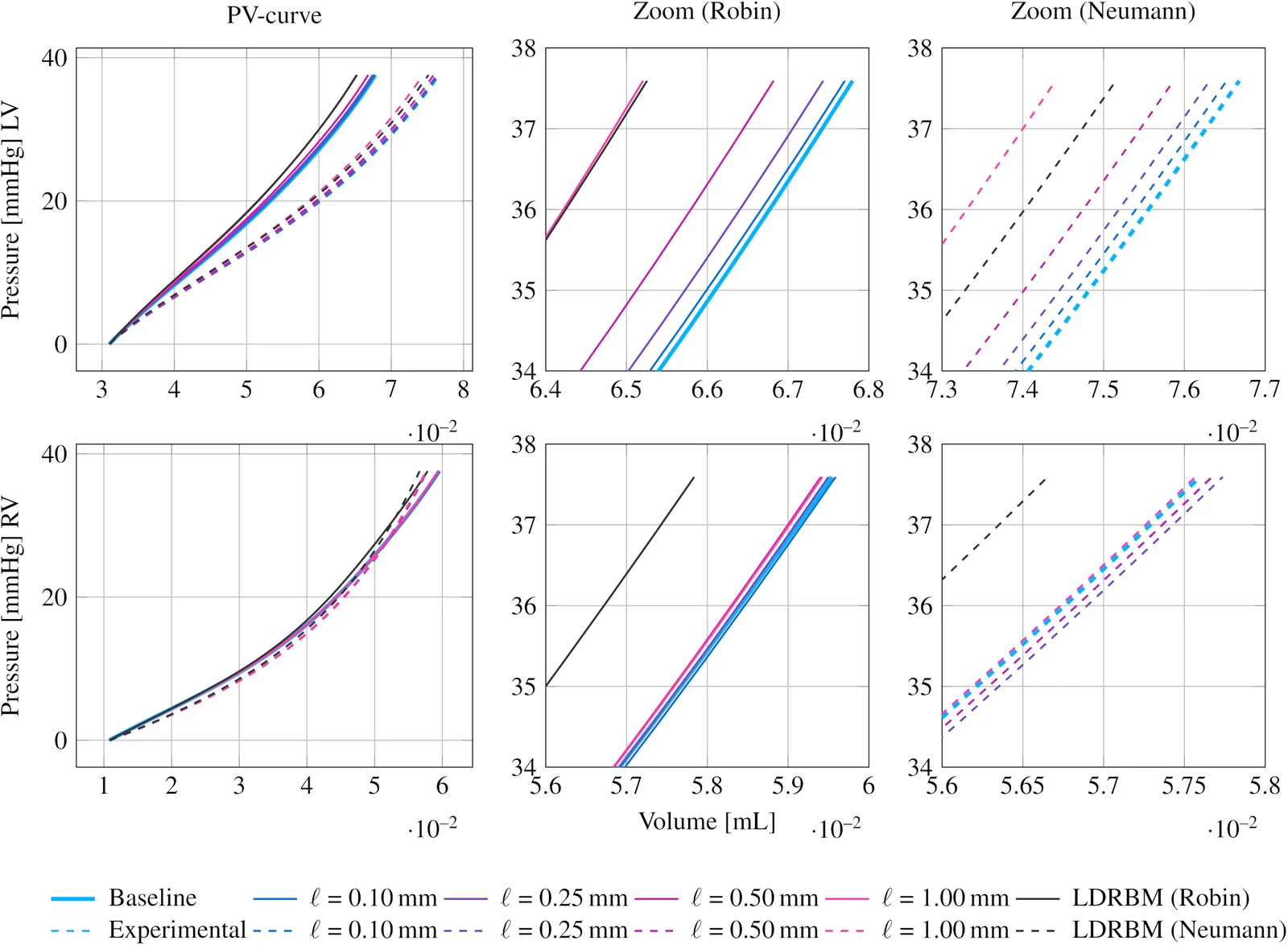
Passive inflation of the left (PV-curve LV) and right (PV-curve RV) ventricles under increasing pressure, in absence of active contraction. Comparison among different fiber architectures: experimental (baseline), progressively smoothed fibers with increasing regularization radius ℓ, and LDRBM. Two cases are shown. The first one (solid lines) correspond to the baseline BCs with Robin BCs at the base and at the epicardium. The second case (dashed lines) corresponds to homogeneous Dirichlet BCs at the base and Neumann BCs at the epicardium. The second column shows the zoomed views of the Robin BCs case, the third column shows the zoomed views of the Neumann BCs case.

Fig 7 compares the inflation curves of LV and RV obtained with different fiber configurations: experimental, progressively smoothed fields (with increasing regularization radius, as described in 2.3, see also Fig 3), and Doste el al. LDRBM [52]. Moreover, we analyzed two different cases. In the first case, we used Robin BCs at the base and at the epicardium, as in the baseline simulation (see S1 Table); in the second case we replaced the Robin BCs at the epicardium with stress-free (Neumann) BCs, and applied homogeneous Dirichlet BCs at the base to constraint the motion. The rationale behind the letter case was to reduce the effect of BCs on the elastances of the chambers, thus isolating the effect of the fiber field. The results show that, in both the cases considered, reduced fiber dispersion (i.e., greater alignment coherence) slightly increases myocardial stiffness. More precisely, increasing ℓ from 0 mm to ℓ=1.0 mm reduces LV volume variation at P=37.5 MPa by 4% with Robin BCs and by 3% with Neumann BCs. Moreover, replacing the experimental fiber field with the LDRBM one yields a similar variations. Since these trends are consistent across boundary-condition setups, we conclude that the observed differences can be attributed primarily to fiber architecture and disarray, rather than to the influence of the surrounding tissues.

### 3.3 Electromechanics

We analyzed the effects of the fiber architecture on the overall electromechanical behavior. We stress that when electromechanical function is considered, the fiber architecture influences all the major components of the heart model. Specifically, fiber orientation affects passive mechanics and electrophysiology, as previously discussed (in Sec. 3.1 and 3.2), and also plays a pivotal role in the active stress (see Sec. 2.5 and Eq. (20)).

To assess the impact of fibers on the electromechanical function, we employ two types of readout: pressure-volume data – providing global indication of the pumping function of the heart – and strain maps – yielding local information on the tissue mechanical response.

#### 3.3.1 Pressure-volume analysis.

**Fig 8:**
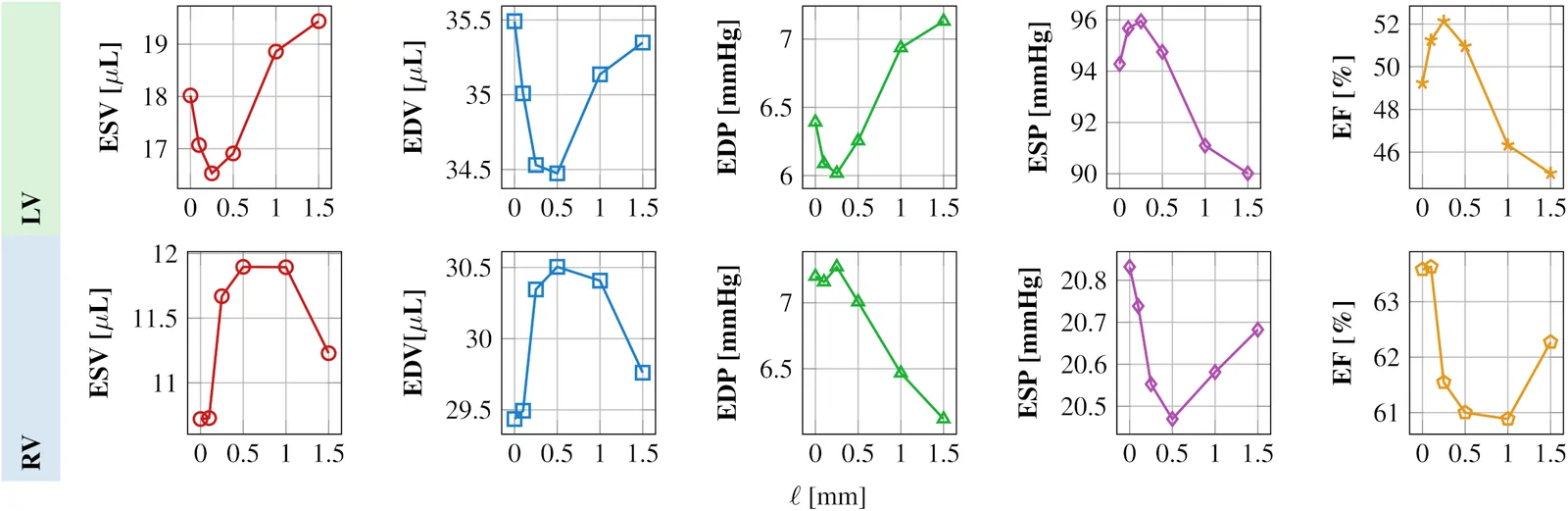
QoI at varying regularization radius. Pressures and volumes of LV and RV during systole and diastole, and corresponding ejection fractions, obtained for different values of the regularization radius ℓ.

Fig 8 shows the variation of several QoI with respect to the fiber regularization radius ℓ: the End Diastolic Volume (EDV) and Pressure (EDP), the End Systolic Volume (ESV) and Pressure (ESP) and the Ejection Fraction (EF), which provides a measure of ventricular efficiency. Fig 8 shows a maximum EF of LV at ℓ=0.25 mm and reveals a non-monotonic effect of fiber regularization on cardiac contractility. In addition, for LV, the increases in EDP and ESP peaks at ℓ=0.25 mm indicate an increase in the amount of energy transferred to the blood. This dual-phase behavior can be interpreted as the result of two distinct regimes induced by the fiber regularization radius ℓ. For small values of ℓ, increasing ℓ primarily reduces the local fiber disarray while preserving the underlying macroscopic fiber architecture. Moreover, LV contraction becomes more effective, as myofibers act in a more coordinated manner, with less active mechanical energy dissipated along misaligned fibers. Conversely, when the regularization radius ℓ approaches the characteristic length scale of the macroscopic fiber architecture, further smoothing progressively degrades the typical helical fiber organization, which is specifically structured to ensure a mechanically efficient contraction. As a result, the effectiveness of LV contraction decreases. In addition EDV does not remain constant, when varying ℓ, but it follows the same trend of the ESV, suggesting the effect of a residual tension that increases when ℓ≈0.25 mm. The residual tension limits the full expansion of the LV at end diastole.

Based on the above observations, we interpret the fiber field obtained for ℓ=0.25 mm as a reliable representation of the effective macroscopic fiber architecture, while the discrepancy between this field and the originally measured one can be attributed to fiber disarray. An opposite behavior with respect to LV can be observed for RV. The latter reflects the strong ventricular interdependence, with RV dynamics predominantly dictated by LV activity [96].

**Fig 9:**
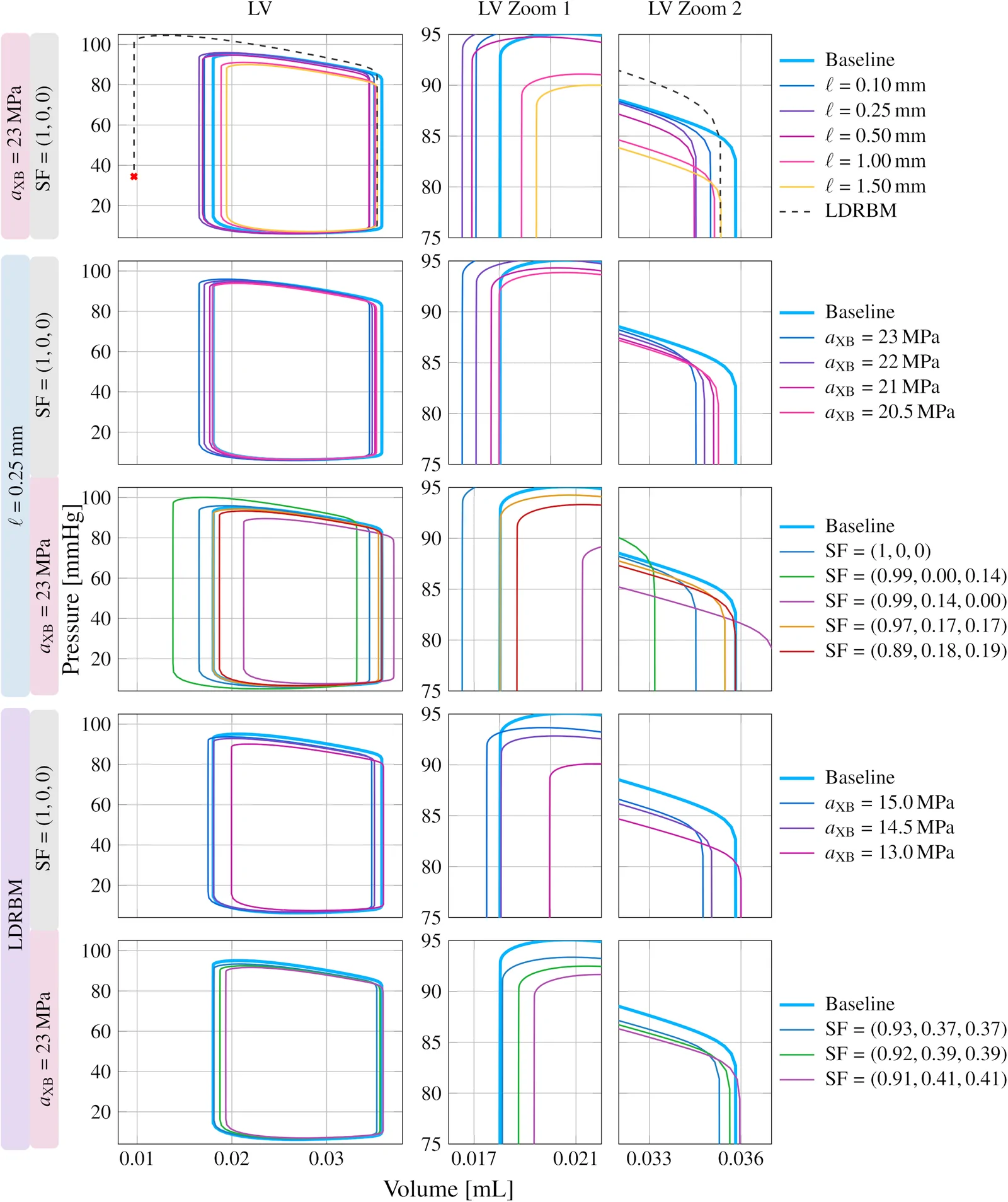
LV PV loops comparison. PV loops of LV (first column) with zooms in end-systolic (LV Zoom 1, second column) and end-diastolic (LV Zoom 2, third column) zooms. The first row shows PV loops resulting from the baseline simulation (i.e., with the experimental fibers) compared with regularized and LDRBM fibers. Rows two and three illustrate PV loops using the regularized fiber field, while rows four and five show PV loops with the LDRBM fibers. The calibration was performed by adjusting $a_{XB}$ (rows two and four) and SF (rows three and five).

The first row of Fig 9 compares the LV PV-loop from the baseline simulation (using experimental fibers) with PV-loops obtained using regularized and LDRBM fiber fields. For ℓ≤0.25 mm, fiber regularization induces a leftward shift of the LV PV loop. Further increasing the regularization radius ℓ causes the LV PV loop to shift rightward, resulting in a decrease in EF and, consequently, in pumping efficiency. This behavior can be explained by the reduction of local fiber disarray when the regularization radius remains much smaller than the myocardial wall thickness, facilitating more a coordinated muscle contraction. Conversely, larger regularization radii ℓ progressively disrupt the original helical fiber architecture responsible for an optimal ventricular contraction. Fig 9 also reports the PV loop obtained using LDRBM fibers. In this case, the simulation failed during systole due to excessively high contraction levels, which caused the mesh element collapse. This behavior is attributed to the idealized nature of the rule-based fiber architecture, which enforces a perfectly helical fiber arrangement.

#### 3.3.2 Accounting for the presence of disarray in computational models.

Active force is largely directed along longitudinal directions, so fiber disarray can have a significant impact on both the magnitude and distribution of active tension. We remark that most computational models account for this effect by introducing active stress components along directions orthogonal to the fibers [69,76] (see Sec. 2.5). In Eq. (20), this effect is represented by assigning nonzero values to $m_{s}$ and/or $m_{n}$. Commonly, however, these components are set to zero ($m_{s}=m_{n}=0$), and the effect of disarray is implicitly included by calibrating the fiber contractility parameter [66,98,99].

In this study, the availability of a high-resolution experimentally measured fiber field, together with a modeling framework in which fiber disarray is progressively removed through smoothing, provides a unique opportunity to assess the impact of fiber disarray on active mechanics and to evaluate modeling strategies designed to account for it. Moreover, this framework enables us to assess whether the smoothed fiber field produces responses comparable to those observed with the experimental disordered architecture.

For the purposes of this analysis, and for the reasons outlined in Sec. 3.3.1, we use the smoothed fiber field obtained with ℓ=0.25 mm as representative of the macroscopic fiber architecture. We then investigate whether the proposed surrogate strategies for fiber disarray (i.e., variations of the SF parameters, see Eq. (20)) are able to reproduce the functional consequences of the experimentally observed disordered architecture.

We first investigate the impact of variations in the contractility parameter $a_{XB}$. As shown in the second row of Fig 9, decreasing $a_{XB}$ causes a rightward shift of the PV loops for the regularized fiber field (ℓ=0.25 mm). Overall, adjusting contractility alone cannot fully reproduce the baseline LV response, but reducing $a_{XB}$ from 23 MPa to $a_{XB}=20\;MPa$ (–13%) provides a good approximation of the Left Ventricle (LV) behavior, see second row of Fig 9.

We then evaluate the effect of introducing active stress components along the cross-fiber directions ${𝐬}_{0}$ and/or ${𝐧}_{0}$. To isolate directional effects while preserving the overall level of activation, we enforce the constraint $m_{f}^{2}+m_{s}^{2}+m_{n}^{2}=1$. The third row of Fig 9 illustrates that the sheet and normal components of active tension $T_{a}$ produce markedly different responses. Adding normal-direction activation causes a leftward shift of the PV loop, while sheet-direction activation shifts it rightward. The latter effect is evident when comparing the fiber-normal activation case SF=(0.99,0.00,0.14) with the fiber-sheet activation SF=(0.99,0.14,0.00). When both the cross-fiber directions are activated, SF=(0.97,0.17,0.17), the resulting PV loop closely matches the baseline simulation (i.e., with the experimental fibers).

Finally, we consider a configuration in which active force is applied along the three directions ${𝐟}_{0}$, ${𝐬}_{0}$, and ${𝐧}_{0}$, weighted according to the average projection of the experimentally measured fiber directions onto these orthogonal axes. Specifically, we compute the quantities $\left|{𝐟}^{meas}\cdot {𝐟}_{0}\right|$, $\left|{𝐟}^{meas}\cdot {𝐬}_{0}\right|$, $\left|{𝐟}^{meas}\cdot {𝐧}_{0}\right|$, where ${𝐟}^{meas}$ is the experimentally measured fiber direction, and then average them over the entire myocardium. The resulting values correspond to the coefficients SF=(0.89,0.18,0.19). The use of absolute values reflects the directional invariance of the applied active force. It is worth noting that $\sqrt{m_{f}^{2}+m_{s}^{2}+m_{n}^{2}}=0.93<1$, so that this approach effectively combines a reduction of the effective contractility with the introduction of active force components in the cross-fiber directions. The resulting PV loop closely approximates the baseline simulation, exhibiting a better fit during diastole compared to the case SF=(0.92,0.39,0.39), albeit with reduced agreement during systole.

The approaches previously applied to the experimental fiber field (with ℓ=0.25 mm) are next tested using the LDRBM fibers. In this case, good approximations for the LV PV-loop are obtained by reducing the crossbridge stiffness $a_{XB}$, as shown in the fourth row of Fig 9, and also by introducing the misalignment for $T_{a}$, as illustrated in fifth row of Fig 9. The closest agreement is obtained for $a_{XB}=12.5\;MPa$ and for SF=(0.92,0.39,0.39). Nonetheless, the recovery of the baseline PV loop exhibits a larger approximation error relative to the regularized fiber field. This suggests that the average fiber orientation has a greater impact on contraction than fiber disarray. As a result, replacing disarray with SF does not fully capture the baseline contraction.

#### 3.3.3 Strain field analysis.

**Fig 10:**
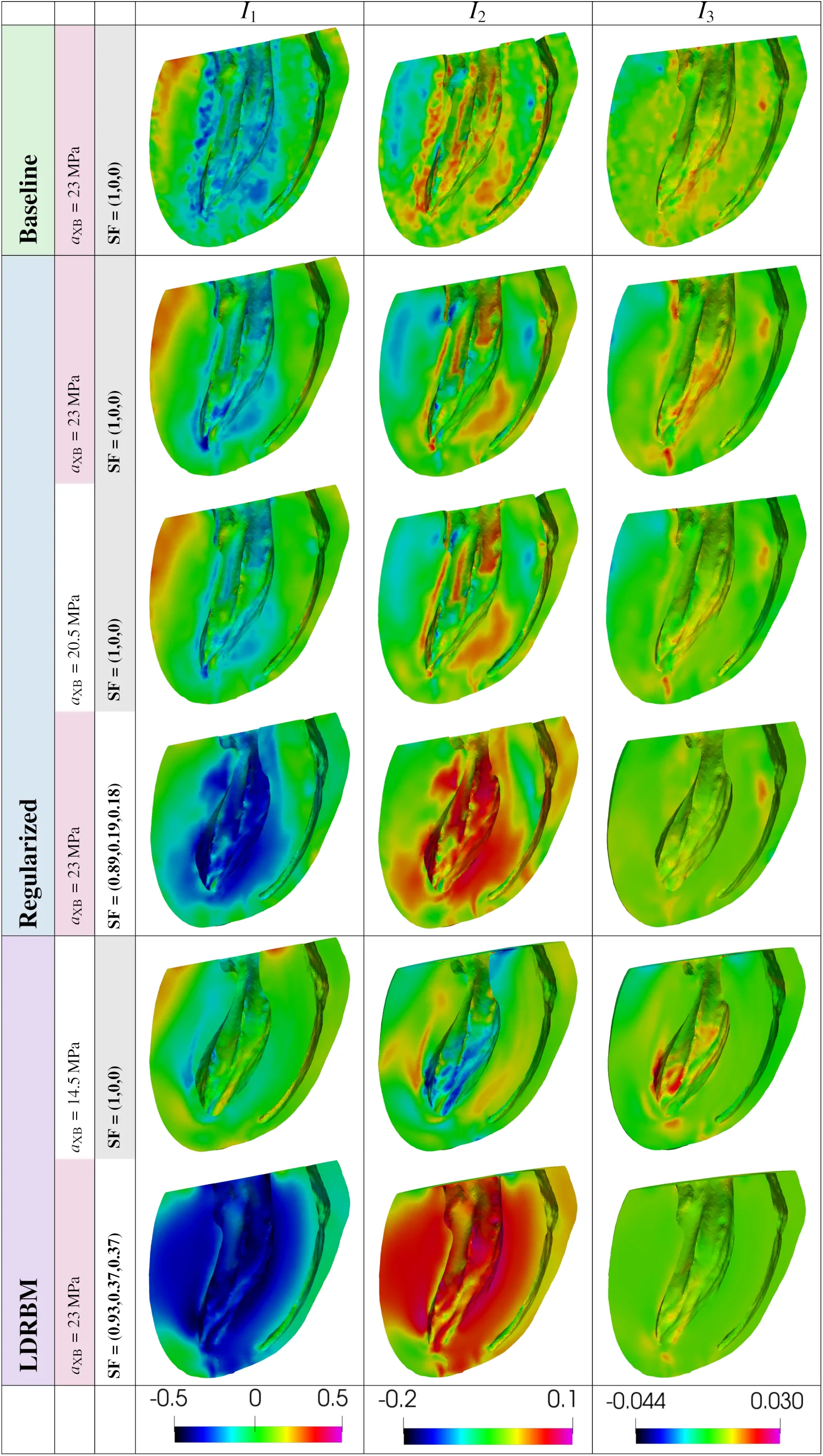
Invariants analysis. Comparison of the first three invariants *I*_1_, *I*_2_, *I*_3_ of the Green-Lagrange strain tensor *E* (see Eq. (22)) obtained from different fiber fields: experimental (baseline), regularized fibers and LDRBM.

Fig 10 compares the strain fields obtained for the baseline simulation, the regularized fiber field (ℓ=0.25 mm), and the LDRBM-based simulation. For each fiber architecture, we also include the configurations that best reproduce the PV loop of the baseline simulation. For the regularized fiber field, these correspond to the cases with $a_{XB}=20.5\;MPa$ and SF=(0.89,0.19,0.18), whereas for the rule-based fibers, the selected configurations use $a_{XB}=12.5\;MPa$ and SF=(0.92,0.39,0.39).

As shown in the second row of Fig 10, the regularized fiber field leads to a more diffused distribution of strain. While the main patterns of dilatation and distortion are preserved (with respect to the baseline), high-frequency spatial variations linked to fiber disarray are reduced.

A comparable strain pattern is observed in the reduced $a_{XB}$ (see Fig 10, third row), with a reduction in the overall magnitude of both dilatation and distortional components. Conversely, the case with cross-fibers SF activation (see Fig 10, fourth row) exhibits marked discrepancies with respect to the baseline simulation. Although strain concentration at the left endocardium is captured, local variations are largely not resolved. In particular, dilatation at the left epicardium is absent, while spurious distortion appears in the right myocardium. This behavior can be attributed to the use of globally averaged SF parameters, which act uniformly throughout the domain, whereas the experimentally fiber disarray shows significant heterogeneity. As a result, the SF surrogate approach tends to enhance contraction uniformly, increasing contraction at the endocardium while suppressing dilatation at the epicardium.

In the LDRBM fiber simulation, with reduced $a_{XB}$, the first invariant *I*_1_ shows a contraction pattern similar to the experimental fiber cases, albeit with lower agreement than in the regularized fiber field. This suggests that the macroscopic fiber orientation is only globally captured by the rule-based approach. On the other hand, shear distortion is localized at the midwall, localized by the linear transmural variation of fiber orientation, and at the left endocardium, where the sign of shear differs from the baseline. Finally, the LDRBM case with cross-fibers SF activation yields strain fields that are markedly different from the baseline, with a predominantly negative first invariant and a uniformly positive second invariant, thereby failing to reproduce the localized strain patterns observed in the baseline simulation.

## 4 Discussion

In this work, we developed a biventricular electromechanical model of the murine heart, based on high-resolution experimentally measured fiber orientations. We introduced a strategy allowing to separate the macroscopic fiber architecture from local fiber disarray, and applied it to investigate their respective roles in electromechanical simulations. The resulting responses were compared with those obtained using a surrogate fiber field generated with a LDRBM approach [52]. Finally, we assessed the influence of fiber architecture on the different components of the model, including electrophysiology, passive mechanics, active contraction, and global hemodynamic indicators.

From the numerical results, we observed that the electrophysiology is only marginally sensitive to the presence of fiber disarray, whose main effect is to promote a more isotropic propagation of the activation wavefront (see Sec. 3.1). Moreover, the activation maps are well approximated even with the rule-based fibers, indicating that RBMs capture the macroscopic fiber architecture with sufficient accuracy to reproduce physiological activation patterns. However, these considerations apply only to ventricular morphology under physiological conduction conditions, and can substantially change in the atria and/or under pathological conditions, where altered conduction properties or heterogeneous substrates arise due to scar tissue or fibrosis [100].

The passive mechanical response is influenced by the macroscopic fiber architecture: replacing the experimental fiber field with the LDRBM configuration results in a volume variation of approximately 4% under purely passive loading at a pressure of 37.5 MPa. Instead, the effect of microscopic fiber disarray – represented in our analysis by increasing the smoothing parameter from ℓ=0 mm to ℓ=0.25 mm – is much weaker in the passive regime, leading to volume changes of only about 0.4%. For both macroscopic fiber architecture and microscopic disarray, the impact on passive mechanics is smaller than that observed in active contraction. A possible explanation is that active stress is primarily generated along the fiber direction and is therefore highly sensitive to fiber orientation. Conversely, passive elastic response is also supported by material stiffness in directions orthogonal to the fibers, resulting in a less pronounced anisotropy and, consequently, a reduced sensitivity to fiber orientation.

As a matter of fact, the greatest impact of fiber macroscopic architecture and microscopic disarray is observed in the electromechanical simulations (see Sec. 3.3.1), where active tension plays a central role in myocardial contraction. The PV loops differ substantially among the baseline (i.e., with the experimental fibers), regularized, and LDRBM cases. In particular, a regularization radius around 0.25 mm yields the maximum EF, indicating that the reduction of microscopic disarray enhances the mechanical efficiency of the pumping function. Further increasing the regularization radius, however, leads to a reduction of the LV EF, suggesting that excessive alteration of the macroscopic fiber organization impairs efficient ventricular contraction. This behavior suggests that the regularization radius ℓ can be interpreted as an effective length scale separating local fiber misalignment from the macroscopic myocardial organization contributing coherently to ventricular contraction. For small values of ℓ, smoothing primarily reduces microscopic disarray while preserving the physiological helical architecture, thereby promoting a more coordinated generation of active tension. Conversely, larger regularization radii progressively alter the macroscopic fiber organization itself, ultimately impairing mechanical efficiency. Interestingly, the optimal value identified in the present study (ℓ≈0.25 mm) is substantially smaller than the ventricular wall thickness and is instead closer to the characteristic scale of small cardiomyocyte aggregates, supporting the interpretation of ℓ as a local microstructural length scale rather than a macroscopic geometric one. Under this interpretation, the optimal smoothing scale may depend only weakly on organ size and species, although dedicated investigations on larger mammalian hearts would be required to verify this hypothesis quantitatively. Simulations performed with rule-based fibers and the same contractility parameter used for the experimental fibers exhibit even stronger contraction, ultimately leading to numerical failure due to the highly organized helical architecture.

We investigated two strategies commonly used in the cardiac modeling community to surrogate the effects of fiber disarray with an active stress approach (see Sec. 3.3.2): an effective reduction of contractility with respect to the pure longitudinal fiber contractile strength, and the introduction of active stress components in the sheet and/or normal directions. In this analysis, simulations with experimental fibers were taken as the reference baseline, with the aim of validating these strategies as practical modeling approaches to reproduce the behavior observed when high-resolution fiber data are available. The results showed that both strategies can yield pressure–volume loops close to the baseline. In particular, the best match was obtained with a 13% reduction in contractility, while when introducing active stress in orthogonal directions, the best agreement is obtained by activating both the sheetlet and cross-fiber components with a ratio SF=(0.97,0.17,0.17). However, despite the good agreement at the organ level, significant discrepancies emerge at the tissue level. In particular, the inclusion of active stress in cross-fiber directions leads to strain fields that differ markedly from the baseline case. On the other hand, a simple effective reduction of contractility, despite its phenomenological nature, is better able to reproduce the strain distributions. These findings highlight an intrinsic limitation of modeling approaches based on the addition of orthogonal active stress components. These results suggest that more faithful descriptions are likely to require rigorous upscaling procedures that link microscopic fiber distributions to effective macroscopic active stress formulations, which will be the subject of future work.

Finally, the regularized fiber fields consistently yield better agreement with the baseline results compared to the LDRBM, suggesting that an accurate representation of the macroscopic fiber architecture plays a dominant role compared with the explicit modeling of microscopic disarray.

Previous studies have established the capability of RBMs to reproduce signal propagation in the ventricles [52,101]. The analysis shown in the present work confirms these results and highlights the low impact of high frequency fibers misalignement in the signal propagation. Furthermore, our results suggest a negligible effect of fiber orientation on passive inflation. These findings are not comparable with those reported in [74], where the authors performed a sensitivity analysis of fiber orientation angles on diastolic mechanics. They observed a clear dependence of myocardium elastic properties on fiber mean orientation, considering large variations of the fiber angles at endocardium and epicardium. Nevertheless, their results are derived from a RBM and thus account solely for an idealized fiber architecture.

Regarding electromechanical simulations, our results highlight that a major impact of the myofiber orientations and related disarray. The observed increase in EDV [76] for fibers oriented in the longitudinal direction, the enhancement of EF in RBM cases [69], and the necessity to reproduce complete 3D fiber orientation for accurate strain distribution [75] are all consistent with literature findings [69,75]. Additionally, the effects of active tension misalignment in sheet and normal directions are in agreement with previous results [69,76]. In particular the active stress factor in the sheet direction decreases the EF, while the active stress factor in the normal direction induces an opposite effect [68,69].

Compared to previous studies, the present work represents, to the best of our knowledge, the first integration of fiber architecture data at this spatial resolution (96 μm) within a fully coupled electromechanical cardiac model. In addition, the proposed regularization framework enables a systematic decoupling of the effects of macroscopic fiber organization from the microscopic fiber disarray. This provides, for the first time, the opportunity to independently examine their functional roles and to critically evaluate commonly adopted modeling approaches for representing fiber disarray in electromechanical simulations.

A limitation of the present study is that the analysis was restricted to a single murine heart geometry. The present conclusions may therefore be influenced by specimen-specific factors, including inter-subject variability, tissue preparation and imaging uncertainty. More definitive and quantitative conclusions will require extending the investigation to a larger cohort of geometries in order to account for inter-subject variability. Nevertheless, the electromechanical results presented here are robust and highlight clear trends, allowing us to identify important limitations of both rule-based fiber models and indirect modeling approaches for fiber disarray. Caution is also required when extrapolating the present quantitative findings to larger mammalian hearts, due to well-known inter-species differences in ventricular geometry, wall thickness, and myocardial organization. Nevertheless, the proposed framework may provide useful insights for future investigations in larger animal models and human-specific geometries. Moreover, only physiological activation patterns were considered in this work. As a consequence, the conclusions drawn – in particular regarding electrophysiology – may not directly extend to pathological conditions, where altered conduction properties and heterogeneous substrates, such as scar or fibrosis, are expected to amplify the role of fiber architecture. Extending the present framework to pathological activation scenarios represents a relevant direction for future studies. In addition, the present analysis focused primarily on the LV. In murine hearts, RV mechanics are strongly influenced by ventricular interdependence and by the substantially lower pressure regime of the pulmonary circulation. Moreover, the thin RV free wall also results in a lower signal-to-noise ratio in the experimental fiber measurements. As a consequence, the conclusions drawn here regarding electromechanical function are more directly supported for the LV than for the RV.

While direct quantitative translation to the human heart is not straightforward due to well-known inter-species differences in structure and function, the computational framework developed here is species-agnostic and may provide a basis for future investigations in larger animal models and, eventually, in human-specific geometries, although the broader adoption of such approaches is currently limited by the availability of suitable high-resolution whole-heart datasets for large mammalian hearts. From a clinical perspective, these findings would provide a coherent framework to interpret the role of myocardial fiber architecture in the cardiac function, and are also relevant for the construction of cardiac digital twins, where fiber architecture is typically approximated rather than directly measured. While capturing the macroscopic organization of myocardial fibers appears sufficient to reproduce global electrophysiological features, it can lead to significant inaccuracies in predicting mechanical function if microscopic disarray is not well captured, particularly when active contraction and strain distributions are of interest.

## Supporting information

S1 Appendix Model calibration and baseline simulation setup. In this section, the baseline simulation and the corresponding parameters are detailed. Moreover, the calibration strategy for the electrophysiology, blood circulation, passive mechanics, and activation components is presented. First, we describe the employed experimental data, and then we illustrate the algorithm that we followed to calibrate all the components of the model, based on subject-specific activation maps, experimentally measured calcium and force traces, and on murine PV-loops observed in the literature (see [93,94]).
(PDF)

S2 Appendix Fiber distribution. In this section, an analysis of fiber distribution in terms of mean angles and disarray is presented.
(PDF)

S3 Appendix PV-loop analysis. In this section we complement the analysis presented in Sec. 3.3.1 on LV PV-loops with the PV-loops obtained by multiple simulations for RV, LA and RA. These plots are complementary to the LV ones shown in Fig 9.
(PDF)

S1 Table Parameters for baseline model. This table lists all the paramters, devided by core models, obtained trough calibration.
(TIF)

S1 Fig Calibration workflow for the biventricular electromechanical model of the murine heart. Arrows 1, 2, 3 and 4 represent the contribution of the four components in the electromechanical model. The green arrows (4, 5, and 6) depict a calibration loop that requires multiple iterations to calibrate the 0D circulation parameters. Arrows 7, 8, and 9 represent the final calibration loop for the crossbridge stiffness parameter.
(TIF)

S2 FigCalibration of the active force generation model for mouse cardiomyocytes.Top: experimentally measured calcium transient, averaged over five consecutive cycles. Center: comparison between the experimental twitch force and the model prediction after calibration of the activation parameters. The model reproduces the mechanical response by coupling the contractile element with a compliant elastic element adjusted to match the observed 10% shortening at a diastolic sarcomere length of 2.2 μm. Bottom: sarcomere length transient predicted by the model under the same experimental conditions.(TIF)

S3 FigDistribution of the α and γ angles in different zones of the domain.Comparing the experimental fiber field (ℓ=0 mm) with the regularized fiber field (ℓ=0.25 mm).(TIF)

S4 FigProjection of fiber field on the regularized one.Distibution of $\hat{\left|{𝐟}_{f}\right|}$, $\hat{\left|{𝐟}_{s}\right|}$ and $\hat{\left|{𝐟}_{n}\right|}$.(TIF)

S5 FigDistribution of the angles α and γ in the Experimental fiber field and in the smoothed fiber field.Histograms showing angle distributions (diagonal boxes) and scatterplots showing the cross-correlations (off-diagonal boxes).(TIF)

S6 FigPV-loops of RV, LA, RA.(first column) RV, (second column) LA, (third column) RA. (first Row) Comparison of PV-loops from experimental fiber field against regularized ones and the rule-based one, (second and third rows) recovery of the baseline PV-loop from regularized fiber field, (fourth and fifth rows) recovery of the baseline PV-loop from the rule-based one. The calibration is obtained varying (second and fourth rows) $a_{XB}$ and (third and fifth rows) SF.(TIF)

S7 FigDomain segmentation.Ω is the whole domain including also septum and myocardium that are not included in the image, (in orange) $\Gamma_{\text{base}}$ is the boundary surface identified as the ventricular base, (in gray) $\Gamma_{\text{epi}}$ is the boundary surface identified as the epicardium, (in blue) $\Gamma_{\text{endo, RV}}$ is the boundary syrface identified as the right endocardium, (in purple) $\Gamma_{\text{endo, LV}}$ is the boundary surface identified as the left endocardium.(TIFF)

S8 Fig Total activation time. The figure shows the total activation time, *T*_max_, as a function of the regularization radius and compares it with the value obtained using the rule-based fiber field.
(PNG)
