# Supplementary material for: The functional impact of myofiber macroscopic organization and disarray in computational models of the murine heart
Source: PLoS Comput Biol. 2026 Sep 21;22(9):e1014807. doi: 10.1371/journal.pcbi.1014807 (PMC13614661; doi:10.1371/journal.pcbi.1014807)
Supplement: Supplementary file 1 [file pcbi.1014807.s001.pdf]

S1 MODEL CALIBRATION AND BASELINE SIMULATION SETUP

In this section, the baseline simulation and the corresponding parameters are detailed. Moreover, the calibration strategy for the electrophysiology, blood circulation, passive mechanics, and activation components is presented. First, we describe the employed experimental data, and then we illustrate the algorithm that we followed to calibrate all the components of the model, based on subject-specific activation maps, experimentally measured calcium and force traces, and on murine PV-loops observed in the literature (see[1,2]).

S1.1 Baseline simulation setup

As explained in Sec. 3, the electromechanical model is composed of electrophysiology, activation, mechanics and circulation core models. Electrophysiology is modeled through the eikonal equation, whose solution is the activation time. The initial conditions of the eikonal problem are composed of 5 stimulation sites activated at different times to reproduce the Purkinje network function[3]. The calcium ion concentration curve is then translated in time at each point of the domain by a quantity equal to the activation time computed through the eikonal equation. Mechanical activation is described by the RDQ20-MF model, which allows to compute the active tension T_a through a system of ODEs[4]. Then, T_a enters in the definition of the active component of the Piola-Kirchhoff stress tensor $\mathbf{P}$ as shown in Eq. (20). In the baseline simulation, the active tension is assumed to act only in the longitudinal fiber direction, therefore the SF are set as $m_\mathrm{f} = 1$, $m_\mathrm{s} = 0$, $m_\mathrm{n} = 0$. We remark that T_a depends linearly on a_XB, which, in the baseline simulation, is set as $a_\mathrm{XB} = 23\,\mathrm{MPa}$. Finally, the 0D model of blood circulation is coupled with the mechanics model to provide coherent boundary conditions[5].

The full set of parameters is listed in Tab. I.

S1.2 Functional data for model calibration

The functional data used for model calibration were acquired from one control heart following the optical mapping protocol described in[7]. Briefly, the heart was excised from a heparinized and anesthetized male littermate control mouse, aged 28 weeks, cannulated at the aorta, and retrogradely perfused in a horizontal Langendorff configuration with oxygenated Krebs–Henseleit buffer at 36(1) °C. Cardiac contraction was suppressed by adding blebbistatin to the perfusate. After stabilization of the electrocardiogram, the voltage-sensitive dye di-4-ANBDQPQ was injected into the aorta over 1 min.

Transmembrane potential was imaged on the LV free wall using wide-field fluorescence microscopy. Excitation was provided by a 625 nm LED filtered at 640/40 nm, and the emitted fluorescence was collected through a 775/140 nm band-pass filter via a 685 nm dichroic beam splitter. Images were acquired on the central 128×128 pixels of an sCMOS camera (ORCA-Flash 4.0, Hamamatsu) at a frame rate of 1 kHz, yielding a pixel-size of $78\,\mu\mathrm{m} \times 78\,\mu\mathrm{m}$ in the sample. The heart was electrically paced at the apex using an electrode connected to an isolated constant-voltage stimulator, delivering 15 pulses at 5 Hz.

Activation maps were derived from the recorded fluorescence signals. The mean baseline was subtracted and the signal was normalized on a pixel-by-pixel basis to yield the fractional fluorescence change ($\Delta F/F_0$). After signal inversion, a spatial binning of 4×4 pixels was applied, resulting in parameter maps with a spatial resolution of 312 µm. Activation times were estimated using a cross-correlation-based approach: a reference pixel was selected, and the temporal shift of each pixel's fluorescence trace was computed relative to this reference, yielding a map of local activation delays.

The two-dimensional activation map was co-registered with the three-dimensional mesoSPIM-based anatomical reconstruction of the same heart, following the morpho-functional registration pipeline described in[7]. The registered activation map, projected onto the corresponding anatomical mesh, served as the reference for calibrating the eikonal model parameters.

S1.3 Calibration procedure

Fig. 1 shows a representation of the calibration procedure within an iterative framework. The calibration procedure comprises multiple steps. The first step focuses on calibrating the electrophysiology parameters. The second step involves computing an initial guess for the set of parameters of the 0D circulation model, by temporarily surrogating the ventricles by means of time-varying elastance models. The third step consists of calibrating Usyk's model parameters in the passive mechanics model. Then the activation model is calibrated and a first guess for the crossbridge stiffness

Component	Model Parameter	Value	Method parameter	Value
**Electrophysiology**	Electrophysiology approach	Eikonal	FE space degree	1
	Longitudinal conductivity σ_{f}	$2.0 \times 10^{-4}\,\mathrm{m^2s^{-1}}$	Algorithm	Continuation
	Depolarization coefficient c_0	$55\,\mathrm{s^{-1/2}}$	Initial ramp step	0.1
	Transversal conductivity σ_{s}	$1.0 \times 10^{-4}\,\mathrm{m^2s^{-1}}$	Step factor after failure	0.5
	Cross-fiber conductivity σ_{n}	$1.0 \times 10^{-4}\,\mathrm{m^2s^{-1}}$	Step factor after success	1.2
	Conduction velocity[6] $v_f = c_0\sqrt{\sigma_f}$	$0.777\,\mathrm{ms^{-1}}$	Maximum ramp increment	1
	$v_s = v_n = c_0\sqrt{\sigma_s}$	$0.549\,\mathrm{ms^{-1}}$	Minimum ramp increment	0
			Linear solver	GMRES,
				Maximum iterations =1000,
				Tolerance=1e-15,
**Fibers**	Regularization radius ℓ	$0.0\,\mathrm{mm}$	Element type	Tet
**Mechanics**	Density	$1 \times 10^3\,\mathrm{kgm^{-3}}$	FE space degree	1
	Viscosity	$0\,\mathrm{Pa\,s}$	Pericardium Robin BC	$K_{\mathrm{norm}} = 30 \times 10^5\,\mathrm{m^{-1}Pa}$,
	Fiber active stress factor m_{f}	1.0		$K_{\mathrm{tan}} = 30 \times 10^4\,\mathrm{m^{-1}Pa}$,
	Sheet active stress factor m_{s}	0.0		$C_{\mathrm{norm}} = 6 \times 10^4\,\mathrm{sm^{-1}Pa}$,
	Cross active stress factor m_{n}	0.0		$C_{\mathrm{tan}} = 6 \times 10^3\,\mathrm{sm^{-1}Pa}$.
			Base Robin BC	$K_{\mathrm{norm}} = 2 \times 10^5\,\mathrm{m^{-1}Pa}$,
	Usyk parameters	$b_{\mathrm{f}} = 5.0$, $b_{\mathrm{s}} = 3.7$,		$K_{tan} = 2 \times 10^5\,\mathrm{m^{-1}Pa}$,
		$b_{\mathrm{n}} = 3.7$, $b_{\mathrm{fs}} = 2.6$,		$C_{\mathrm{norm}} = 2 \times 10^4\,\mathrm{sm^{-1}Pa}$,
		$b_{\mathrm{fn}} = 3.7$, $b_{\mathrm{sn}} = 3.7$,		$C_{\mathrm{tan}} = 2 \times 10^3\,\mathrm{sm^{-1}Pa}$.
		bulk $= 5 \times 10^4$ Pa, $c = 2 \times 10^3$ Pa	Non-linear solver	Maximum iterations =50,
				Absolute tolerance=1e-9,
				Relative tolerance=1e-9
			Linear solver	GMRES,
				Maximum iterations=1000,
				Tolerance=1e-8,
**Activation**	LV contractility ratio	1.0	FE space degree	1
	RV contractility ratio	0.5	Time step (0D)	$2 \times 10^{-5}\,\mathrm{s}$
	Sarcomere slack length	$1.9\,\mathrm{\mu m}$	Time step RU update	$2.0 \times 10^{-5}\,\mathrm{s}$
	Model	Activation-RDQ20-MF	Linear solver	GMRES,
	Sarcomere initial length	$2.2\,\mathrm{\mu m}$		Maximum iterations=1000,
	Calcium initial concentration	$0.1656\,\mathrm{\mu molL^{-1}}$		Tolerance=1e-10,
	Initialization time	$0.2\,\mathrm{s}$		
	Sarcomere parameters	$LA = 1.25\,\mathrm{\mu m}$, $LM = 1.65\,\mathrm{\mu m}$,		
		$LB = 0.18\,\mathrm{\mu m}$, $\mu_{0,\mathrm{fP}} = 32.68\,\mathrm{s^{-1}}$,		
		$SL_0 = 2.2\,\mathrm{\mu m}$, $Q = 2$,		
		$K_{\mathrm{d},0} = 0.65\,\mathrm{\mu m}$, $\alpha_{\mathrm{Kd}} = -0.83\,\mathrm{\mu molL^{-1}\mu m^{-1}}$,		
		$\mu = 10$, $\gamma = 12$,		
		$K_{\mathrm{off}} = 180\,\mathrm{s^{-1}}$, $K_{\mathrm{basic}} = 180\,\mathrm{s^{-1}}$,		
		$r_0 = 134.3\,\mathrm{s^{-1}}$, $\alpha = 25.18$,		
		$\mu_{1,\mathrm{fP}} = 0.77\,\mathrm{s^{-1}}$, $a_{\mathrm{XB}} = 15 \times 10^6$ Pa,		
		$TnC_{\mathrm{max}} = 70\,\mathrm{\mu molL^{-1}}$		
**Circulation**	Heartbeat period	$0.2\,\mathrm{s}$	Numerical scheme	Explicit Euler
	Right atrium	Elastance $A = 1000\,\mathrm{mmHgmL^{-1}}$,		
		$B = 40\,\mathrm{mmHgmL^{-1}}$, $V_0 = 0.0008\,\mathrm{mL}$,		
		Duration of contraction = 0.38,		
		Duration of relaxation = 0.38,		
		$t_0 = 0.83$,		
	Pulmonary arterial system	$R = 5.216\,\mathrm{mmHgsmL^{-1}}$, $C = 0.0024\,\mathrm{mLmmHg^{-1}}$,		
		$R_{\mathrm{up}} = 0.0\,\mathrm{mmHgsmL^{-1}}$, $L = 2064 \times 10^{-4}\,\mathrm{mmHgs^2mL^{-1}}$		
	Pulmonary venous system	$R = 55.45\,\mathrm{mmHgsmL^{-1}}$, $C = 0.0387\,\mathrm{mLmmHg^{-1}}$		
		$L = 2064 \times 10^{-4}\,\mathrm{mmHgs^2mL^{-1}}$		
	Left atrium	Elastance $A = 140\,\mathrm{mmHgmL^{-1}}$, $B = 360\,\mathrm{mmHgmL^{-1}}$,		
		Duration of contraction = 0.36,		
		Duration of relaxation = 0.36,		
		$t_0 = 0.85$, $V_0 = 0.0008\,\mathrm{mL}$		
	Systemic arterial system	$R = 500\,\mathrm{mmHgsmL^{-1}}$, $C = 0.0008\,\mathrm{mLmmHg^{-1}}$,		
		$R_{\mathrm{up}} = 0.0\,\mathrm{mmHgsmL^{-1}}$, $L = 2064 \times 10^{-3}\,\mathrm{mmHgs^2mL^{-1}}$,		
	Systemic venous system	$R = 400\,\mathrm{mmHgsmL^{-1}}$, $C = 0.0145\,\mathrm{mLmmHg^{-1}}$,		
		$L = 2064 \times 10^{-4}\,\mathrm{mmHgs^2mL^{-1}}$		
	Valves	$R_{\mathrm{min}} = 7.5\,\mathrm{mmHgsmL^{-1}}$, R_{max}=75 000 $\mathrm{mmHgsmL^{-1}}$,		
**LDRBM**	Imposed angles	$\alpha_{\mathrm{Epi,LV}} = 23^\circ$, $\alpha_{\mathrm{Epi,RV}}$, $\alpha_{\mathrm{Endo,LV}} = -81^\circ$,		
		$\alpha_{\mathrm{Endo,RV}}$, $\gamma_{\mathrm{Epi,LV}} = 2^\circ$, $\gamma_{\mathrm{Epi,RV}} = 5^\circ$,		
		$\gamma_{\mathrm{Endo,LV}} = 5.4^\circ$, $\gamma_{\mathrm{Endo,RV}} = 5^\circ$		

Table I. Parameters for Baseline Model

is computed. Afterward, the four core models are coupled in a comprehensive electromechanical simulation. From this model, a 0D emulator[8] is built and the 0D circulation model calibration is fine-tuned together with the value of a_{XB}.

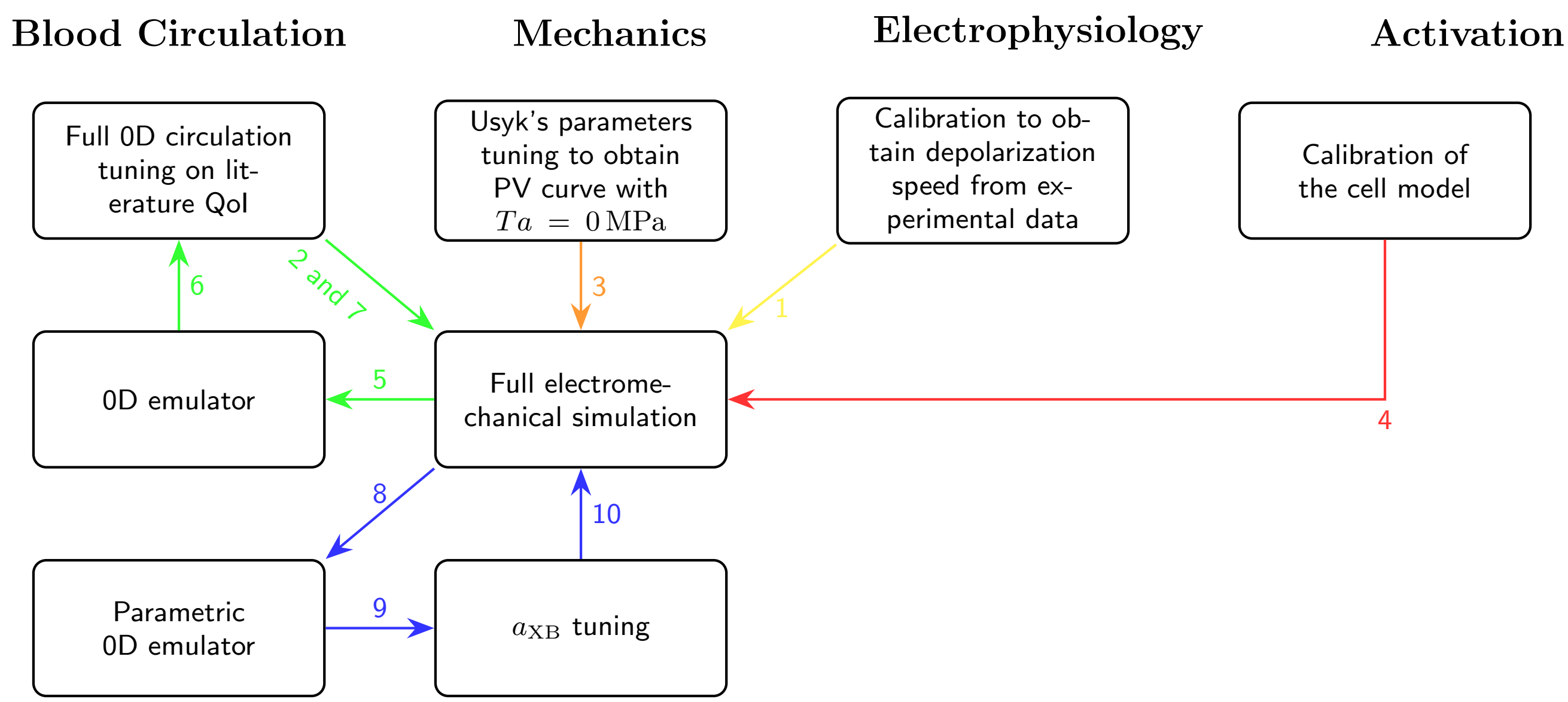

Figure 1. Calibration workflow for the biventricular electromechanical model of the murine heart. Arrows 1, 2, 3 and 4 represent the contribution of the four components in the electromechanical model. The green arrows (4, 5, and 6) depict a calibration loop that requires multiple iterations to calibrate the 0D circulation parameters. Arrows 7, 8, and 9 represent the final calibration loop for the crossbridge stiffness parameter.

S1.3.1 Electrophysiology

The eikonal model used in this work is calibrated based on an experimental activation map obtained through stimulation of the ventricular apex and imaging of the resulting fluorescence. From this measurement, the average depolarization speed in the mouse tissue is computed. Then, the conductivity parameters of the eikonal problem are adjusted to recover the experimental depolarization speed (reported in Tab I).

S1.3.2 Blood-circulation

The blood circulation model is calibrated based on ventricular PV-loops observed in literature[1,2]. In the first step of the circulation calibration, the ventricles are modeled as elastances $E(t)$ varying in time t. Therefore, the pressure curve in each chamber can be computed as

$$P(V,t) = E(t)(V - V_0), \tag{1}$$

where P is the pressure, V is the blood volume and V_0 is the resting volume of the chamber. We solve multiple times the circulation model, coupled with Eq. (1), to compute an initial guess of the circulation parameters.

Following the calibration of the passive mechanics model, activation and electrophysiology, we use the 0D emulator proposed in[8] to further calibrate the circulation parameters and speed up the convergence of the 3D model. Specifically, the emulator can take advantage of 3D electromechanical simulations to build a 0D associated counterpart of the chamber (i.e. LV) that reproduces the same PV-loop obtained in the 3D simulation. The latter is based on the assumption that the PV-loop of the chamber can be approximated by the weighted sum of the end-systolic pressure-volume relationship (ESPVR) and of the end-diastolic pressure-volume relationship (EDPVR). Therefore the ventricular pressure P_{V} can be written as

$$P_{\text{V}}^{\text{emu}}(V,t) = (1 - \varphi_{\text{act}}(t))P_{\text{ED}}(V) + \varphi_{\text{act}}(t)P_{\text{ES}}(V) \tag{2}$$

where $P_{\text{ED}}(V)$ is the EDPVR, $P_{\text{ES}}(V)$ is the ESPVR and the function φ_{act} is a time-dependent function encoding the activation kinetics. Ideally, $\varphi_{act} = 0$, $\varphi_{\text{act}} = 1$ corresponding to fully relaxed (at end-diastole) and fully contracted (at end systole) tissue respectively. φ_{act} is periodic in t with period T_{HB}, that is the duration of a heartbeat. Note that inertia and damping effects are neglected within the 0D emulator. The procedure for building the emulator and reaching the limit cycle to be used in the 3D simulation consists of the following steps.

1. Run a full 3D simulation with an acceptable number of hearbeats (5 for this work).

2. Build the 0D emulator of the 3D chamber using the PV-loop derived from the 3D simulation.

3. Run a 0D circulation simulation until the limit cycle is approached. During this step, the circulation parameters are calibrated in order to obtain a physiological PV-loop, based on those reported in the literature[1,2]. This procedure is run in real time, avoiding running multiple simulations to calibrate the circulation parameters.

4. Use the obtained PV-loops as initial conditions of the new 3D simulation.

This method, which is called 3D-0D-3D V-cycle[5], is used to obtain the limit cycle of the calibrated circulation model in far less time than it would be required through multiple trials on the 3D simulations. The emulator, therefore, serves a dual role. Firstly, it speeds up the convergence process by skipping the computationally expensive transient behavior, enabling the 3D model to reach a steady state with far fewer simulated heartbeats. Moreover, it allows for efficient many-query scenarios, such as sensitivity analysis, parameter estimation, and uncertainty quantification, where the cardiac model is, in principle, multiple times for different parameter sets.

1. *S1.3.3 Activation*

The active force generation model RDQ20-MF was originally calibrated for rat and human cardiomyocytes[4]. Therefore, its application to mouse cells requires a dedicated recalibration procedure. Since this process relies on detailed measurements obtained under specific conditions – such as physiological temperature – that were not available for the same animal model used here, we rely instead on data acquired from mice of comparable age at 37 °C, with extracellular calcium concentration 1.8 µM[9]. Specifically, we use calcium transient measurements recorded during twitch contractions at 5 Hz. To reduce noise, the fluorescence signal is averaged over five consecutive cycles. To convert the Cal520 fluorescence signal $F(t)$ into intracellular calcium concentration, we use the standard relation $[\mathrm{Ca}^{2+}]_\mathrm{i}(t) = K_d \frac{F(t)-F_\mathrm{min}}{F_\mathrm{max}-F(t)}$ where $K_d = 320\,\mathrm{nM}$ represents the dissociation constant of the dye, while $F_\mathrm{max} = 11110$ and $F_\mathrm{min} = 3600$ denote, respectively, the fluorescence levels at calcium saturation and at zero calcium. The resulting calcium trace is then used to calibrate the RDQ20-MF model. To this end, we employed mechanical experiments performed on myofibers extracted from the same cell, consisting of shortening twitches characterized by a diastolic sarcomere length of 2.2 µm and a shortening amplitude of 10%. To model the experimental setup, we consider an elastic element in series with the contractile element, that takes into account the compliant portion of the sample. The stiffness of this elastic element is calibrated to match the observed shortening for a given developed force. Finally, the parameters of the activation model – specifically those governing the kinetics (k_on, k_basic) and the cross-bridge contractility (a_XB) – are calibrated to minimize the mismatch between the predicted and measured force traces as shown in Fig. 2.

S1.3.4 Mechanics

For the passive mechanics, we start from Usyk's parameter values obtained by the authors in[10], where a murine LV is inflated and the resulting pressure–volume curve is fitted by varying the stiffness parameters. Then, in order to match the literature passive pressure–volume relationship with the geometry used in the present study, which is characterized by a thin RV wall, we vary the parameter c in Usyk strain potential[11]. Regarding the active part, to calibrate the crossbridge stiffness a_XB, used in Eq. (21) a specific strategy is employed by using a combination of two emulators. This approach involves constructing two distinct emulators, each based on a different value for a particular parameter. Consequently, two separate full-scale simulations are used as datasets for emulator training. The two emulators are subsequently interpolated to create a parametric emulator, adding an extra degree of freedom. In particular given two different simulations, namely A and B, with $a_\mathrm{XB} = a_\mathrm{XB,A}$ and $a_\mathrm{XB} = a_\mathrm{XB,B}$ respectively, the parametrized pressure curve $p_{param}(V, t, a_\mathrm{XB})$ becomes

$$p_\mathrm{param}(V, t, a_\mathrm{XB}) = \frac{a_\mathrm{XB} - a_\mathrm{XB,A}}{a_\mathrm{XB,B} - a_\mathrm{XB,A}} p_\mathrm{B}(V, t) + \frac{a_\mathrm{XB} - a_\mathrm{XB,B}}{a_\mathrm{XB,A} - a_\mathrm{XB,B}} p_\mathrm{A}(V, t). \tag{3}$$

We use this formulation to fit the PV-loops available in the literature[1,2] by varying a_XB.

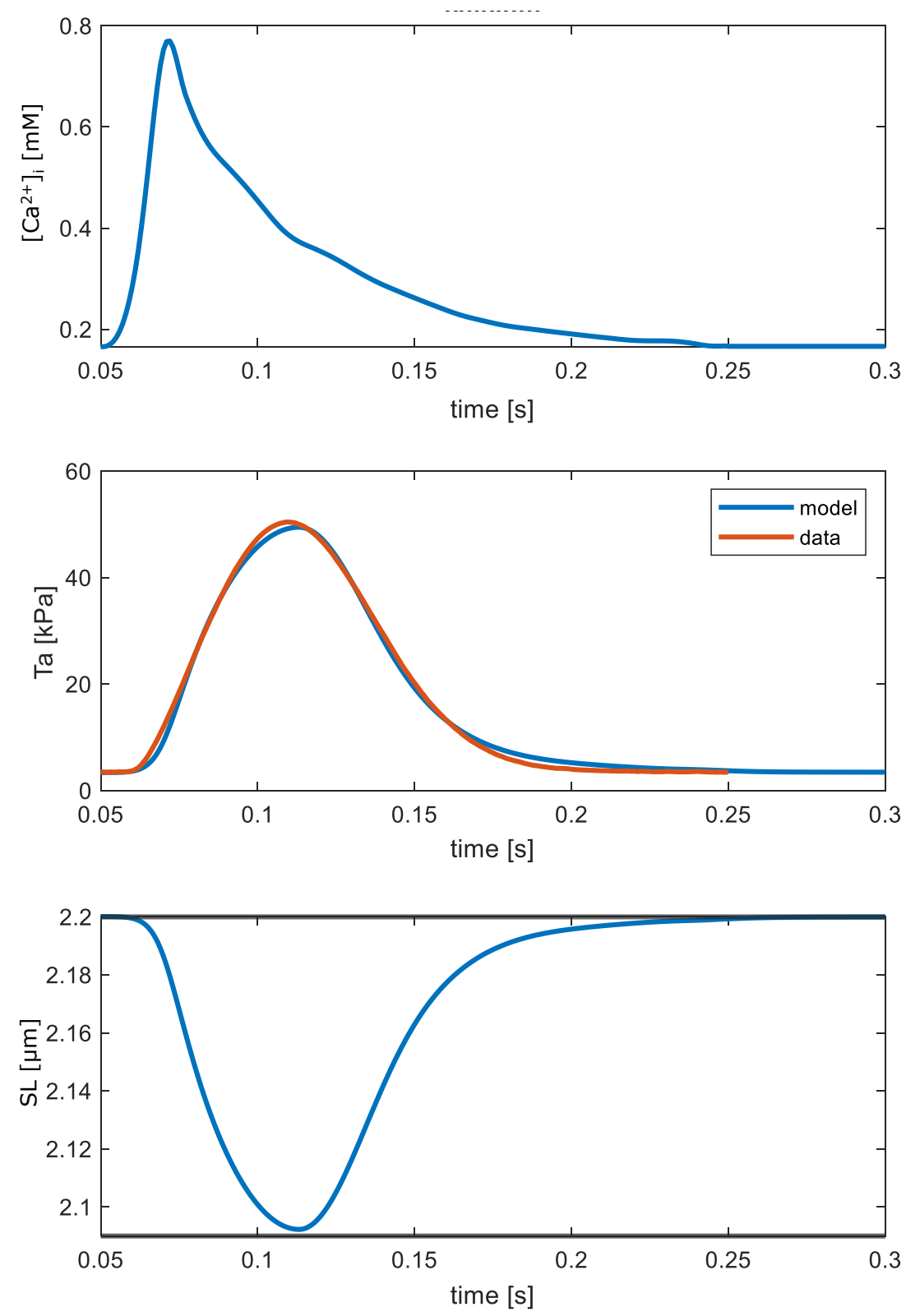

Figure 2. Calibration of the active force generation model for mouse cardiomyocytes. Top: experimentally measured calcium transient, averaged over five consecutive cycles. Center: comparison between the experimental twitch force and the model prediction after calibration of the activation parameters. The model reproduces the mechanical response by coupling the contractile element with a compliant elastic element adjusted to match the observed 10% shortening at a diastolic sarcomere length of 2.2 µm. Bottom: sarcomere length transient predicted by the model under the same experimental conditions.

Tab I lists all the models and methods parameters used in the baseline simulation.

---

[1] D. M. Tabima, T. A. Hacker, and N. C. Chesler, American Journal of Physiology-Heart and Circulatory Physiology **299**, H2069 (2010).
[2] D. Townsend, Journal of visualized experiments , 53810 (2016).
[3] S. Stella, F. Regazzoni, C. Vergara, L. Dede, and A. Quarteroni, Mathematical Models and Methods in Applied Sciences **32**, 1531 (2022).
[4] F. Regazzoni, L. Dedè, and A. Quarteroni, PLoS computational biology **16**, e1008294 (2020).
[5] R. Piersanti, F. Regazzoni, M. Salvador, A. F. Corno, C. Vergara, A. Quarteroni, *et al.*, Computer Methods in Applied Mechanics and Engineering **391**, 114607 (2022).
[6] R. Piersanti, R. Bradley, S. Y. Ali, A. Quarteroni, N. A. Trayanova, *et al.*, Computers in biology and medicine **188**, 109774 (2025).
[7] F. Giardini, C. Olianti, G. A. Marchal, F. Campos, V. Romanelli, J. Steyer, J. Madl, R. Piersanti, G. Arecchi, I. Perumal Vanaja, *et al.*, Nature Cardiovascular Research , 1 (2025).
[8] F. Regazzoni and A. Quarteroni, Computers in Biology and Medicine **135**, 104641 (2021).
[9] C. Ferrantini, R. Coppini, B. Scellini, C. Ferrara, J. M. Pioner, L. Mazzoni, S. Priori, E. Cerbai, C. Tesi, and C. Poggesi, Journal of General Physiology **147**, 39 (2016).
[10] Ø. Nordbø, P. Lamata, S. Land, S. Niederer, J. M. Aronsen, W. E. Louch, I. Sjaastad, H. Martens, A. B. Gjuvsland, K. Tøndel, *et al.*, Computers in biology and medicine **53**, 65 (2014).
[11] T. Usyk, R. Mazhari, and A. McCulloch, Journal of elasticity and the physical science of solids **61**, 143 (2000).
