# Supplementary material for: The functional impact of myofiber macroscopic organization and disarray in computational models of the murine heart
Source: PLoS Comput Biol. 2026 Sep 21;22(9):e1014807. doi: 10.1371/journal.pcbi.1014807 (PMC13614661; doi:10.1371/journal.pcbi.1014807)
Supplement: Supplementary file 2 [file pcbi.1014807.s002.pdf]

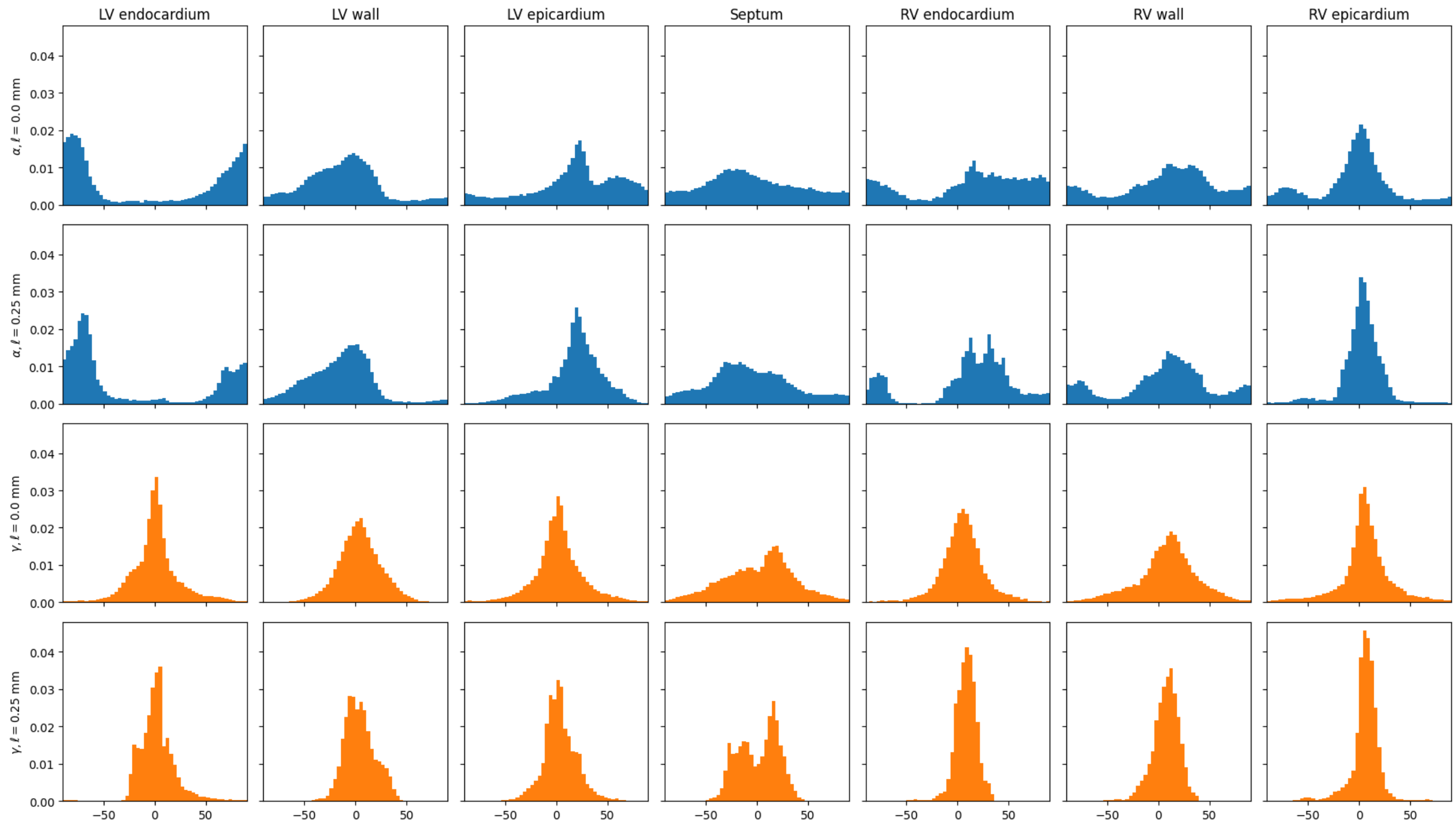

Figure 1. Distribution of the α and γ angles in different zones of the domain, comparing the experimental fiber field ($\ell = 0\,\mathrm{mm}$) with the regularized fiber field ($\ell = 0.25\,\mathrm{mm}$).

S2 FIBER DISTRIBUTION

In this section, an analysis of fiber distribution in terms of mean angles and disarray is presented.

Fig. 1 shows the distribution of the α and γ angles in different regions of the biventricular geometry for the experimental fiber field ($\ell = 0\,\mathrm{mm}$) and for the smoothed fibers ($\ell = 0.25\,\mathrm{mm}$). The angle γ exhibits mode values close to zero and its variance is much smaller with respect to α. Furthermore, the smoothing process reduces the variance of the angles in the whole domain. In particular, the standard deviation is equal to 45.68° for α and to 25.39° for γ in the experimental fiber field, while in the smoothed case the standard deviation decreases to 40.20° for α and to 15.97° for γ. The distribution of the angles varies largely across different regions of the myocardium. The largest variation of the modal value of α is measured in the LV passing from the endocardium (−76°), through the wall (−1.8°) to the epicardium (22°), while in the zone of the RV the variation is consierably lower (from 5° to 14°), given the thinner wall as shown in Fig. 1.

Fig. 3 shows the correlation of the α and γ angles in the experimental field and in the smoothed one with $\ell = 0.25\,\mathrm{mm}$. The plots show good correlation between the measured fibers and the smoothed one. In addition, we observe that the angles α and γ are only weakly correlated between each other, as the angle γ remains close to zero while α exhibits a much larger variation.

To assess the average fiber misalignment, the measured fiber field was projected onto the smoothed field $(\mathbf{f}_0, \mathbf{s}_0, \mathbf{n}_0)$, resulting in three components: $\mathbf{f}_f = \mathbf{f}^{\mathrm{meas}} \cdot \mathbf{f}_0$, $\mathbf{f}_s = \mathbf{f}^{\mathrm{meas}} \cdot \mathbf{s}_0$ and $\mathbf{f}_n = \mathbf{f}^{\mathrm{meas}} \cdot \mathbf{n}_0$. Fig, 2 shows the absolute value distribution of the three components in each zone of the domain. Given the means $|\hat{\mathbf{f}}_f|$, $|\hat{\mathbf{f}}_s|$ and $|\hat{\mathbf{f}}_n|$, the distance $1 - |\hat{\mathbf{f}}_f|$ and the values of $|\hat{\mathbf{f}}_s|$ and $|\hat{\mathbf{f}}_n|$ are measures of the myofiber disarray. In particular, $|\hat{\mathbf{f}}_f|$ varies from a maximum of 0.92 in the left endocardium to a minimum of 0.85 in the right endocardium. In the whole myocardium we measure mean values $|\hat{\mathbf{f}}_f| = 0.89$,$|\hat{\mathbf{f}}_s| = 0.18$, $|\hat{\mathbf{f}}_s| = 0.19$. These coefficients are set in active stress factors in the configuration SF$= (0.89, 0.19, 0.18)$ for the Piola-Kirchhoff stress tensor (see Eq. (20)).

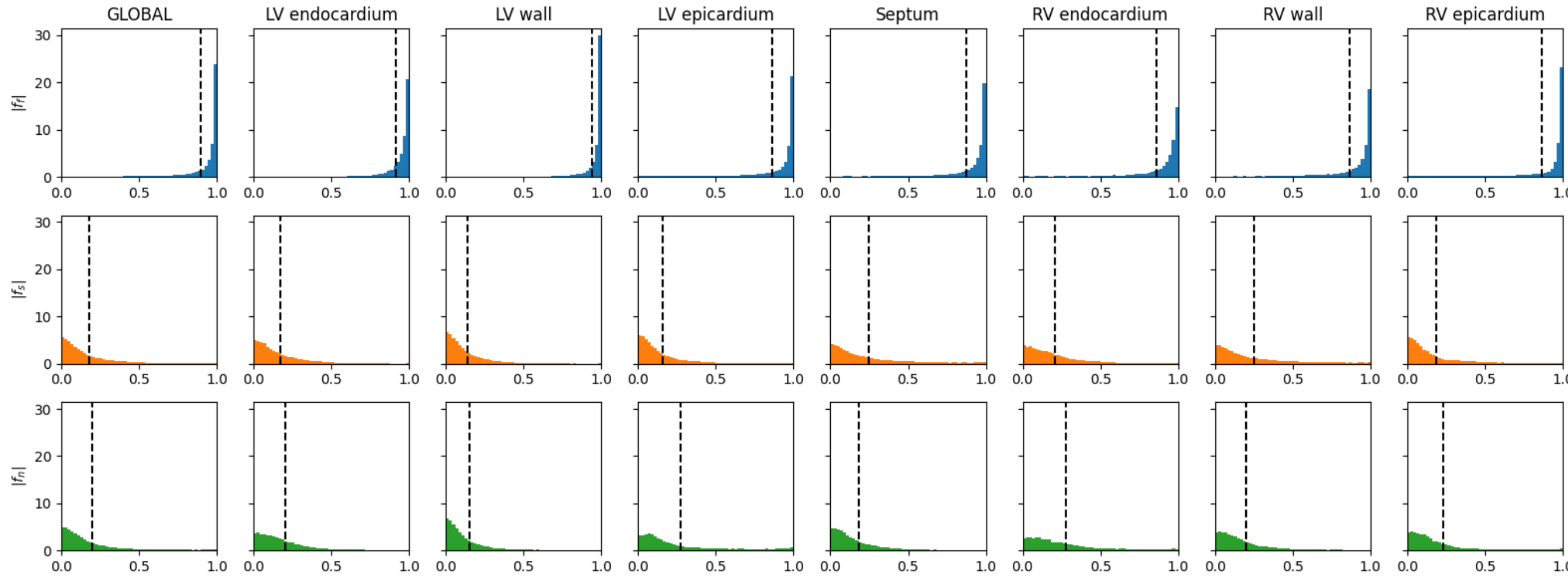

Figure 2. Distibution of $|\hat{\mathbf{f}}_f|$, $|\hat{\mathbf{f}}_s|$ and $|\hat{\mathbf{f}}_n|$.

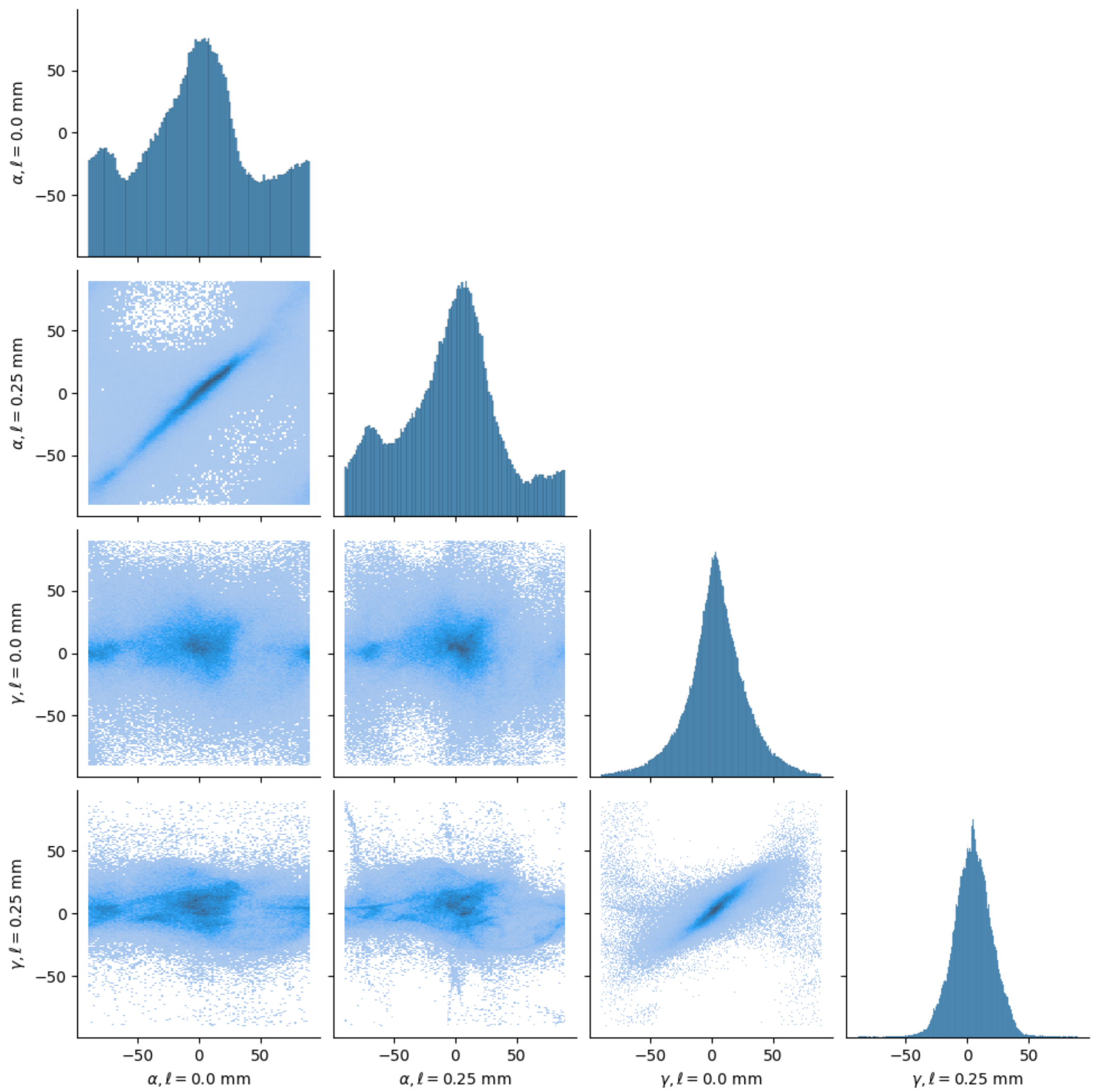

Figure 3. Distribution of the angles α and γ in the Experimental fiber field and in the smoothed fiber field: histograms showing angle distributions (diagonal boxes) and scatterplots showing the cross-correlations (off-diagonal boxes).
