# Supplementary material for: The functional impact of myofiber macroscopic organization and disarray in computational models of the murine heart
Source: PLoS Comput Biol. 2026 Sep 21;22(9):e1014807. doi: 10.1371/journal.pcbi.1014807 (PMC13614661; doi:10.1371/journal.pcbi.1014807)
Supplement: Supplementary file 3 [file pcbi.1014807.s003.pdf]

S3 PV-LOOP ANALYSIS

In this section we complement the analysis presented in Sec. 4.3.1 on LV PV-loops with the PV-loops obtained by multiple simulations for RV, LA and RA. These plots are complementary to the LV ones shown in Fig. 9. In particular, the first row of Fig. 1 shows the PV-loops obtained varying the regularization radius compared to the ones of the LDRBM. In the second and third rows of Fig. 1 we aim to recover the LV PV-loops of the experimental fiber field and the regularized one with $\ell = 0.25\,\mathrm{mm}$, varying the a_{XB} and SF. Fig. 1 in the fourth and fifth rows shows the PV-loops of the experimental fiber field and the LDRBM ones. In the RV we observe a shift to the right when increasing the regulrization radius up to $\ell = 1.0\,\mathrm{mm}$, while the opposite trend is observed for $\ell = 1.5\,\mathrm{mm}$. A similar behaviour is observed in RA where the trend inversion is already visible for $\ell = 1.5\,\mathrm{mm}$. We conclude that the smoothing of the fiber field does not causes an increase in the RA and RV efficiency. These results are consistent with the fact that the contraction of the RV is strongly driven by LV. Because the atria are represented by a 0D circulation model, the observed PV loops directly reflect the pressure and volume changes in the ventricles. The LA PV-loop follows the LV behaviour with a left shift up to $\ell = 0.25\,\mathrm{mm}$ and then a trend inversion. We recall that the LDRBM simulation with SF and a_{XB} equal to the baseline failed due to excessive contraction in the LV. In the second and third rows we can observe that transversal and cross-fiber SF activation and a_{XB} produce comparable effect on RV and RA with respect to LV. In fact the reduction of a_{XB} causes a shift to the right of the PV-loop for all the chambers, as the the activation of the sheet SF, while the activation of cross-fiber SF causes a shift to the left. However, the magnitude of this effect is considerably lower with respect to LV. Passing from $a_{\mathrm{XB}} = 23\,\mathrm{MPa}$ to $a_{\mathrm{XB}} = 20\,\mathrm{MPa}$ causes a reduction of EF of RV from 65% 62%, while passing from the SF=(1,0,0) to the SF=(0.99,0.14,0.00) reduces the EF from 60% to 58%. Therefore while the LV PV-loop get closer to the baseline, the RV one shifts away from the baseline. Similar results are also observed in the fourth and fifth rows, where the RV PV loops of the LDRBM are shifted to the right compared to the baseline simulation. This behaviour contrasts with that of the LV, where the LDRBM is more efficient than the baseline. These observations indicate that, with the tested methodologies, it is not possible to simultaneously recover the LV and RV PV loops using the LDRBM and the regularized fiber field. This confirms the need for improved methodologies that account for fiber disarray and overcome the lack of patient-specific data for fiber field reconstruction.

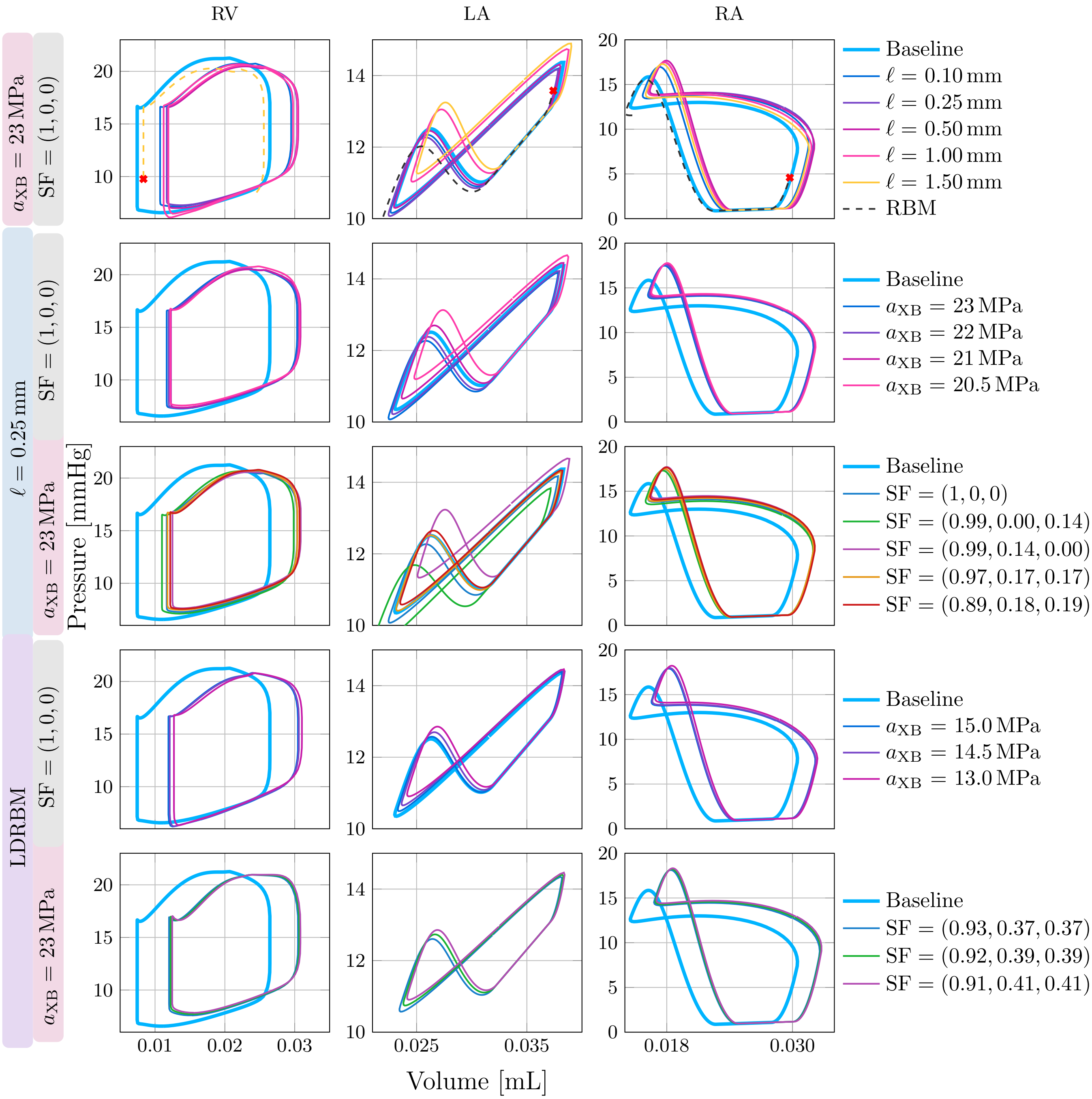

Figure 1. PV-loops of (first column) RV, (second column) LA, (third column) RA. (first Row) Comparison of PV-loops from experimental fiber field against regularized ones and the rule-based one, (second and third rows) recovery of the baseline PV-loop from regularized fiber field, (fourth and fifth rows) recovery of the baseline PV-loop from the rule-based one . The calibration is obtained varying (second and fourth rows) a_{XB} and (third and fifth rows) SF.
