# Supplementary material for: The functional impact of myofiber macroscopic organization and disarray in computational models of the murine heart
Source: PLoS Comput Biol. 2026 Sep 21;22(9):e1014807. doi: 10.1371/journal.pcbi.1014807 (PMC13614661; doi:10.1371/journal.pcbi.1014807)
Supplement: Supplementary file 4 [file pcbi.1014807.s013.pdf]

July 3, 2026

The functional impact of myofiber macroscopic organization and disarray in computational models of the murine heart
PLOS Computational Biology - Manuscript Number PCOMPBIOL-D-26-00721

Answers to Reviewers

Dear Editor,

We thank the Reviewers for carefully reading our paper and for their relevant suggestions. We reviewed the text accordingly and we report hereafter a detailed description of all the modifications carried out upon Reviewers' requests. The pages and references mentioned in our answers refer to the revised manuscript. The main modifications in the paper are highlighted in blue.

With our best regards,

Carlo Guastamacchia, Roberto Piersanti, Francesco Giardini, Raffaele Coppini, Cecilia Ferrantini, Luca Dede', Leonardo Sacconi, Francesco Regazzoni

Reviewer #1

> *The manuscript titled "The functional impact of myofiber macroscopic organization and disarray in computational models of the murine heart" presents an exceptional study that leverages high-resolution optical imaging data to decouple macroscopic fiber architecture from local disarray. This work addresses a fundamental and long-standing limitation in cardiac digital twins—the reconstruction of fiber fields. The introduction of the Helmholtz spatial filtering strategy is novel and provides critical insights into the limitations of widely used Rule-Based Methods (LDRBM) and cross-fiber active stress surrogates.*
> *The paper is well-written, the methodology is rigorous, and the findings are of significant importance to the computational cardiology community. The results convincingly demonstrate that global electrophysiology and passive mechanics are less sensitive to disarray compared to active contraction, and that a reduction in effective contractility is a more accurate phenomenological surrogate than orthogonal stress components.*
> *I recommend Minor Revision to address the following two points, which primarily require clarification and extended discussion in the text rather than new experiments.*

We are grateful to the Reviewer for carefully reading the manuscript and for the constructive suggestions.

> *1. **Generalizability and Single-Sample Limitations.** The study is based on a single high-resolution ex vivo murine heart dataset, which is used as the reference ground truth. This is a valuable dataset, but it would be helpful if the authors could discuss more explicitly the extent to which the conclusions may depend on this specific specimen. In particular, inter-subject variability, preparation-related effects, imaging uncertainty, and the specific structural features of the murine heart may influence the relative roles of macroscopic fiber organization and microscopic disarray reported here. A clearer discussion of these limitations in the Discussion section, particularly regarding the generalizability to larger mammalian hearts, would improve the manuscript.*

We thank the Reviewer for the insightful comments. We have extended the limitations section accordingly:

> A limitation of the present study is that the analysis was restricted to a single murine heart geometry. The present conclusions may therefore be influenced by specimen-specific factors, including inter-subject variability, tissue preparation and imaging uncertainty. More definitive and quantitative conclusions will require extending the investigation to a larger cohort of geometries in order to account for inter-subject variability. Nevertheless, the electromechanical results presented here

> are robust and highlight clear trends, allowing us to identify important limitations of both rule-based fiber models and indirect modeling approaches for fiber disarray. Caution is also required when extrapolating the present quantitative findings to larger mammalian hearts, due to well-known inter-species differences in ventricular geometry, wall thickness, and myocardial organization. Nevertheless, the proposed framework may provide useful insights for future investigations in larger animal models and human-specific geometries.

2. ***Physical Interpretation of the Optimal Smoothing Parameter (ℓ).*** *The authors have identified an optimal parameter value ($\ell = 0.25$ mm), but the manuscript does not currently elaborate on why this particular value emerges. Is it because it corresponds to roughly one-quarter of the murine wall thickness (1 mm)? Does it reflect the characteristic width of a few cardiomyocytes? Or is it an empirical optimum specific to this dataset? Given that the human ventricular wall is approximately $10 - 15$ millimeter thick, this raises an important question for readers who may wish to apply this method to other species: Should the optimal ℓ be scaled proportionally (e.g., to $2.5 - 3.75$ mm for humans), or should it be treated as an absolute constant linked to a species-invariant cellular length scale ($\sim 100\,\mu$m)? I encourage the authors to include a short discussion in the manuscript addressing this point to enhance the methodological clarity and cross-species applicability of their framework.*

We agree that the physical interpretation and potential cross-species scaling of the optimal smoothing parameter ℓ deserve clarification. In the revised manuscript, we have expanded the Discussion to better explain the mechanistic interpretation of ℓ as an effective length scale separating local fiber disarray from the coherent macroscopic fiber organization contributing to ventricular contraction. We also clarified that the optimal value identified in the present study emerges empirically from the analyzed dataset, while noting that its magnitude is more consistent with a local microstructural scale (i.e. small cardiomyocyte aggregates) than with the global ventricular wall thickness. The newly added paragraph reads as follows:

> This behavior suggests that the regularization radius ℓ can be interpreted as an effective length scale separating local fiber misalignment from the macroscopic myocardial organization contributing coherently to ventricular contraction. For small values of ℓ, smoothing primarily reduces microscopic disarray while preserving the physiological helical architecture, thereby promoting a more coordinated generation of active tension. Conversely, larger regularization radii progressively alter the macroscopic fiber organization itself, ultimately impairing mechanical efficiency. Interestingly, the optimal value iden-

tified in the present study ($\ell \approx 0.25\,\mathrm{mm}$) is substantially smaller than the ventricular wall thickness and is instead closer to the characteristic scale of small cardiomyocyte aggregates, supporting the interpretation of ℓ as a local microstructural length scale rather than a macroscopic geometric one. Under this interpretation, the optimal smoothing scale may depend only weakly on organ size and species, although dedicated investigations on larger mammalian hearts would be required to verify this hypothesis quantitatively.

Reviewer #2

> *The authors investigated the role of macroscopic fiber architecture and microscopic fiber disarray on cardiac electromechanical function. Accordingly, they performed electromechanical simulations in a murine biventricular model that included fiber orientation obtained with mesoscopic optical imaging. Results in this model were compared to those with rule-based approaches for assigning myocardial fiber orientation to image-based biventricular models. Interestingly, they found active mechanics to be sensitive to macroscopic fiber architecture, but not passive mechanics nor electrical activation maps. When macroscopic fiber architecture was preserved, they also found contraction to be stronger with less fiber disarray (either missing in rule-based methods or data overly smoothed).*
> *Overall, the results of this simulation improve our understanding for how electrical activation maps and ventricular mechanics are influenced by macroscopic and microscopic myocardial fiber architecture. In my opinion, this is a very challenging and important study to do. Although I have many questions and comments regarding the study, please see below, I found the study to provide valuable new information regarding the clinical relevance of macroscopic and microscopic fiber architecture in cardiac electromechanics. This knowledge will hopefully strengthen the ability of such models to more realistically simulate the mechanical behavior of the heart in patients.*

We sincerely thank the Reviewer for the thoughtful evaluation of the manuscript and for the valuable suggestions.

> *1. Line 146-148: "In particular, in electrophysiology models, fiber disarray is implicitly accounted for in the definition of the anisotropic diffusion tensor." In my opinion, this is also a resolution problem. At lower model resolutions > 150 um (resolution for the mesh in this study), I imagine much of the disarray is lost, which as mentioned, could be accounted for by adjusting the diffusion tensor in the directions transverse the longitudinal fiber direction. In models of lower resolution, would it still be possible to account for fiber disarray by adding noise to the to the longitudinal fiber direction? In theory, results for the distribution of this disarray could be derived from the optical imaging data from the study by the authors.*

We thank the Reviewer for this insightful observation. We agree that the treatment of fiber disarray is intrinsically related to spatial resolution. When the computational mesh does not resolve the characteristic length scale of local fiber variability, part of the disarray becomes a sub-grid feature and should therefore be incorporated through effective tissue properties, such as the transverse components of the anisotropic diffusion tensor. Conversely, at sufficiently high spatial resolution, local fiber perturbations could in principle be represented explicitly, either from direct measurements or through stochastic perturbations calibrated from high-resolution

imaging data, such as those employed in the present work. However, resolving such fine-scale variability in full-organ electromechanical simulations may rapidly become computationally prohibitive, which motivates the use of homogenized or effective representations in current large-scale cardiac models. We have clarified this point in the revised manuscript:

> This necessity is inherently resolution-dependent: when the computational mesh does not resolve local variations in fiber orientation, the effect of sub-grid fiber disarray should be incorporated through effective tissue properties. Conversely, at sufficiently high spatial resolution, local fiber perturbations may in principle be represented explicitly, either from direct measurements or through stochastic perturbations calibrated from high-resolution imaging data. However, resolving such fine-scale variability in full-organ electromechanical simulations may rapidly become computationally prohibitive, thereby motivating the use of homogenized representations in large-scale cardiac models.

> *2. Line 207: It is unclear why reference 50 is used here because previous studies have used nearly identical myocardial reference systems (according to the description given by the authors) well before reference 50's publication date in 2021. If reference 50 is doing something new, then please state so.*

We agree with the Reviewer. In the revised manuscript (Line 215), we added the references [1, 3, 17], particularly the work by Bayer et al. [1], which first introduced a myocardial reference system based on Laplace boundary-value problems.

> *3. Line 221: Is the intrusion angle the same as the imbrication angle used in early studies (https://doi.org/10.1093/europace/eum213)?*

We thank the Reviewer for suggesting reference [18]. We examined the similarities and differences between the angle γ (intrusion), as defined in our work, and the angle α_3, as defined in [18]. A one-to-one correspondence between the directions and planes used in the two formulations cannot be established, since our reference system relies on the local apico-basal direction, which varies pointwise within the myocardium, whereas [18] defines the longitudinal axis of the myocardium as a fixed direction over the whole domain. Nevertheless, the two reference systems are conceptually similar. Both γ and α_3 quantify the extent to which the fiber direction deviates from the tangential plane. However, they are defined in different planes. In our formulation, γ lies in the circumferential plane after the rotation associated with the helix angle α, whereas α_3 lies in the original circumferential plane, represented in Fig. 2 as the e_l–e_t plane.

4. Line 226: The Figure 1 legend does not seem to match the Figure 1 at the end of the manuscript. It seems that Figures 1 and 2 were swapped. Please correct. Secondly, regarding the current Figure 1, the apicobasal colorscale does a poor job of showing the apicobasal distance. Lastly, in Figure 1.C why are there not more et vectors observed? Did you postprocess the data to prevent this?

We thank the Reviewer for highlighting this mistake. We have renumbered Figures 1 and 2. In addition, we changed the colormap for the apico-basal coordinate and saturated it in the 0.9–1.0 range to better represent its variation across the domain; the caption has been changed accordingly. Finally, we changed the color of the e_t vectors and increased the transparency of the geometry in order to show the vectors entering the myocardium.

5. Line 229, to reiterate, reference 50 is not the first to introduce a myocardial reference frame. What makes it different from the reference frames used by the previous LDRBMs mentioned? The reference frame looks much like that in Figure 1 of doi: 10.1007/s10439-012-0593-5.

We thank the Reviewer for raising this point, which we acknowledge was not explained clearly enough in the original manuscript. Recently, in [12], we presented a unified framework for LDRBMs, providing a unified mathematical formulation for the existing approaches. The LDRBM mathematical framework of [12], is the framework adopted in the present work. We have clarified this point in the revised manuscript:

> Several LDRBMs have been proposed in the literature, each employing different strategies to compute the myocardial reference system [1, 3, 17]. Recently, a unified mathematical framework encompassing many of the existing LDRBM approaches has been proposed [12]. In this work, we use a LDRBM based on Doste et al. [3], with the modifications presented in [12], that enable its application to based biventricular geometries.

6. Line 230, why is reference 50 here if the method is from Doste el al (ref 52)? Secondly, your biventrciular models do not include outflow tracts, so why do you use the Doste method? From my understanding, the Doste method is just an extension of previous methods, and used these methods to incorporate local data. If you are not using the outflow track feature, or another novel feature that you specify from the Doste approach, then you should state the same method they based their algorithm on.

Following the points raised by the Reviewer, we clarified that the approach adopted in this work is based on the LDRBM originally proposed by Doste et al. [3], implemented within the unified framework presented in [12]. The unified framework introduced in [12] not only embeds the existing LDRBM approaches within a common mathematical formulation, but also incorporates some modeling improvements. In particular, we extended the Bayer et al. RBM [1] to include specific fiber orientations for the right ventricle (RV), and the Doste et al. RBM [3] to enable its application to based biventricular geometries.

Moreover, we also emphasized that the Doste et al. RBM is not merely an extension of previous methods including the outflow tracts, but also provides a dedicated treatment of specific ventricular regions, such as the inter-ventricular septum (approximately associating two-thirds of the septum to LV and one-third to RV) as well as tailored fiber orientations for the RV. All these aspects have now been clarified in the revised manuscript, as follows:

> In this work, we use a LDRBM based on Doste et al. [3], with the modifications presented in [12] that enables its application to based biventricular geometries. We emphasize that the Doste et al. RBM provides a dedicated treatment of specific ventricular regions, such as the inter-ventricular septum (approximately assigning two-thirds of the septum to LV and one-third to RV) as well as tailored fiber orientations for the RV.

8. Line 272: How does fiber architecture data from mesoscopic optical imaging compare to the traditionally used DTMRI?

We have not performed a systematic direct comparison between the fiber architecture quantified with our mesoscopic optical imaging approach and fiber architectures derived from DTMRI. However, in [5], we compared fiber reconstructions obtained from mesoSPIM microscopy with rule-based fiber organization models that were developed based on DTMRI datasets. Although indirect, this comparison demonstrated that the overall myocardial fiber organization identified by our method is consistent with the established DTMRI-derived framework. At the same time, the substantially higher spatial resolution of mesoSPIM provides additional structural details and local fiber features that cannot be resolved with conventional DTMRI. Therefore, while a direct validation against DTMRI was not part of the present study, the results from [5] support the consistency of the global fiber architecture recovered by the optical imaging approach while highlighting its ability to capture finer microstructural information.

9. Line 290: How does murine fiber architecture and disarray compare to large animal hearts (sheep, porcine, human)? What are the default parameter settings for the fiber angles in your rule-based methods?

We thank the Reviewer for pointing out this topic. We believe that the work in the literature that best addresses this question, although only partially, is [8], where left ventricular fiber fields obtained by diffusion tensor imaging were compared in mouse, rabbit, and sheep. Although the helical organization of myocardial fibers is preserved across species, the results show substantial interspecies differences in the helix-angle distribution. In particular, the mouse ventricle presents a larger transmural helix angle range and higher transmural helix-angle linearity compared with sheep and rabbit. In our opinion, this suggests that directly inferring fiber angles from other mammals, including humans, to the mouse, or vice versa, would be inappropriate. Therefore, for the LDRBM, we do not use literature values for the input angles; instead, we compute the modal values for the left and right epicardium and endocardium from our dataset. We thank the Reviewer for noting that these values were not reported. We have modified Table 1 of Appendix S1 to report the computed modal values for α and γ. To clarify this aspect we modified line 354 as follows

> ..., since the helix-angle distribution varies significantly accross species [8], we do not use literature values, but rather we compute the statistical distribution of α and γ selecting the modal value in different myocardial sub-regions, as done in [13] (see Fig. 5).

10. Line 342: It is difficult to see the fiber directions in Figure 3. Could you provide a zoomed in view of the transmural fiber orientation?

We thank the reviewer for the suggestion. We have modified Fig. 3 by increasing the density of the vectors rendered in the image and by adding a zoomed-in view of each fiber field to highlight variations in the transmural direction. We have revised the caption accordingly:

> **Comparison different fiber fields derived by varying the regularization radius ℓ with respect to the the unfiltered fiber field and the ldrbm one.** On the left the unfiltered fiber field and on the right the ldrbm one [3]. The first row shows the full geometry. The second row shows a magnified view of the region highlighted by the gray boxes in the first row, with an edge length of 3.0 mm. The colormap represents the angular variation of α within the myocardium.

11. Line 231: Do these scalar fields represent the actual distance from the boundary conditions or just a function between 0 and 1, which doesn't necessarily represent a physical distance. In other words, how are the fields computed? Distance, eikonal, Laplace, PDE-based approaches like in Cobiveco, etc.

We thank the Reviewer for pointing out this ambiguity. We clarify that ϕ and ψ are not distances, but rather the solutions of Laplace problems. Therefore, in Sec. 2.1.1, we have replaced the expressions "transmural distance" and "apico-basal distance" with "transmural coordinate" and "apico-basal coordinate", respectively. Furthermore, we have corrected lines 231–233 as follows:

> ... the transmural coordinate ϕ which surrogates the distance from the epicardium and the endocardial surfaces, see Fig 2(a); the apico-basal ψ coordinate representing the position with respect to the apex and the ventricular base, see Fig 2(b).

12. Lines 361-364: Isn't this what you are trying to study? As suggested by previous studies, this spatial variability in helical angle alpha is more likely to impact mechanics than electrical activation, with the impact on more difficult to study.

We thank the Reviewer for allowing us to clarify this aspect. The work of [7], suggests that decomposing the domain into a larger number of subregions and assigning a modal angle value to each of them would provide a better approximation of the original experimental fiber field. However, this approach is not representative of the state of the art, as in most models only epicardial and endocardial angles are prescribed. Therefore, our aim is not to investigate the fiber field within each subregion, but rather to assess the effect of local disarray, with respect to the mean fiber orientation, on cardiac electromechanics. We achieved this by introducing the fiber regularization algorithm. We agree that, as already suggested by [6, 7], active mechanics is more affected by fiber configuration than electrophysiology and passive mechanics. Nevertheless, given the interdependence of the model components, we preferred to investigate the effect on each component separately, rather than only on the fully coupled electromechanical model.

13. Line 366: In Figure 4 for the cross-sections at the base, why does the alpha angle invert from blue (endo) and green (epi) in the LV wall to red/green in the LV septum and blue in the RV septum?

We thank the Reviewer for noticing this important aspect related to septal fiber architecture. The fiber orientation within the septum (both in the portions associated with

LV and in those associated with RV) may differ substantially from that observed in the endo-epicardial regions of the LV/RV free walls, as also reported in [16]. In the lateral wall, fiber streamlines typically rotate smoothly from the subendocardial to the subepicardial layers. In contrast, within the septal wall, a discontinuity may be observed near the epicardial region, where fibers exhibit an inverse rotation from the antero-posterior direction to the inferior-superior direction (see, e.g., Figure 4 in [16]). We have now mentioned this aspect in Lines 375-380:

> In particular, the fiber orientation within the septum (both in the portions associated with the LV and in those associated with the RV) differs substantially from that observed in the endo-epicardial regions of the LV/RV free walls, in agreement with [16]. In the lateral wall, fiber orientation rotates smoothly from the endocardial to the epicardial layers. In contrast, within the septal wall, an inverse rotation from the antero-posterior direction to the inferior-superior direction is observed.

> *14. Line 416: Can the authors elaborate more on why they used these specific mechanical boundary conditions? Is this consistent with previous studies?*

We thank the Reviewer for requesting further clarification. Regarding the epicardium, Robin-type boundary conditions are commonly used to reproduce the effect of the pericardial sac on the myocardium [11]. This type of boundary condition has been previously applied in left-ventricular [15], biventricular [14], and whole-heart [9] electromechanical simulations. Robin boundary conditions are also applied at the base to surrogate the effect of the atria [4]. Furthermore, we would like to apologize for an error: at the endocardium, we use Neumann boundary conditions, as previously done in [14, 15], rather than "energy-consistent" boundary conditions, as incorrectly stated in the first draft. To clarify these aspects, we have modified the text in lines 416–420 as follows:

> Mechanical boundary conditions are imposed in four cardiac zones: epicardium, left and right endocardium, and the ventricular base. Robin-type boundary conditions are applied at the epicardium and basal plane, following the approach in [11]. These boundary conditions introduce stiffness and damping parameters that reproduce the interaction between the myocardium and the pericardium. This interaction is modeled as springs and dashpots acting in the normal and tangential directions with respect to the epicardium. At the base, less stiff Robin boundary conditions are applied to reproduce the ef-

> fect of the atria on the ventricles. At the endocardium, the mechanical model is coupled with the blood circulation model through Neumann boundary conditions accounting for the pressure exerted by the blood inside the chamber.

> *15. Line 433: What does element size mean? The average edge length of a tetrahedral element? Also, 150 microns seems like a very high-resolution for simulating cardiac mechanics. Are these simulations very computationally demanding? For a much larger human heart (more mesh nodes), would similar simulations be possible at this same spatial mesh resolution?*

We thank the Reviewer for pointing out this aspect. The value of 150 µm actually refers to the average edge length. We therefore replaced the expression "element size" with "edge length" at line 433. These simulations, run on 48 physical cores, require around 5 hours; therefore, maintaining the same resolution at the human scale would be computationally demanding.

> *16. Lines 460-467: This paragraph seems more appropriate for the limitations section.*

We agree that part of the original paragraph contained considerations more closely related to the interpretation and limitations of the study. Accordingly, we revised the manuscript by shortening the paragraph in Sec. 4.3 so that it now only provides the physiological motivation for focusing the electromechanical analysis on the LV:

> In the murine heart, global mechanical function is predominantly governed by the LV, whereas the RV operates under substantially lower pressure and mechanical load due to the low-resistance pulmonary circulation [10]. Moreover, owing to its extremely thin free wall and strong ventricular interdependence, RV dynamics are largely driven by LV contraction. Accordingly, in the following electromechanical analysis we primarily focus on the LV.

At the same time, we moved the discussion regarding the limitations associated with RV analysis to the Discussion section:

> In addition, the present analysis focused primarily on the LV. In murine hearts, RV mechanics are strongly influenced by ventricular interdependence and by the substantially lower pressure regime of the pulmonary circulation. Moreover, the thin RV free wall also results in a lower signal-to-noise ratio in the experimental fiber measurements. As a consequence, the conclusions drawn here regarding electrome-

chanical function are more directly supported for the LV than for the RV.

17. Line 467: In Figure 6, the isolines are 1 ms apart. Without the spatial dimensions of the tissue sample provided along side the activation time data, activation seems very rapid. Please add the spatial dimensions in text or a scale bar to the figure. Secondly, what is the conduction velocity of these activation maps along the three fiber directions? Does this change with smoothed disarray?

We thank the Reviewer for pointing out this weakness. To address this comment, we modified Figure 6 to report the total activation time for each case and added Figure S12 to the Supporting Information, where the total activation time is plotted as a function of the regularization radius. In addition, in Table 1 of the Supporting Information, we now report the theoretical conduction velocities used in the eikonal problem, namely $v_f = 0.777\,\mathrm{m\,s^{-1}}$ in the longitudinal direction and $v_s = v_n = 0.549\,\mathrm{m\,s^{-1}}$ in the sheetlet and normal directions, which were computed according to the formula $v_f = c_0\sqrt{\sigma_f}$, as in [13]. Therefore, the theoretical conduction velocity is independent of fiber disarray; however, the total activation time is partially affected by the tissue anisotropy and, consequently, by fiber disarray, while being more strongly influenced by the mean fiber direction, as shown in Figure 6. Indeed, for $\ell = 1.0\,\mathrm{mm}$, not only the fiber disarray but also the local mean fiber direction changes, thereby affecting the total activation time, which increases from 11.78 ms in the baseline case to 12.60 ms in the regularized case with $\ell = 1.0\,\mathrm{mm}$.

18. Line 477: Some quantification would help. Most of this section is very qualitative.

We agree with the Reviewer that a more quantitative analysis is needed. To address this point, we further investigated the differences between the regularized and rule-based fiber fields by comparing the total activation times reported in Figure 6:

> Fig. 6 shows the endocardial and epicardial activation maps together with the total activation time $T_{\max}$, for increasing fiber regularization radius and for the ldrbm. The results for the baseline case are aligned with the literature [2]. Wavefronts originating from the stimulation sites propagate preferentially along the higher-conductivity fiber direction. As expected, in the experimental fiber field, the presence of disarray reduced the directional effect of faster conduction along the fibers, resulting in a more isotropic propagation pattern compared to ldrbm fibers. However filtering out the disarray results only in slight

reduction of $T_{\max}$ for $\ell \leq 0.25$ mm. Moreover, as the fiber regularization radius increases, the preferred direction of propagation in the longitudinal direction becomes more pronounced. Overall, the ldrbm produces an activation pattern similar to that of the regularized fiber field and its $T_{\max}$ is in between the total activation time of the case with $\ell = 0.5$ mm and the case with $\ell = 1.0$ mm, suggesting that ldrbm captures the mean fiber orientation. However, signal propagation with ldrbm is more anisotropic compared to the experimental fiber field. $T_{\max}$ shows therefore a weak dependence on the regularization radius as it varies only between 11.67 ms and 11.98 ms when $\ell \leq 0.5$ mm. To observe a meaningful change in $T_{\max}$, the regularization must be increased to 1.0 mm.

19. Line 516: I was under the impression that small changes in fiber architecture had little impact on activation patterns in your model. Are you discussing macroscopic fiber architecture here? Also, did the authors consider comparing their results to simulations with uniform fiber orientation (all vectors pointing in the direction of the endo-epi or apex-base axis), or no fiber orientation (the same conductivity values in all three fiber directions)?

We thank the Reviewer for this observation. Here the statement was intended in a modeling sense: the fiber directions explicitly enter the constitutive formulations of electrophysiology, passive mechanics, and active contraction, and therefore influence all components of the electromechanical model. The actual sensitivity of the resulting quantities of interest to fiber architecture is then quantified in the subsequent sections.

We did not consider simulations with uniform fiber orientation because such configurations would lead to strongly non-physiological contraction patterns, preventing an efficient ventricular squeezing mechanism and therefore producing unrealistically low pressure generation. On the other hand, fully isotropic conductivity tensors would by construction suppress the influence of fiber orientation on electrical propagation. However, meaningful comparison in this case would require defining an effective isotropic conductivity value intermediate between the longitudinal and transverse conductivities. Since the specific choice of this effective parameter would itself strongly affect activation times and propagation patterns, the interpretation of the resulting differences would become challenging.

20. Line 524: The changes in each graph of Figure 8 appear to be very small with respect to the x-axis l parameter. Are any of these changes considered clinically significant?

We thank the Reviewer for this observation. The LV peak pressure varies from approximately 90 to 96 mmHg, corresponding to a relative change of about 6.7%,

whereas the EF varies from approximately 45% to 52%. While we would avoid referring to these differences as clinically significant, since the present study is based on a murine computational model rather than on clinical outcome data, they are for sure mechanically meaningful. In particular, the EF variation indicates a non-negligible change in pumping efficiency induced solely by modifications of fiber organization/disarray, with all other model parameters kept fixed.

> *20. Line 578: This sentence does not make sense.*

We agree that the original sentence was unclear and have therefore rephrased it to improve readability:

> We then investigate whether the proposed surrogate strategies for fiber disarray (i.e., variations of the Stress Factors (SF) parameters, see Eq. 20) are able to reproduce the functional consequences of the experimentally observed disordered architecture.

> *21. Lines 619-628: This seems more like methods than results.*

We thank the Reviewer for the suggestion. We moved lines 619–628 to a new Section 3.1, titled "Strain computation," within the section Materials and Methods.

> *22. Line 749: Although you have data at 96 um, it ends up being downsampled to the mesh resolution of 150um correct? If this is the case, it is not clear to me how important this statement is.*

We thank the Reviewer for pointing out this aspect. The average edge length of 150 µm results from the meshing procedure, wherein the rationale was to have a minimum cell diameter compatible with the data resolution. The result was a mesh with minimum cell diameter of 91 µm and average one of 150 µm. We modified line 433 to clarify this aspect:

> The multiphysics problem is discretized on a tetrahedral mesh composed of 328 000 nodes, with an averege edge length of 150 µm and a minimum cell diameter of 91 µm. We selected the minimum mesh size to capture the fiber field variations without unnecessarily increasing the computational cost.

23. Line 770: What needs to be done to make this possible? Is it computational resources or proper data, or both, the reason why everyone is not doing this now?

We thank the Reviewer for this important question. The main current limitation is the lack of suitable experimental data. Whole-heart microstructural datasets with the spatial resolution employed in the present study have only become available very recently, thanks to advances in mesoscopic optical imaging techniques. At present, these approaches are still largely limited to relatively small specimens, such as murine hearts, and their extension to substantially larger organs remains challenging. For example, acquiring a full human heart dataset at comparable resolution is currently not feasible in practice. Computational cost also represents an important challenge for fully coupled electromechanical simulations at this level of structural detail, although we believe that the primary bottleneck at present remains the availability of suitable high-resolution experimental datasets. We have now mentioned this aspect in the Discussion:

> While direct quantitative translation to the human heart is not straightforward due to well-known inter-species differences in structure and function, the computational framework developed here is species-agnostic and may provide a basis for future investigations in larger animal models and, eventually, in human-specific geometries, although the broader adoption of such approaches is currently limited by the availability of suitable high-resolution whole-heart datasets for large mammalian hearts.

References

[1] J. D. Bayer, R. C. Blake, G. Plank, and N. A. Trayanova. A novel rule-based algorithm for assigning myocardial fiber orientation to computational heart models. *Annals of biomedical engineering*, 40(10):2243–2254, 2012.

[2] C. Crocini, C. Ferrantini, R. Coppini, M. Scardigli, P. Yan, L. M. Loew, G. Smith, E. Cerbai, C. Poggesi, F. S. Pavone, and L. Sacconi. Optogenetics design of mechanistically-based stimulation patterns for cardiac defibrillation. *Scientific reports*, 6(1):35628, 2016.

[3] R. Doste, D. Soto-Iglesias, G. Bernardino, A. Alcaine, R. Sebastian, S. Giffard-Roisin, M. Sermesant, A. Berruezo, D. Sanchez-Quintana, and O. Camara. A rule-based method to model myocardial fiber orientation in cardiac biventricular geometries with outflow tracts. *International journal for numerical methods in biomedical engineering*, 35(4):e3185, 2019.

[4] A. Gerbi, L. Dedè, and A. Quarteroni. A monolithic algorithm for the simulation of cardiac electromechanics in the human left ventricle. 2018.

[5] F. Giardini, C. Olianti, G. A. Marchal, F. Campos, V. Romanelli, J. Steyer, J. Madl, R. Piersanti, G. Arecchi, I. Perumal Vanaja, et al. Correlative imaging integrates electrophysiology with three-dimensional murine heart reconstruction to reveal electrical coupling between cell types. *Nature Cardiovascular Research*, 4(11):1466–1486, 2025.

[6] D. Gil, R. Aris, A. Borras, E. Ramírez, R. Sebastian, and M. Vazquez. Influence of fiber connectivity in simulations of cardiac biomechanics. *International journal of computer assisted radiology and surgery*, 14(1):63–72, 2019.

[7] D. Guan, J. Yao, X. Luo, and H. Gao. Effect of myofibre architecture on ventricular pump function by using a neonatal porcine heart model: from dt-mri to rule-based methods. *Royal Society open science*, 7(4):191655, 2020.

[8] L. J. Healy, Y. Jiang, and E. W. Hsu. Quantitative comparison of myocardial fiber structure between mice, rabbit, and sheep using diffusion tensor cardiovascular magnetic resonance. *Journal of Cardiovascular Magnetic Resonance*, 13(1):74, 2011.

[9] J. Krauß, T. Gerach, and A. Loewe. Comparison of pericardium modeling approaches for mechanical whole heart simulations. In *2023 Computing in Cardiology (CinC)*, volume 50, pages 1–4. IEEE, 2023.

[10] N. Milani-Nejad and P. M. Janssen. Small and large animal models in cardiac contraction research: advantages and disadvantages. *Pharmacology & therapeutics*, 141(3):235–249, 2014.

[11] M. R. Pfaller, J. M. Hörmann, M. Weigl, A. Nagler, R. Chabiniok, C. Bertoglio, and W. A. Wall. The importance of the pericardium for cardiac biomechanics: from physiology to computational modeling. *Biomechanics and modeling in mechanobiology*, 18(2):503–529, 2019.

[12] R. Piersanti, P. C. Africa, M. Fedele, C. Vergara, L. Dedè, A. F. Corno, and A. Quarteroni. Modeling cardiac muscle fibers in ventricular and atrial electrophysiology simulations. *Computer Methods in Applied Mechanics and Engineering*, 373:113468, 2021.

[13] R. Piersanti, R. Bradley, S. Y. Ali, A. Quarteroni, L. Dede', and N. A. Trayanova. Defining myocardial fiber bundle architecture in atrial digital twins. *Computers in biology and medicine*, 188:109774, 2025.

[14] R. Piersanti, F. Regazzoni, M. Salvador, A. F. Corno, L. Dede', C. Vergara, and A. Quarteroni. 3d–0d closed-loop model for the simulation of cardiac biventricular electromechanics. *Computer Methods in Applied Mechanics and Engineering*, 391:114607, 2022.

[15] F. Regazzoni, M. Salvador, P. C. Africa, M. Fedele, L. Dedè, and A. Quarteroni. A cardiac electromechanical model coupled with a lumped-parameter model for closed-loop blood circulation. *Journal of Computational Physics*, 457:111083, 2022.

[16] J. Rodríguez-Padilla, A. Petras, J. Magat, J. Bayer, Y. Bihan-Poudec, D. El Hamrani, G. Ramlugun, A. Neic, C. M. Augustin, F. Vaillant, et al. Impact of intraventricular septal fiber orientation on cardiac electromechanical function. *American Journal of Physiology-Heart and Circulatory Physiology*, 322(6):H936–H952, 2022.

[17] S. Rossi, T. Lassila, R. Ruiz-Baier, A. Sequeira, and A. Quarteroni. Thermodynamically consistent orthotropic activation model capturing ventricular systolic wall thickening in cardiac electromechanics. *European Journal of Mechanics-A/Solids*, 48:129–142, 2014.

[18] D. L. Weiss, D. U. Keller, G. Seemann, and O. Dössel. The influence of fibre orientation, extracted from different segments of the human left ventricle, on the activation and repolarization sequence: a simulation study. *Europace*, 9(suppl_6):vi96–vi104, 2007.
