# Supplementary material for: The functional impact of myofiber macroscopic organization and disarray in computational models of the murine heart
Source: PLoS Comput Biol. 2026 Sep 21;22(9):e1014807. doi: 10.1371/journal.pcbi.1014807 (PMC13614661; doi:10.1371/journal.pcbi.1014807)
Supplement: Supplementary file 5 [file pcbi.1014807.s014.pdf]

September 3, 2026

The functional impact of myofiber macroscopic organization and disarray in computational models of the murine heart
PLOS Computational Biology - Manuscript Number PCOMPBIOL-D-26-00721

Answers to Reviewers - Second Review

Dear Editor,

We thank the Reviewers for carefully reading our paper and our response. Hereafter we address the remaining questions. The pages and references mentioned in our answers refer to the revised manuscript. The modifications in the paper are highlighted in blue.

With our best regards,

Carlo Guastamacchia, Roberto Piersanti, Francesco Giardini, Raffaele Coppini, Cecilia Ferrantini, Luca Dede', Leonardo Sacconi, Francesco Regazzoni

Reviewer #1

> *I recommend this manuscript for acceptance in its current form.*

We are grateful to the Reviewer for carefully reading the reviewed manuscript and for recommending it for acceptance.

Reviewer #2

> *The authors have addressed the majority of my comments and concerns. Bravo. I only have two very minor requests remaining.*

We thank the Reviewer for reading our manuscript and response. Following we answer to the remaining requests.

> *1. Define what "based biventricular geometries" means.*

We thank the Reviewer for highlightining this ambiguity. We clarified the meaning of "based biventricular geometries" in the manuscript at line 241:

> In this work, we use a LDRBM based on Doste et al. [52], with the modifications presented in [50], that enable its application to based biventricular geometries. Here, based refers to the artificial truncation of the ventricular geometry by a basal plane, introduced to define the ventricular base as a boundary of the computational domain.

2 Include the computation times required to run your simulations.

We thank the Reviewer for rising this point. To address it we provided an average time value for each hearbeat, in the manuscript at line 471:

> The simulations were run on one CPU node powered by two 24-core CPUs with 512 GB RAM and required approximately 2000 s to reproduce one heartbeat.
