# Supplementary material for: The functional impact of myofiber macroscopic organization and disarray in computational models of the murine heart
Source: PLoS Comput Biol. 2026 Sep 21;22(9):e1014807. doi: 10.1371/journal.pcbi.1014807 (PMC13614661; doi:10.1371/journal.pcbi.1014807)
Supplement: Peer review history

# Peer review: Decision Letter 0

**DOI:** 10.1371/journal.pcbi.1014807.r001

**Authors:** Stacey D. Finley, Haibo Ni

20 May 2026

The functional impact of myofiber macroscopic organization and disarray in computational models of the murine heart

PLOS Computational Biology

Dear Dr. Regazzoni,

Thank you for submitting your manuscript to PLOS Computational Biology. After careful consideration, we feel that it has merit but does not fully meet PLOS Computational Biology's publication criteria as it currently stands. Therefore, we invite you to submit a revised version of the manuscript that addresses the points raised during the review process.

Please submit your revised manuscript by Jul 20 2026 11:59PM. If you will need more time than this to complete your revisions, please reply to this message or contact the journal office at ploscompbiol@plos.org. When you're ready to submit your revision, log on to https://www.editorialmanager.com/pcompbiol/ and select the 'Submissions Needing Revision' folder to locate your manuscript file.

Please include the following items when submitting your revised manuscript:

* A letter that responds to each point raised by the editor and reviewer(s). You should upload this letter as a separate file labeled 'Response to Reviewers'. This file does not need to include responses to formatting updates and technical items listed in the 'Journal Requirements' section below.

* A marked-up copy of your manuscript that highlights changes made to the original version. You should upload this as a separate file labeled 'Revised Manuscript with Track Changes'.

* An unmarked version of your revised paper without tracked changes. You should upload this as a separate file labeled 'Manuscript'.

As the corresponding author, your ORCID iD is verified in the submission system and will appear in the published article. PLOS supports the use of ORCID, and we encourage all coauthors to register for an ORCID iD and use it as well. Please encourage your coauthors to verify their ORCID iD within the submission system before final acceptance, as unverified ORCID iDs will not appear in the published article. Only the individual author can complete the verification step; PLOS staff cannot verify ORCID iDs on behalf of authors.

We look forward to receiving your revised manuscript.

Kind regards,

Haibo Ni

Academic Editor

PLOS Computational Biology

Stacey Finley

Section Editor

PLOS Computational Biology

**Additional Editor Comments:**

Both reviewers agree upon the challenges and significance of the study. However, a number of concerns and suggestions have been raised, and should be addressed.

**Journal Requirements:**

If the reviewer comments include a recommendation to cite specific previously published works, please review and evaluate these publications to determine whether they are relevant and should be cited. There is no requirement to cite these works unless the editor has indicated otherwise.

1) Please ensure that the CRediT author contributions listed for every co-author are completed accurately and in full.

At this stage, the following Authors/Authors require contributions: Cecilia Ferrantini. Please ensure that the full contributions of each author are acknowledged in the "Add/Edit/Remove Authors" section of our submission form.

The list of CRediT author contributions may be found here: https://journals.plos.org/ploscompbiol/s/authorship#loc-author-contributions

2) We ask that a manuscript source file is provided at Revision. Please upload your manuscript file as a .doc, .docx, .rtf or .tex. If you are providing a .tex file, please upload it under the item type u2018LaTeX Source Fileu2019 and leave your .pdf version as the item type u2018Manuscriptu2019.

3) Your manuscript is missing the following sections: Methods.  Please ensure all required sections are present and in the correct order. Make sure section heading levels are clearly indicated in the manuscript text, and limit sub-sections to 3 heading levels. An outline of the required sections can be consulted in our submission guidelines here:

https://journals.plos.org/ploscompbiol/s/submission-guidelines#loc-parts-of-a-submission

4) We have noticed that you have cited Table  5 in the manuscript file but there is no corresponding table in the manuscript.  Please amend your manuscript to include this table noting that tables should not be uploaded as individual files.

5) In the online submission form, you indicated that your data will be submitted to a repository upon acceptance. We strongly recommend all authors deposit their data before acceptance, as the process can be lengthy and hold up publication timelines. Please note that, though access restrictions are acceptable now, your entire minimal dataset will need to be made freely accessible if your manuscript is accepted for publication. This policy applies to all data except where public deposition would breach compliance with the protocol approved by your research ethics board. If you are unable to adhere to our open data policy, please kindly revise your statement to explain your reasoning and we will seek the editor's input on an exemption.

6) Please amend your detailed Financial Disclosure statement. This is published with the article. It must therefore be completed in full sentences and contain the exact wording you wish to be published.

1) State what role the funders took in the study. If the funders had no role in your study, please state: "The funders had no role in study design, data collection and analysis, decision to publish, or preparation of the manuscript."

2) If any authors received a salary from any of your funders, please state which authors and which funders..

If you did not receive any funding for this study, please simply state: u201cThe authors received no specific funding for this work.u201d

7)  Please ensure that the funders and grant numbers match between the Financial Disclosure field and the Funding Information tab in your submission form. Note that the funders must be provided in the same order in both places as well.

**Reviewers' comments:**

Reviewer's Responses to Questions

**Comments to the Authors:**

**Please note here if the review is uploaded as an attachment.**

Reviewer #1: The manuscript titled "The functional impact of myofiber macroscopic organization and disarray in computational models of the murine heart" presents an exceptional study that leverages high-resolution optical imaging data to decouple macroscopic fiber architecture from local disarray. This work addresses a fundamental and long-standing limitation in cardiac digital twins—the reconstruction of fiber fields. The introduction of the Helmholtz spatial filtering strategy is novel and provides critical insights into the limitations of widely used Rule-Based Methods (LDRBM) and cross-fiber active stress surrogates.

The paper is well-written, the methodology is rigorous, and the findings are of significant importance to the computational cardiology community. The results convincingly demonstrate that global electrophysiology and passive mechanics are less sensitive to disarray compared to active contraction, and that a reduction in effective contractility is a more accurate phenomenological surrogate than orthogonal stress components.

I recommend Minor Revision to address the following two points, which primarily require clarification and extended discussion in the text rather than new experiments.

1. Generalizability and Single-Sample Limitations

The study is based on a single high-resolution ex vivo murine heart dataset, which is used as the reference ground truth. This is a valuable dataset, but it would be helpful if the authors could discuss more explicitly the extent to which the conclusions may depend on this specific specimen. In particular, inter-subject variability, preparation-related effects, imaging uncertainty, and the specific structural features of the murine heart may influence the relative roles of macroscopic fiber organization and microscopic disarray reported here. A clearer discussion of these limitations in the Discussion section, particularly regarding the generalizability to larger mammalian hearts, would improve the manuscript.

2. Physical Interpretation of the Optimal Smoothing Parameter (ℓ)

The authors have identified an optimal parameter value (ℓ = 0.25 mm), but the manuscript does not currently elaborate on why this particular value emerges. Is it because it corresponds to roughly one-quarter of the murine wall thickness (~1 mm)? Does it reflect the characteristic width of a few cardiomyocytes? Or is it an empirical optimum specific to this dataset? Given that the human ventricular wall is approximately 10–15 mm thick, this raises an important question for readers who may wish to apply this method to other species: Should the optimal ℓ be scaled proportionally (e.g., to 2.5–3.75 mm for humans), or should it be treated as an absolute constant linked to a species-invariant cellular length scale (~100 μm)? I encourage the authors to include a short discussion in the manuscript addressing this point to enhance the methodological clarity and cross-species applicability of their framework.

Reviewer #2: The authors investigated the role of macroscopic fiber architecture and microscopic fiber disarray on cardiac electromechanical function. Accordingly, they performed electromechanical simulations in a murine biventricular model that included fiber orientation obtained with mesoscopic optical imaging. Results in this model were compared to those with rule-based approaches for assigning myocardial fiber orientation to image-based biventricular models. Interestingly, they found active mechanics to be sensitive to macroscopic fiber architecture, but not passive mechanics nor electrical activation maps. When macroscopic fiber architecture was preserved, they also found contraction to be stronger with less fiber disarray (either missing in rule-based methods or data overly smoothed).

Overall, the results of this simulation improve our understanding for how electrical activation maps and ventricular mechanics are influenced by macroscopic and microscopic myocardial fiber architecture. In my opinion, this is a very challenging and important study to do. Although I have many questions and comments regarding the study, please see below, I found the study to provide valuable new information regarding the clinical relevance of macroscopic and microscopic fiber architecture in cardiac electromechanics. This knowledge will hopefully strengthen the ability of such models to more realistically simulate the mechanical behavior of the heart in patients.

Line 146-148: "In particular, in electrophysiology models, fiber disarray is implicitly accounted for in the definition of the anisotropic diffusion tensor." In my opinion, this is also a resolution problem. At lower model resolutions > 150 um (resolution for the mesh in this study), I imagine much of the disarray is lost, which as mentioned, could be accounted for by adjusting the diffusion tensor in the directions transverse the longitudinal fiber direction. In models of lower resolution, would it still be possible to account for fiber disarray by adding noise to the to the longitudinal fiber direction? In theory, results for the distribution of this disarray could be derived from the optical imaging data from the study by the authors.

Line 207: It is unclear why reference 50 is used here because previous studies have used nearly identical myocardial reference systems (according to the description given by the authors) well before reference 50's publication date in 2021. If reference 50 is doing something new, then please state so.

Line 221: Is the intrusion angle the same as the imbrication angle used in early studies (https://doi.org/10.1093/europace/eum213)?

Line 226: The Figure 1 legend does not seem to match the Figure 1 at the end of the manuscript. It seems that Figures 1 and 2 were swapped. Please correct. Secondly, regarding the current Figure 1, the apicobasal colorscale does a poor job of showing the apicobasal distance. Lastly, in Figure 1.C why are there not more et vectors observed? Did you postprocess the data to prevent this?

Line 229, to reiterate, reference 50 is not the first to introduce a myocardial reference frame. What makes it different from the reference frames used by the previous LDRBMs mentioned? The reference frame looks much like that in Figure 1 of doi: 10.1007/s10439-012-0593-5.

Line 230, why is reference 50 here if the method is from Doste el al (ref 52)? Secondly, your biventrciular models do not include outflow tracts, so why do you use the Doste method? From my understanding, the Doste method is just an extension of previous methods, and used these methods to incorporate local data. If you are not using the outflow track feature, or another novel feature that you specify from the Doste approach, then you should state the same method they based their algorithm on.

Line 231: Do these scalar fields represent the actual distance from the boundary conditions or just a function between 0 and 1, which doesn't necessarily represent a physical distance. In other words, how are the fields computed? Distance, eikonal, Laplace, PDE-based approaches like in Cobiveco, etc.

Line 272: How does fiber architecture data from mesoscopic optical imaging compare to the traditionally used DTMRI?

Line 290: How does murine fiber architecture and disarray compare to large animal hearts (sheep, porcine, human)? What are the default parameter settings for the fiber angles in your rule-based methods?

Line 342: It is difficult to see the fiber directions in Figure 3. Could you provide a zoomed in view of the transmural fiber orientation?

Lines 361-364: Isn't this what you are trying to study? As suggested by previous studies, this spatial variability in helical angle alpha is more likely to impact mechanics than electrical activation, with the impact on more difficult to study.

Line 366: In Figure 4 for the cross-sections at the base, why does the alpha angle invert from blue (endo) and green (epi) in the LV wall to red/green in the LV septum and blue in the RV septum?

Line 416: Can the authors elaborate more on why they used these specific mechanical boundary conditions? Is this consistent with previous studies?

Line 433: What does element size mean? The average edge length of a tetrahedral element? Also, 150 microns seems like a very high-resolution for simulating cardiac mechanics. Are these simulations very computationally demanding? For a much larger human heart (more mesh nodes), would similar simulations be possible at this same spatial mesh resolution?

Lines 460-467: This paragraph seems more appropriate for the limitations section.

Line 467: In Figure 6, the isolines are 1 ms apart. Without the spatial dimensions of the tissue sample provided along side the activation time data, activation seems very rapid. Please add the spatial dimensions in text or a scale bar to the figure. Secondly, what is the conduction velocity of these activation maps along the three fiber directions? Does this change with smoothed disarray?

Line 477: Some quantification would help. Most of this section is very qualitative.

Line 516: I was under the impression that small changes in fiber architecture had little impact on activation patterns in your model. Are you discussing macroscopic fiber architecture here? Also, did the authors consider comparing their results to simulations with uniform fiber orientation (all vectors pointing in the direction of the endo-epi or apex-base axis), or no fiber orientation (the same conductivity values in all three fiber directions)?

Line 524: The changes in each graph of Figure 8 appear to be very small with respect to the x-axis l parameter. Are any of these changes considered clinically significant?

Line 578: This sentence does not make sense.

Lines 619-628: This seems more like methods than results.

Line 749: Although you have data at 96 um, it ends up being downsampled to the mesh resolution of 150um correct? If this is the case, it is not clear to me how important this statement is.

Line 770: What needs to be done to make this possible? Is it computational resources or proper data, or both, the reason why everyone is not doing this now?

**********

**Have the authors made all data and (if applicable) computational code underlying the findings in their manuscript fully available?**

The PLOS Data policy requires authors to make all data and code underlying the findings described in their manuscript fully available without restriction, with rare exception (please refer to the Data Availability Statement in the manuscript PDF file). The data and code should be provided as part of the manuscript or its supporting information, or deposited to a public repository. For example, in addition to summary statistics, the data points behind means, medians and variance measures should be available. If there are restrictions on publicly sharing data or code —e.g. participant privacy or use of data from a third party—those must be specified.

Reviewer #1: Yes

Reviewer #2: Yes

**********

PLOS authors have the option to publish the peer review history of their article (what does this mean?). If published, this will include your full peer review and any attached files.

If you choose “no”, your identity will remain anonymous but your review may still be made public.

**Do you want your identity to be public for this peer review?**  For information about this choice, including consent withdrawal, please see our Privacy Policy.

Reviewer #1: **Yes:**  Teng-Chao Li

Reviewer #2: No

**Figure resubmission:**

After uploading your figures to PLOS’s NAAS tool - https://ngplosjournals.pagemajik.ai/artanalysis, NAAS will process the files provided and display the results in the "Uploaded Files" section of the page as the processing is complete. If the uploaded figures meet our requirements (or NAAS is able to fix the files to meet our requirements), the figure will be marked as "fixed" above. If NAAS is unable to fix the files, a red "failed" label will appear above. When NAAS has confirmed that the figure files meet our requirements, please download the file via the download option, and include these NAAS processed figure files when submitting your revised manuscript.

**Reproducibility:**

To enhance the reproducibility of your results, we recommend that authors of applicable studies deposit laboratory protocols in protocols.io, where a protocol can be assigned its own identifier (DOI) such that it can be cited independently in the future. Additionally, PLOS ONE offers an option to publish peer-reviewed clinical study protocols. Read more information on sharing protocols at https://plos.org/protocols?utm_medium=editorial-email&utm_source=authorletters&utm_campaign=protocols

# Author comment: Author response to Decision Letter 1

**DOI:** 10.1371/journal.pcbi.1014807.r002

6 Jul 2026

Attachment Submitted filename: fibers_article__PLOS___Review_.pdf

# Peer review: Decision Letter 1

**DOI:** 10.1371/journal.pcbi.1014807.r003

**Authors:** Stacey D. Finley, Haibo Ni

11 Aug 2026

PCOMPBIOL-D-26-00721R1

The functional impact of myofiber macroscopic organization and disarray in computational models of the murine heart

PLOS Computational Biology

Dear Dr. Regazzoni,

Thank you for submitting your manuscript to PLOS Computational Biology. After careful consideration, we feel that it has merit but does not fully meet PLOS Computational Biology's publication criteria as it currently stands. Therefore, we invite you to submit a revised version of the manuscript that addresses the points raised during the review process.

Please submit your revised manuscript by Oct 11 2026 11:59PM. If you will need more time than this to complete your revisions, please reply to this message or contact the journal office at ploscompbiol@plos.org. When you're ready to submit your revision, log on to https://www.editorialmanager.com/pcompbiol/ and select the 'Submissions Needing Revision' folder to locate your manuscript file.

Please include the following items when submitting your revised manuscript:

* A letter that responds to each point raised by the editor and reviewer(s). You should upload this letter as a separate file labeled 'Response to Reviewers'. This file does not need to include responses to formatting updates and technical items listed in the 'Journal Requirements' section below.

* A marked-up copy of your manuscript that highlights changes made to the original version. You should upload this as a separate file labeled 'Revised Manuscript with Track Changes'.

* An unmarked version of your revised paper without tracked changes. You should upload this as a separate file labeled 'Manuscript'.

If you would like to make changes to your financial disclosure, competing interests statement, or data availability statement, please make these updates within the submission form at the time of resubmission. Guidelines for resubmitting your figure files are available below the reviewer comments at the end of this letter.

As the corresponding author, your ORCID iD is verified in the submission system and will appear in the published article. PLOS supports the use of ORCID, and we encourage all coauthors to register for an ORCID iD and use it as well. Please encourage your coauthors to verify their ORCID iD within the submission system before final acceptance, as unverified ORCID iDs will not appear in the published article. Only the individual author can complete the verification step; PLOS staff cannot verify ORCID iDs on behalf of authors.

We look forward to receiving your revised manuscript.

Kind regards,

Haibo Ni

Academic Editor

PLOS Computational Biology

Stacey Finley

Section Editor

PLOS Computational Biology

**Additional Editor Comments (if provided):**

The authors have thoroughly addressed the reviewers' comments and suggestions, with only minor edits required:

"1.) Define what "based biventricular geometries" means.

2.) Include the computation times required to run your simulations."

**Reviewers' comments:**

Reviewer's Responses to Questions

**Comments to the Authors:**

**Please note here if the review is uploaded as an attachment.**

Reviewer #1: I recommend this manuscript for acceptance in its current form.

Reviewer #2: The authors have addressed the majority of my comments and concerns. Bravo.

I only have two very minor requests remaining.

1.) Define what "based biventricular geometries" means.

2.) Include the computation times required to run your simulations.

**********

**Have the authors made all data and (if applicable) computational code underlying the findings in their manuscript fully available?**

The PLOS Data policy requires authors to make all data and code underlying the findings described in their manuscript fully available without restriction, with rare exception (please refer to the Data Availability Statement in the manuscript PDF file). The data and code should be provided as part of the manuscript or its supporting information, or deposited to a public repository. For example, in addition to summary statistics, the data points behind means, medians and variance measures should be available. If there are restrictions on publicly sharing data or code —e.g. participant privacy or use of data from a third party—those must be specified.

Reviewer #1: None

Reviewer #2: Yes

**********

PLOS authors have the option to publish the peer review history of their article (what does this mean?). If published, this will include your full peer review and any attached files.

If you choose “no”, your identity will remain anonymous but your review may still be made public.

**Do you want your identity to be public for this peer review?**  For information about this choice, including consent withdrawal, please see our Privacy Policy.

Reviewer #1: **Yes:**  Teng-Chao Li

Reviewer #2: No

[NOTE: If reviewer comments were submitted as an attachment file, they will be attached to this email and accessible via the submission site. Please log into your account, locate the manuscript record, and check for the action link "View Attachments". If this link does not appear, there are no attachment files.]

**Figure resubmission:**

**Reproducibility:**

To enhance the reproducibility of your results, we recommend that authors of applicable studies deposit laboratory protocols in protocols.io, where a protocol can be assigned its own identifier (DOI) such that it can be cited independently in the future. Additionally, PLOS ONE offers an option to publish peer-reviewed clinical study protocols. Read more information on sharing protocols at https://plos.org/protocols?utm_medium=editorial-email&utm_source=authorletters&utm_campaign=protocols

# Author comment: Author response to Decision Letter 2

**DOI:** 10.1371/journal.pcbi.1014807.r004

3 Sep 2026

Attachment Submitted filename: fibers_article_PLOS_Review2.pdf

# Editor report: Decision Letter 2

**DOI:** 10.1371/journal.pcbi.1014807.r005

**Authors:** Stacey D. Finley, Haibo Ni

7 Sep 2026

Dear Dr. Regazzoni,

We are pleased to inform you that your manuscript 'The functional impact of myofiber macroscopic organization and disarray in computational models of the murine heart' has been provisionally accepted for publication in PLOS Computational Biology.

Before your manuscript can be formally accepted you will need to complete some formatting changes, which you will receive in a follow up email. A member of our team will be in touch with a set of requests.

Please note that your manuscript will not be scheduled for publication until you have made the required changes, so a swift response is appreciated.

IMPORTANT: The editorial review process is now complete. PLOS will only permit corrections to spelling, formatting or significant scientific errors from this point onwards. Requests for major changes, or any which affect the scientific understanding of your work, will cause delays to the publication date of your manuscript.

Should you, your institution's press office or the journal office choose to press release your paper, you will automatically be opted out of early publication. We ask that you notify us now if you or your institution is planning to press release the article. All press must be co-ordinated with PLOS.

Thank you again for supporting Open Access publishing; we are looking forward to publishing your work in PLOS Computational Biology.

Best regards,

Haibo Ni

Academic Editor

PLOS Computational Biology

Stacey Finley

Section Editor

PLOS Computational Biology

***********************************************************

All issues addressed. Thank you.

# Editor report: Acceptance letter

**DOI:** 10.1371/journal.pcbi.1014807.r006

**Authors:** Stacey D. Finley, Haibo Ni

PCOMPBIOL-D-26-00721R2

The functional impact of myofiber macroscopic organization and disarray in computational models of the murine heart

Dear Dr Regazzoni,

I am pleased to inform you that your manuscript has been formally accepted for publication in PLOS Computational Biology. Your manuscript is now with our production department and you will be notified of the publication date in due course.

The corresponding author will soon be receiving a typeset proof for review, to ensure errors have not been introduced during production. Please review the PDF proof of your manuscript carefully, as this is the last chance to correct any errors. Please note that major changes, or those which affect the scientific understanding of the work, will likely cause delays to the publication date of your manuscript.

Soon after your final files are uploaded, unless you have opted out, the early version of your manuscript will be published online. The date of the early version will be your article's publication date. The final article will be published to the same URL, and all versions of the paper will be accessible to readers.

For Research, Software, and Methods articles, you will receive an invoice from PLOS for your publication fee after your manuscript has reached the completed accept phase. If you receive an email requesting payment before acceptance or for any other service, this may be a phishing scheme. Learn how to identify phishing emails and protect your accounts at https://explore.plos.org/phishing.

Thank you again for supporting PLOS Computational Biology and open-access publishing. We are looking forward to publishing your work!

With kind regards,

Sharmila Kamatchi

PLOS Computational Biology | Carlyle House, Carlyle Road, Cambridge CB4 3DN | United Kingdom ploscompbiol@plos.org | Phone +44 (0) 1223-442824 | ploscompbiol.org | @PLOSCompBiol
